# Suppressing glutamate dehydrogenase guides hematopoietic stem cell divisions into self-renewal

**DOI:** 10.1038/s41467-026-77151-6

**Published:** 2026-09-05

**Authors:** Michihiro Hashimoto, Michie Uchikawa, Yuichiro Arima, Akiyasu Iwase, Ayano Yahagi, Mizuki Ishimaru, Tomomasa Yokomizo, Ayako Nakamura-Ishizu, Yosuke Tanaka, Kenichi Miharada, Hiroki Kurihara, Goro Sashida, Toshio Suda, Terumasa Umemoto

**Affiliations:** 1 Laboratory of Hematopoietic Stem Cell Engineering, International Research Center for Medical Sciences, Kumamoto University, Kumamoto, Japan; 2 Department of Anatomy, Faculty of Life Sciences, Kumamoto University, Kumamoto, Japan; 3 Isotope Science Center, The University of Tokyo, Tokyo, Japan; 4 Department of Microscopic Anatomy and Developmental Biology, Tokyo Women’s Medical University, Tokyo, Japan; 5 Laboratory of Stem Cell Regulation, International Research Center for Medical Sciences, Kumamoto University, Kumamoto, Japan; 6 Laboratory of Proteostasis in Stem Cell, International Research Center for Medical Sciences, Kumamoto University, Kumamoto, Japan; 7 Laboratory of Multicellular dynamics, International Research Center for Medical Sciences, Kumamoto University, Kumamoto, Japan; 8 Laboratory of Transcriptional Regulation in Leukemogenesis, International Research Center for Medical Sciences, Kumamoto University, Kumamoto, Japan; 9 Institute of Hematology, Blood Diseases Hospital, Chinese Academy of Medical Sciences & Peking Union Medical College, Tianjin, China; 10 Research & Investigation Department, Project Division, the Chemo-Sero-Therapeutic Research Institute (KAKETSUKEN), Kumamoto, Japan

**Keywords:** Haematopoietic stem cells, Self-renewal, Stem-cell biotechnology

## Abstract

Hematopoietic stem cells (HSCs) self-renew to expand their pool but often differentiate upon division. Thus, it remains unclear how HSCs maintain their stemness during or after division. Here, we show that the suppression of glutamate dehydrogenase (Glud1), a key enzyme responsible for glutamate (Glu) catabolism, supports the maintenance of HSCs during division. We find that self-renewing mouse HSCs during hematopoietic regeneration exhibited lower intracellular Glu levels compared to HSCs differentiating during ex vivo culture. We therefore focus on the negative regulation of Glu catabolic reactions as a candidate factor to maintain HSCs during division, and find that treatment with the Glud1 inhibitor, R162, maintains a subset of HSCs that would otherwise differentiate during division under normal culture conditions. This treatment successfully results in an approximately 500-fold expansion of functional mouse HSCs that maintain expression pattern of conventional stem cell markers over a 30-day culture period. Importantly, this positive effect of Glud1 inhibition is independent of suppressing cell divisions and dependent on robust JAK2-STAT signaling. Overall, our findings propose that Glud1 inhibition, coupled with robust JAK2–STAT signaling, characterizes a functional HSC state during division, enabling ex vivo expansion of mouse HSCs under conventional culture conditions.

## Abstract (web summary)

The authors show that suppressing glutamate dehydrogenase (Glud1) maintains hematopoietic stem cell stemness during division. This approach achieves a 500-fold ex vivo expansion of functional stem cells under standard culture conditions.

## Introduction

Hematopoietic stem cells (HSCs) are capable of both self-renewal and multi-lineage differentiation, which govern the lifelong maintenance of hematopoietic homeostasis. In the steady state, adult HSCs typically maintain an undivided, quiescent state, characterized by low mitochondrial metabolism within the specialized microenvironment of bone marrow (BM), called the “niche”^1–3^. HSC quiescence is widely believed to be crucial for preventing differentiation or aging by suppressing excessive cell divisions. Consequently, cell division is generally regarded as a factor that disrupts HSC maintenance. Indeed, because conventional culture conditions often induce HSC differentiation concurrently with divisions, ex vivo expansion of functional HSCs remains a major challenge, with only a few successful examples relying on specialized media^4^.

Hematopoietic regeneration after myeloablation forces HSCs to emerge from quiescence and actively divide to quickly compensate for lost hematopoietic cells^5^. Specifically, in the early phase during BM regeneration after 5-FU administration, HSCs undergo self-renewing divisions without negatively affecting stem cell features, resulting in functional HSC expansion^6,7^. Under these conditions, the extracellular environment surrounding HSCs, including cytokines and niche factors, is greatly changed, which drives self-renewing divisions and promotes HSC expansion^8–12^. Moreover, the use of polyvinyl alcohol (PVA) instead of serum albumin reportedly allows HSCs to maintain stem cell features after division during 28-day long-term culture, resulting in a more than 200-fold increase in functional HSCs^4^. Thus, cell divisions do not always exert a negative effect on HSC maintenance. However, it remains unclear how stem cell maintenance or cell fate is regulated during HSC divisions.

Glutaminolysis is a well-known process that contributes to energy metabolism and the synthesis of amino acids, thereby promoting cell division^13,14^. It is initiated through the catabolization of glutamine (Gln) to glutamate (Glu) by glutaminases. Subsequently Glu is catabolized to α-ketoglutarate (αKG)—one of the anaplerotic reactions in the TCA cycle—by aminotransferases, such as GOT1 and GOT2, or glutamine dehydrogenase (Glud1). Several reports have shown that glutaminases or aminotransferases contribute to HSC proliferation in response to inflammation stimulation or hematopoietic recovery after BM transplantation, respectively^15,16^. However, it remains unclear whether glutaminolysis affects HSC fate.

In the present study, we focused on the negative regulation of Glu catabolic reactions to identify a key factor to maintain HSCs during divisions, since self-renewing HSCs during hematopoietic regeneration exhibited lower intracellular Glu levels compared to those attenuating stem cell features under ex vivo culture. Notably, we found that Glud1 inhibition allowed HSCs to maintain stem cell features without negatively affecting their divisional activity, even under ex vivo culture that typically induces differentiation into progenitor cells. This successfully enabled an approximately 500-fold expansion of functional HSCs over a 30-day culture. Importantly, the positive effect of Glud1 inhibition on HSC maintenance was deeply dependent on robust JAK2-STAT signaling. Thus, our findings identify a specific functional state—supported by Glud1 inhibition, coupled with robust JAK2-STAT signaling—that preserves HSC identity during division, thereby enabling ex vivo expansion of mouse HSCs under the conventional culture conditions.

## Results

### EPCR^High^ phenotype marks bona fide stem cells in vivo and in vitro

**Fig. 1:**
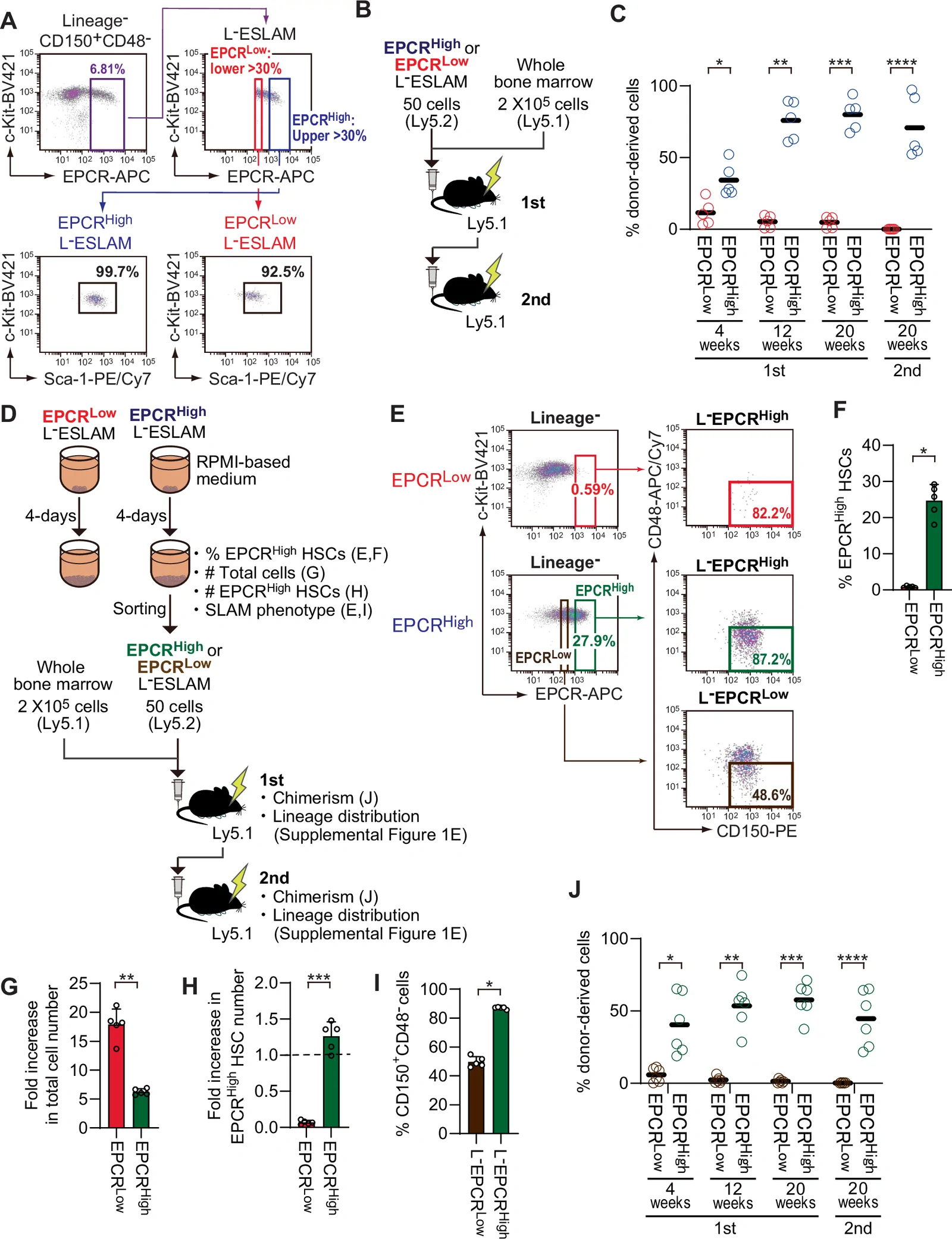
EPCR^High^ fraction contains functional stem cells in the HSC fractions. **A** Gating strategy for EPCR^High^ or EPCR^Low^ cells within the Lineage^-^EPCR^+^CD150^+^CD48^-^ (L^-^ESLAM) HSC fraction. EPCR^High^ and EPCR^Low^ cells are defined as the upper 30% and lower 30% of EPCR fluorescence intensity within the HSC fraction (upper). Sca-1 and c-Kit expression in indicated cells (lower). **B** Competitive transplantation assay using EPCR^High^ and EPCR^Low^ cells from the L^-^ESLAM HSC fraction. Fifty freshly isolated donor cells (Ly5.2) were transplanted into lethally irradiated Ly5.1 mice along with 2 × 10^5^ competitor cells (1st). Subsequently, 1 × 10^6^ BM cells from primary recipients were transplanted into secondary irradiated recipients (2nd). **C** Donor cell chimerism in peripheral blood (PB) after primary (1st) or secondary (2nd) transplantation. Data are means ± S.D. (*n* = 5 biological replicates, two independent experiments; *p = 0.0080, **p = 0.000004, ***p < 0.000001, ****p = 0.000086 using a two-tailed unpaired *t-*test). **D** Strategy to analyze cells after 4-days culture of EPCR^High^ and EPCR^Low^ cells from the L^-^ESLAM HSC fraction in RPMI-based medium. **E** EPCR, CD150 and CD48 expression within the indicated gate after culture. **F–H** Frequency of EPCR^High^ L^-^ESLAM HSCs (**F**), total cell number (**G**), and fold increase in EPCR^High^ HSC number (H) after a 4-day culture. Data are means ± S.D. (*n* = 5 biological replicates, two independent experiments; *p = 0.0003, **p = 0.0005, ***p = 0.0002 using the two-tailed unpaired *t*-test). **I** CD150^+^CD48^-^ cell frequency within lineage^-^ (L^-^) EPCR^High^ or L^-^EPCR^Low^ gate after a 4-day culture of EPCR^High^ HSCs. Data are means ± S.D. (*n* = 5 biological replicates, two independent experiments; *p < 0.0001 using the two-tailed unpaired *t-*test). **J** Donor cell chimerism in PB after primary (1st) and secondary (2nd) transplantation. Donor cells (50 EPCR^High^ or EPCR^Low^ L^-^ESLAM cells) were obtained after a 4-day culture of EPCR^High^ L^-^ESLAM cells before primary transplantation. Secondary transplantation used 1×10^6^ BM cells derived from primary recipients. Data are means ± S.D. (*n* = 6 biological replicates, two independent experiments; *p = 0.0025, **p = 0.000011, ***p < 0.000001, ****p = 0.00021, using the two-tailed unpaired *t*-test).

Our previous study showed that cells highly expressing Endothelial Protein C Receptor (EPCR), an HSC marker, rather than EPCR^Low^ cells within the HSC fraction in the steady state, undergo self-renewing divisions in the early phase after 5-FU administration^7^. Indeed, within the Lineage^-^EPCR^+^CD150^+^CD48^-^ (L^-^ESLAM) HSC fraction which enables the accurate identification of functional HSCs during both the steady state and BM regeneration^6,7^, EPCR^High^ cells (top 30% of the EPCR expression level) expressed both Sca-1 and c-Kit (conventional HSC markers) and exhibited much higher multi-lineage reconstituting activity than EPCR^Low^ cells (lower 30%) after primary and secondary transplantation (Fig. 1A–C and Supplementary Fig. 1A, B), similarly to previous studies^7,17–19^. Notably, EPCR^High^ L^-^ESLAM cells generated EPCR^Low^ L-ESLAM cells after transplantation and during ex vivo culture, but EPCR^Low^ cells provided few EPCR^High^ cells (Fig. 1D–H and Supplementary Fig. 1C, D). In addition, after a 4-day culture of EPCR^High^ L^-^ESLAM cells, most of the cells maintaining the EPCR^High^ phenotype also retained the CD150^+^CD48^-^ HSC phenotype, while approximately half of newly generated EPCR^Low^ cells lost this stem phenotype (Fig. 1E, I). Moreover, EPCR^High^ L^-^ESLAM cells obtained after a 4-day culture maintained long-term engraftment capacity, but newly generated EPCR^Low^ cells did not (Fig. 1D, E, J and Supplementary Fig. 1E). These results suggested that EPCR^High^ cells occupied a higher hierarchy than EPCR^Low^ cells within the L^-^ESLAM HSC fraction in vivo, as well as in vitro. Furthermore, gene expression patterns between EPCR^High^ and EPCR^Low^ cells within the L^-^ESLAM HSC fraction were clearly distinguishable in the steady state, and this discrimination persisted even after 1 day of culture (Supplementary Fig. 2A, B). The 4-day culture of EPCR^High^ L^-^ESLAM cells showed a similar difference between maintained EPCR^High^ and newly generated EPCR^Low^ L^-^ESLAM cells (Supplementary Fig. 2C). EPCR^High^ L^-^ESLAM cells obtained after 4 days of culture maintained the features of the EPCR^High^ L^-^ESLAM cells (shared between the steady state and after a 1-day culture) in terms of gene expression, as well as chromatin accessibility (Supplementary Fig. 2D–K). Chromatin opening and closing regions featured in EPCR^High^ L^-^ESLAM cells tended to be enriched within the locus of genes that were upregulated and downregulated in these cells obtained after 4 days of culture, respectively (Supplementary Fig. 2L, M). In contrast, the newly generated EPCR^Low^ fraction lost these EPCR^High^ features and acquired EPCR^Low^ features (Supplementary Fig. 2D–K). These data indicated that the transformation from EPCR^High^ to EPCR^Low^ cells within the L^-^ESLAM HSC fraction was accompanied by the loss of not only stem cell function but also transcriptomic and epigenetic features. Therefore, this transformation may reflect an earlier step in HSC differentiation compared with the conversion from conventional HSCs to multipotent progenitors.

### Self-renewing divisions in HSCs are driven under low intracellular Glu levels

**Fig. 2:**
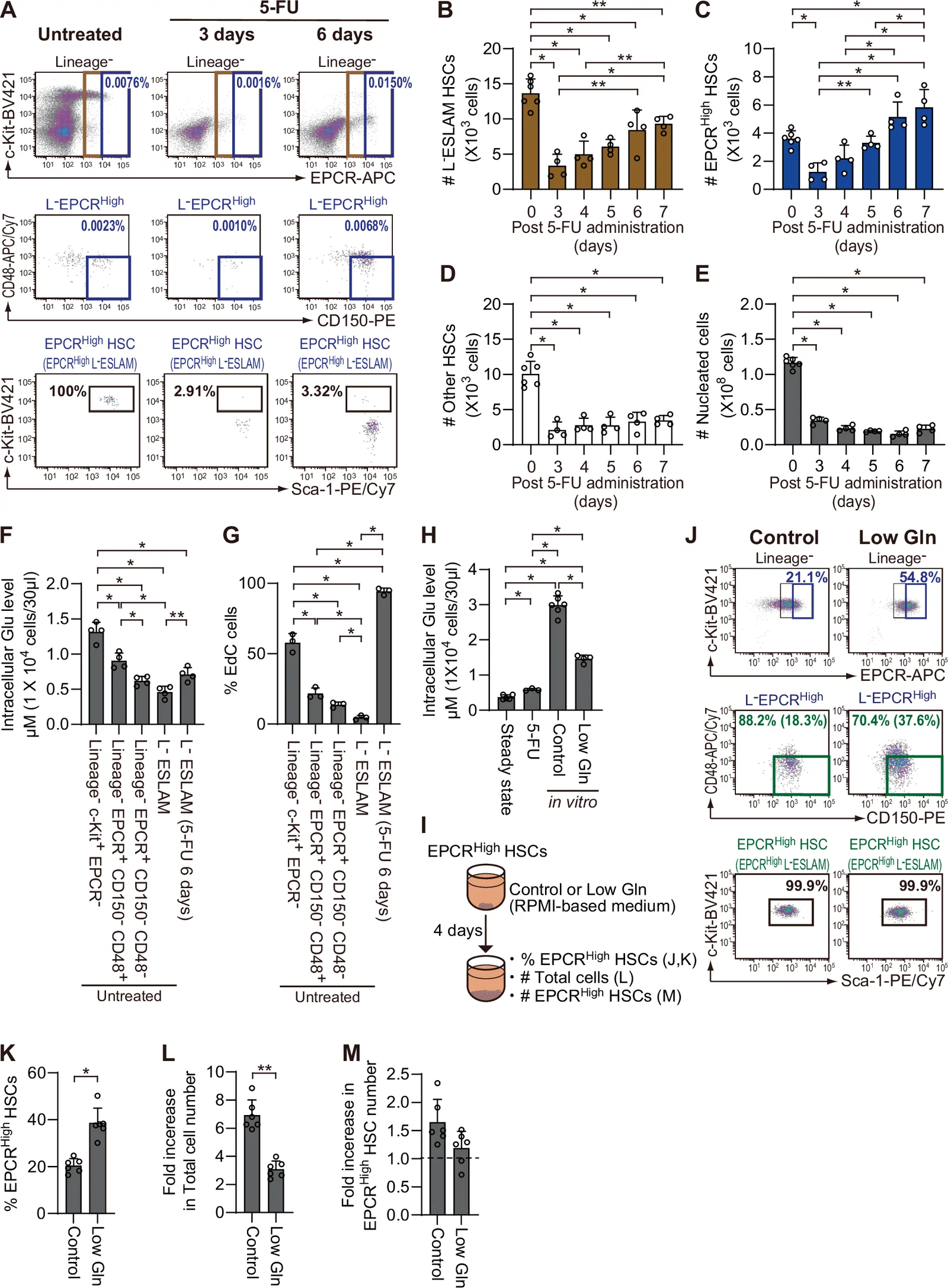
Self-renewing divisions during BM regeneration are driven under lower intracellular Glu level. **A** Gating strategy for total HSCs (L^-^ESLAM HSCs) and EPCR^High^ HSCs in the steady state or after 5-FU administration. Navy or brown gate in upper dot plots represent the EPCR^High^ and EPCR^+^ fractions, respectively. Numbers indicate percentages within BM cells (upper, middle) or within the EPCR^High^ HSC fraction (lower). **B–E** Absolute number of total HSCs (**B**), EPCR^High^ HSCs (**C**), non-EPCR^High^ HSCs (**D**) and total nucleated cells (**E**) after 5-FU administration. Data are means ± S.D. (*n* = 6 biological replicates for control and *n* = 4 for each 5-FU-treated group, two independent experiments; *p < 0.01, **p < 0.05 using one-way ANOVA with Tukey’s test; exact *P* values in Source Data). **F**,**G** Intracellular Glu levels (**F**) and EdC uptake for 24 h (**G**) in HSCs or indicated progenitor cells in the steady state (Untreated) or after 5-FU administration. Data are means ± S.D. (*n* = 4 biological replicates for Intracellular Glu levels, *n* = 3 for EdC uptake, two independent experiments; *p < 0.01, **p < 0.05 using one-way ANOVA with Tukey’s test; exact *P* values in Source Data). **H** Intracellular Glu level in HSCs in the steady state, after 5-FU administration or after a 1-day culture in RPMI-based medium (in vitro) under conventional (Control) and low glutamine (Low Gln: 1% of the standard) conditions. Data are means ± S.D. (*n* = 5 biological replicates for steady state, *n* = 3 for 5-FU, *n* = 6 for Control and *n* = 4 for Low Gln, two independent experiments; *p < 0.01 using one-way ANOVA with Tukey’s test; exact *P* values in Source Data). **I** Strategy for analyzing the effect of decreased intracellular Glu via low Gln conditions in RPMI-based medium. **J–M** Frequency of EPCR^High^ HSCs (**J**,**K**), total cell number (**L**), and EPCR^High^ HSC number (**M**) after a 4-day culture. Percentages indicate whole cultured cells (parentheses) or gates. Data are means ± S.D. (*n* = 6 biological replicates, two independent experiments; *p = 0.0001, **p < 0.0001 using the two-tailed unpaired *t-*test).

Focusing on the EPCR^High^ phenotype as a marker to more precisely identify functional stem cells, ex vivo culture of EPCR^High^ L^-^ESLAM cells (EPCR^High^ HSCs) under the conventional conditions resulted in approximately 6-fold increase in total cell number for 4 days, but EPCR^High^ HSCs themselves could not be effectively expanded (approximately 1.2-fold increase; Fig. 1H). In contrast, 3 to 7 days after 5-FU administration, the number of EPCR^High^ HSCs increased approximately 6-fold for 4 days, whereas other HSCs and the total nucleated cell number within the BM was not (Fig. 2A–E and Supplementary Fig. 3). To identify a key factor for EPCR^High^ HSC expansion, we focused on the low intracellular Glu levels observed in self-renewing HSCs after 5-FU administration. Previously, we found that HSCs dividing after 5-FU administration had higher intracellular Glu levels than quiescent HSCs in the steady state^7^, but much lower levels than Lineage^-^ c-Kit^+^ EPCR^-^ cells in the steady state (Fig. 2F). Nevertheless, HSCs after 5-FU administration showed much higher EdC uptake than Lineage^-^ c-Kit^+^ EPCR^-^ cells in the steady state (Fig. 2G and Supplementary Fig. 4A, B). In addition, ex vivo culture under conventional conditions significantly enhanced the intracellular Glu levels in HSCs (Fig. 2H). These results suggest that self-renewing division was driven under lower intracellular Glu levels than progenitor cell divisions or HSC divisions accompanied by loss of EPCR^High^ phenotype.

### Glud1 inhibition contributes to the maintenance of stem cell features during HSC divisions

**Fig. 3:**
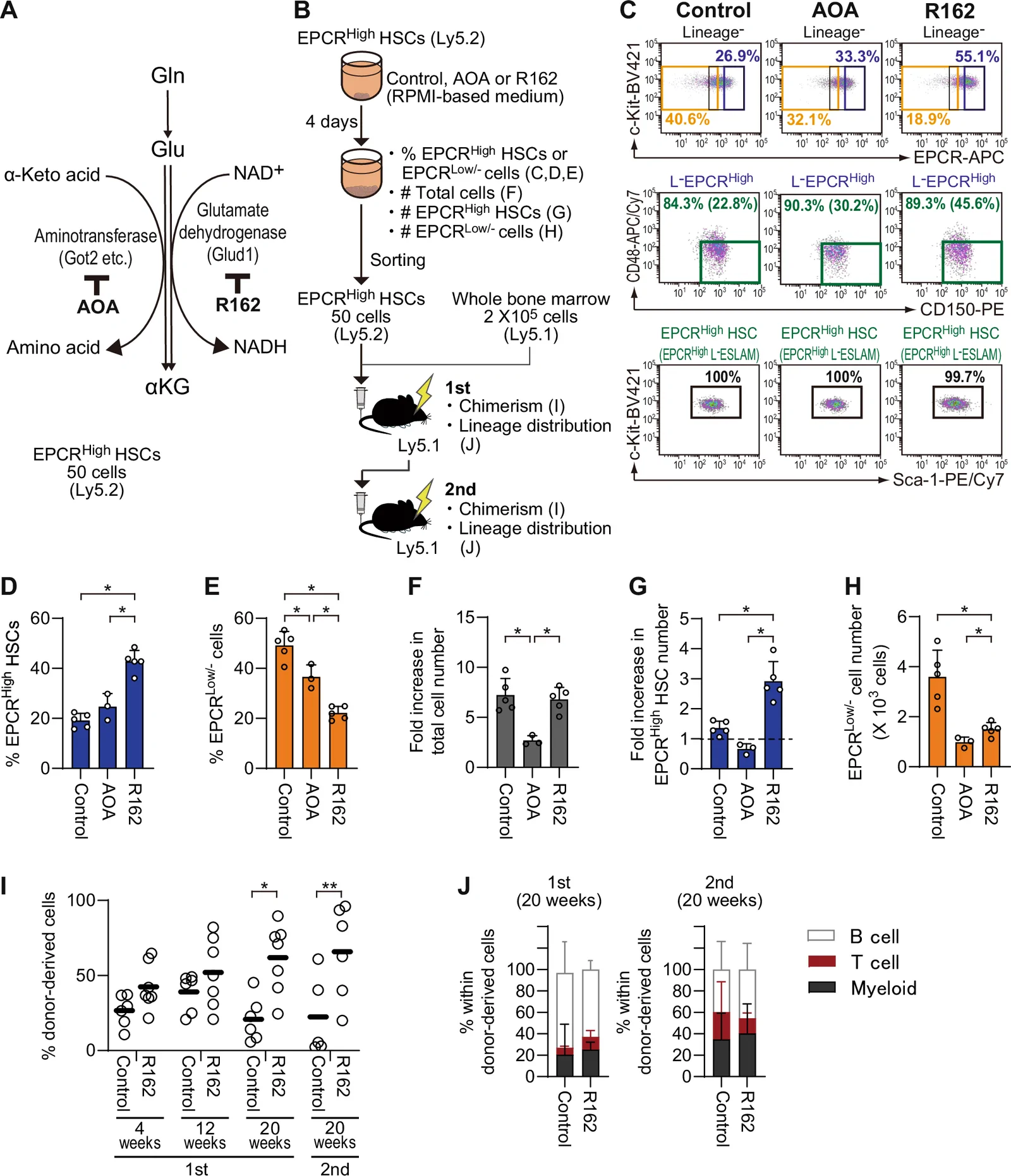
Glud1 inhibition contributes to the maintenance of EPCR^High^ HSCs without affecting HSC divisions. **A** Glutamate catabolic reactions and their corresponding inhibitors. Glutamine (Gln) is converted to Glutamate (Glu), which is then catabolized to α-Ketoglutarate (αKG) by Aminotransferases (e.g., GOT1, GOT2, inhibited by AOA) or Glutamate dehydrogenase (Glud1, inhibited by R162). **B** Strategy for analyzing the effect of inhibiting Glu catabolic reactions on HSCs during ex vivo culture. EPCR^High^ HSCs (EPCR^High^ L^-^ESLAM cells) were cultured for 4 days under Control, AOA, or R162 conditions in RPMI-based medium. **C** The gating strategy for EPCR^High^ HSCs (EPCR^High^ L^-^ESLAM HSCs) and EPCR^Low/-^ cells after 4-day culture. Navy, black or orange gates in upper dot plots represent the EPCR^High^, EPCR^+^ or EPCR^Low/-^ fraction, respectively. Numbers or numbers within parentheses in the dot plots represent the percentage within the indicated gate or whole culture cells, respectively. **D–H** Frequencies of EPCR^High^ HSCs (**D**), EPCR^Low/-^ cells (**E**) and absolute numbers of total cells (**F**), EPCR^High^ HSCs (**G**) and EPCR^Low/-^ cells (**H**)after a 4-day culture of EPCR^High^ HSCs under the indicated conditions. Data are means ± S.D. (*n* = 5 biological replicates for Control, *n* = 3 for AOA and *n* = 5 for R162, two independent experiments; *p < 0.01, **p < 0.05 using one-way ANOVA with Tukey’s test; exact *P* values in Source Data). **I**, **J** Chimerism of donor-derived cells in peripheral blood (PB) (**I**) and their lineage distribution within donor-derived cells (**J**) after primary (1st) and secondary (2nd) transplantation. Fifty EPCR^High^ donor HSCs were obtained after a 4-day culture of EPCR^High^ HSCs in the absence (Control) or presence of R162 before primary transplantation. For secondary transplantation, 1 × 10^6^ BM cells derived from the primary recipients were used. Data are means ± S.D. (*n* = 6 biological recipients for Control and *n* = 7 for R162 in the primary transplantation; *n* = 5 for Control and *n* = 6 for R162 in the secondary transplantation, two independent experiments; *p = 0.0032, **p = 0.036 using the two-tailed unpaired *t-*test).

Next, to investigate the effects of low intracellular Glu levels on HSC divisions, we employed low glutamine (Gln) conditions (1% of the standard Gln concentration), which reduced intracellular Glu levels (Fig. 2I). After EPCR^High^ HSCs were cultured for 4 days, low Gln conditions more efficiently maintained the frequency of EPCR^High^ HSCs compared to control conditions, but decreased total cell number (Fig. 2J–M). To confirm whether these effects are mediated by suppressing Glu-catabolic reactions, we utilized inhibitors targeting enzymes responsible for the catabolism of Glu to α-ketoglutarate, namely aminotransferases (e.g., GOT1, GOT2, etc.) and Glud1, respectively (Fig. 3A). The inhibition of aminotransferases using aminooxyacetic acid (AOA) hardly affected the frequency of EPCR^High^ HSCs after 4 days of culture but significantly reduced total cell numbers (Fig. 3B–F). Among amino acids generated by Glu-dependent aminotransferases, only intracellular aspartate (Asp) levels increased during the culture under control conditions and were significantly suppressed by both AOA treatment and low Gln conditions (Supplementary Fig. 4C–E). These findings suggest that intracellular Glu supported HSC division through Asp synthesis. In contrast, treatment with R162, an inhibitor for Glud1, did not impact total cell numbers, but more effectively maintained the frequency of EPCR^High^ HSCs compared to control conditions (Fig. 3B–F). Thus, inhibiting Glud1 or aminotransferases selectively mimicked the effects of low Gln conditions on HSC maintenance and division, respectively. Notably, R162 treatment resulted in an increase in the absolute number of EPCR ^High^ HSCs and concurrently decreased that of EPCR^Low/-^ cells after 4 days of culture compared to control conditions (Fig. 3G, H). This indicates that Glud1 inhibition shifts the frequency of these populations, favoring EPCR ^High^ HSCs while reducing EPCR^Low/-^ cells without reducing total cell numbers. Moreover, *Glud1* knockout using CRISPR/Cas9 recapitulated the effect of R162 on HSC maintenance in vitro (Supplementary Fig. 4F–K). Furthermore, R162-expanded HSCs showed higher long-term engraftment activity than control HSCs in serial transplantation assay (Fig. 3I, J). Thus, Glud1 inhibition increased the functional HSC number by more effectively maintaining functional EPCR^High^ HSCs without negatively affecting their divisional activity during ex vivo culture.

### Robust JAK2 activation is needed for Glud1 inhibition to maintain HSCs

Consistent with Glud1-mediated Glu catabolism involving both αKG generation and NAD^+^ consumption, R162 decreased the αKG level and increased the ratio of NAD^+^/NADH in HSCs (Supplementary Fig. 5A, B). These metabolic changes may be involved in Glud1-mediated HSC maintenance. However, while the addition of dimethyl-αKG (dmαKG), a cell-permeable analog of αKG, successfully rescued the decrease in intracellular αKG level induced by R162 (Supplementary Fig. 5A), it failed to abolish the positive effect of Glud1 inhibition on HSC maintenance (Supplementary Fig. 5C–G). Moreover, although the supplementation of NR, a NAD^+^ precursor, significantly increased the NAD^+^/NADH ratio in HSCs (Supplementary Fig. 5B), its impact on the maintenance of EPCR^High^ HSCs was marginal (Supplementary Fig. 5H–L). These results suggest that an increase in the NAD^+^/NADH ratio alone is insufficient to account for the robust effect of Glud1 inhibition. Collectively, our findings strongly indicated that neither changes in global αKG abundance nor the NAD^+^/NADH ratio was a primary factor in Glud1 inhibition-mediated HSC maintenance.

**Fig. 4:**
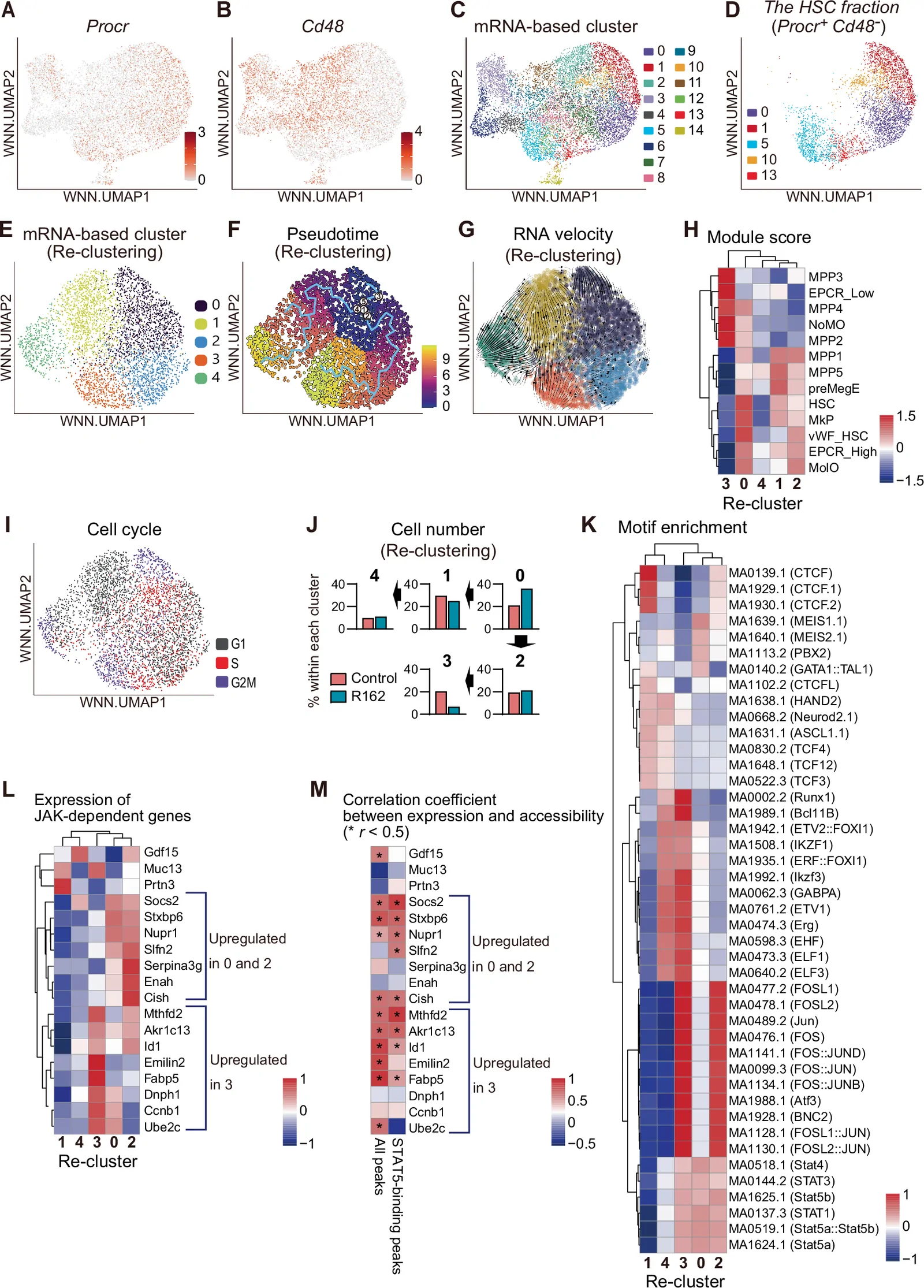
Glud1 inhibition supports HSC maintenance in the context of robust cytokine responses. **A** Expression level of *Procr* (EPCR). **B** Expression level of *Cd48*. **C** Clustering based on gene expression pattern in the UMAP plot. **D** The five clusters exhibiting highest frequency of *Procr*^+^*Cd48*^-^ cells, identified as the HSC fractions (refer to Supplementary Fig. 6D). **E** Re-clustering of the HSC fraction. **F** The pseudotime analysis of the HSC fraction projected onto the UMAP plot. Light blue line and number represent differentiation trajectories and root cell score rankings, respectively. **G** The trajectory analysis of the HSC fraction based on RNA velocity in the UMAP plot. **H** Module scores for genes up-regulated in stem cell or differentiated cells^20–23^ across the five re-clusters. **I** Cell cycle distribution within the HSC fractions. **J** The cell count for each re-cluster. **K** The heatmap represents top 10 of transcription factor binding motifs enriched within opening regions in each re-cluster. **L** Heatmap representing the expression levels of JAK-dependent genes across the re-clusters. **M** Correlation coefficients between gene expression and chromatin accessibility for JAK-dependent genes, calculated across all detected peaks (left) and specifically at potential STAT5-binding peaks (right) (*, *r* > 0.5).

To elucidate the mechanism by which Glud1 inhibition contributed to HSC maintenance, we performed single-cell multiome (scMultiome) analysis using whole cells obtained after EPCR^High^ HSCs were cultured in the absence or presence of R162. Mirroring the flow cytometric results (Fig. 3C–E), the scMultiome data reflected an increased frequency of both *Procr* (EPCR)^+^ cells and *Procr*^+^ *Cd48*^-^ populations following R162 treatment (Fig. 4A–B and Supplementary Fig. 6A–B). Following the identification of 14 distinct transcriptome-based clusters via the Uniform Manifold Approximation and Projection (UMAP), we selected five clusters (0, 1, 5, 10, and 13) that exhibited the highest frequency of *Procr*^+^ *CD48*^-^ cells as the HSC fraction (Fig. 4C, D and Supplementary Fig. 6C, D). Consistent with their identity, these clusters likely contained more cells showing higher module scores of established HSC signatures (EPCR High, MolO^20^ and HSC^21^) and MPP1/5 signatures^21^, while showing lower scores for MPP2/3/4^21^ (Supplementary Fig. 6E–L).

To investigate the effects of R162 within the HSC fraction in greater detail, cells from the five clusters identified as HSCs were re-clustered into distinct ‘re-clusters,’ each characterized by distinct gene expression profiles and chromatin accessibility patterns (Fig. 4E and Supplementary Fig. 6M, N). Notably, unbiased pseudotime analysis identified the top five nodes with the highest root cell scores within re-cluster 0 and revealed two distinct trajectories originating from this re-cluster (Fig. 4F). These pathways were further corroborated by RNA velocity analysis (Fig. 4G). Moreover, re-cluster 0 exhibited the ighest module scores of HSC signatures (e.g., EPCR-High, MolO^20^, vWF HSC^22^, and HSC^21^) (Fig. 4H), confirming it as the most primitive population. These results suggest two differentiating pathways originating from re-cluster 0 (one; 0 → 2 → 3; another 0 → 1 → 4). Interestingly, re-cluster 0, 2, and 3 were significantly enriched for cells presenting S or G2/M phases of the cell cycle compared to other re-clusters (Fig. 4I). Moreover, Re-clusters 1 showed higher scores for pre-MegE^23^ and MkP^22^ signatures compared to re-cluster 2, while re-cluster 3 exhibited significantly higher scores for MPP2/3/4 compared to re-clusters 1 or 4 (Fig. 4H). Importantly, R162 treatment increased cell numbers within re-cluster 0 but decreased those of re-cluster 3 without affecting re-cluster 1 and 4 (Fig. 4J). These data suggest that Glud1 inhibition suppressed the specific differentiation pathway of 0 → 2 → 3 which is characterized by a distinct shift in chromatin structure and gene expression, high proliferative activity, and the transition from primitive HSCs toward MPP2/3/4 state. This finding is consistent with the observed effect of R162 to drive robust HSC expansion during ex vivo culture (Fig. 3).

In addition, the chromatin accessible regions in re-clusters 0, 2, and 3 were significantly enriched for STAT-related binding motifs compared to other re-clusters (Fig. 4K). Consistent with this, re-clusters 0, 2, and 3 exhibited higher expression levels of most JAK-dependent genes than re-clusters 1 and 4 (Fig. 4L). Notably, these JAK-dependent genes were differentially expressed across re-clusters 0, 2, and 3 (Fig. 4L). Importantly, approximately half of these genes showed a strong correlation (*r* > 0.5) between expression levels and chromatin accessibility (Fig. 4M and Supplementary Fig. 6O). Given that our culture system, comprising a combination of Stem Cell Factor (SCF) and Thrombopoietin (TPO), primarily activates STAT5 through TPO signaling^24^, we re-analyzed these correlations by restricting to potential STAT5-binding regions within all detected peaks; this yielded similar results (Fig. 4M and Supplementary Fig. 6P). Importantly, similar to the JAK-dependent genes, HSC signature gene sets (e.g., EPCR High and MolO^20^) and differentiation-associated gene sets (e.g., EPCR Low and NoMO^20^) showed a significant and approximately two-fold enrichment of genes with high expression–accessibility correlation (*r* > 0.5) compared to the genome-wide average (Supplementary Fig. 6Q). This enrichment pattern remained robust even when the analysis was restricted to potential STAT5-binding regions (Supplementary Fig. 6R). Notably, module scores for these HSC and differentiation signatures clearly distinguished the stem-like clusters (re-clusters 0 and 2) from the MPP-like cluster (re-cluster 3) within the differentiation trajectory suppressed by Glud1 inhibition (Fig. 4H, J), despite these clusters sharing a similarly high enrichment of STAT-related motifs (Fig. 4K). These findings suggest that the molecular framework supporting HSC identity relies on the synergy between Glud1-mediated regulation and the active JAK–STAT signaling axis. Based on these observations, we hypothesized that the beneficial effect of R162 on HSC self-renewal and expansion is functionally dependent on robust JAK–STAT activity.

**Fig. 5:**
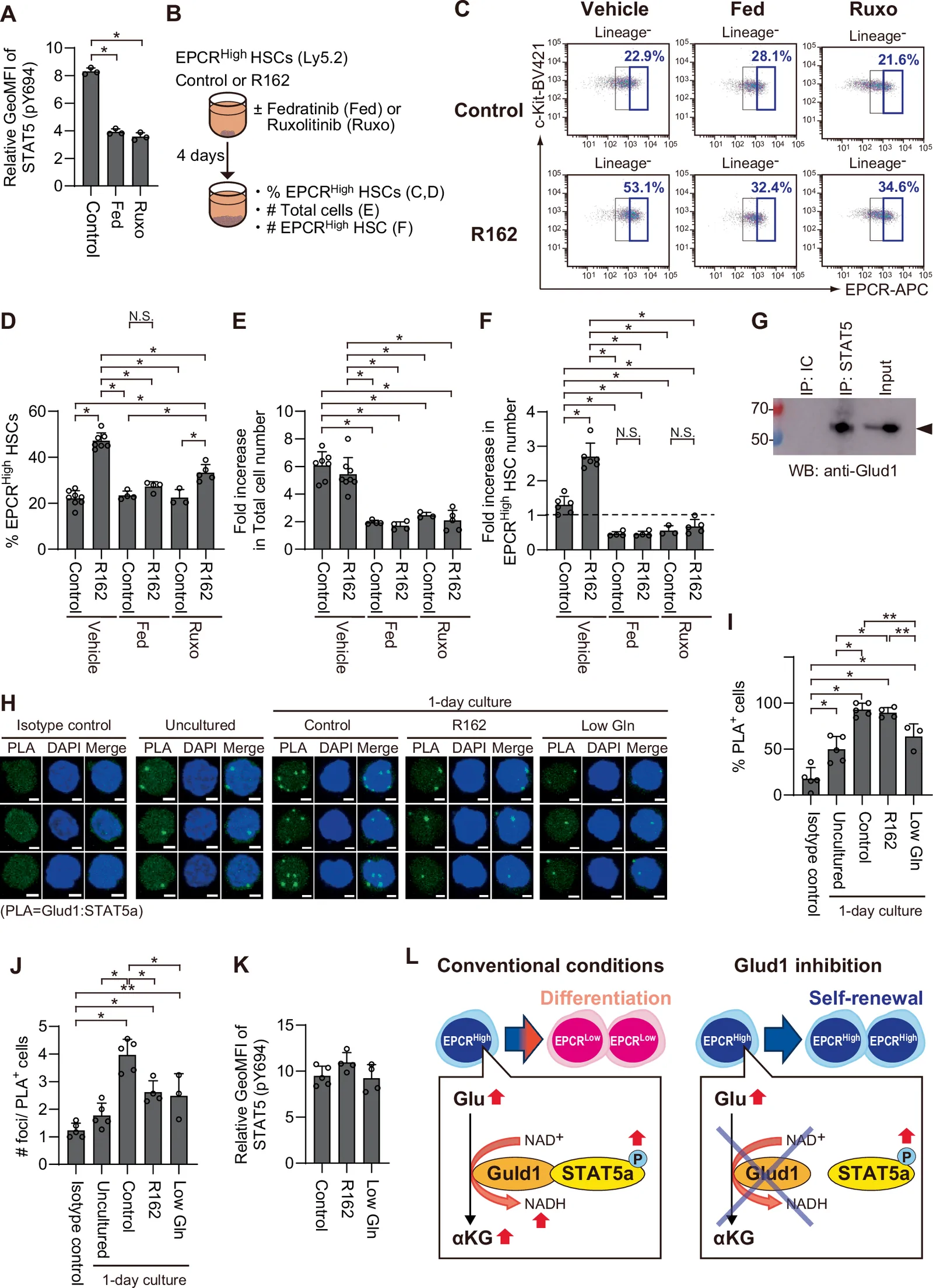
Glud1 inhibition requires robust JAK2 activation to support HSC maintenance. **A** Relative GeoMFI for phosphorylated STAT5 (pY694) in HSCs after a 1-day culture ± fedratinib (Fed) or ruxolitinib (Ruxo). Data are means ± S.D. (*n* = 3 biological replicates, two independent experiments; *p < 0.0001 using one-way ANOVA with Tukey’s test). **B** Strategy to examine JAK2 inhibitor impact on R162-mediated (Glud1 inhibitor) maintenance or expansion of EPCR^High^ HSCs during a 4-day culture. **C–F** EPCR^High^ HSC frequency (**C**,**D**), total cell number (**E**), and EPCR^High^ HSC number (**F**) after culture. Dot plot percentages indicate gates. Data are means ± S.D. (*n* = 6 biological replicates for Vehicle, *n* = 4 biological replicates for all other groups from three independent experiments; *p < 0.01 using one-way ANOVA with Tukey’s test; exact *P* values in Source Data). **G** Western blot (WB) analysis of immunoprecipitation (IP) samples or an input control using anti-Glud1 antibody in A-6 cell lysate. Data are representative of three independent experiments with similar results. **H–J** Nuclei (left), PLA signal of Glud1 and STAT5a (center) and merge (right) in HSCs (H). Scale bar: 2 µm. Frequency of HSCs with foci (**I**) and foci number per PLA-positive cell (**J**). Data are means ± S.D. (*n* = 5 biological replicates for Isotype, Uncultured, Control and R162; *n* = 4 for Low Gln, four independent experiments; *p < 0.01, **p < 0.05 using one-way ANOVA with Tukey’s test; exact *P* values in Source Data). **K** Relative GeoMFI for STAT5 (pY694) in HSCs after a 1-day culture. Data are means ± S.D. (*n* = 5 biological replicates for Control, *n* = 4 for other groups, two independent experiments; *p < 0.01, **p < 0.05 using one-way ANOVA with Tukey’s test). **L** Schematic of Glud1 catabolic activity and JAK–STAT signaling landscape characterizing HSC states. Under the conventional culture, HSCs increase intracellular Glu to support division, which occurs concurrently with differentiation and promotion of Glud1–STAT5a association. Conversely, Glud1 inhibition with R162 suppresses this association, effectively maintaining EPCR^High^ HSCs without compromising divisional activity or STAT5 activation—thereby facilitating “self-renewing divisions”.

To test this hypothesis, we cultured EPCR^High^ HSCs with R162 in the presence of JAK2 inhibitors (fedratinib or ruxolitinib), both of which block STAT activation. Notably, when these JAK2 inhibitors suppressed the phosphorylation of STAT5 (a downstream molecule in JAK2) (Fig. 5A and Supplementary Fig. 7A, B), the positive effect of R162 on maintaining or expanding EPCR^High^ HSCs was significantly suppressed by either JAK2 inhibitor (Fig. 5B–F). In contrast, when we used the Erk1/2 inhibitor SCH772984, which suppressed cell proliferation without inhibiting STAT5 phosphorylation, R162 retained its ability to promote the maintenance and expansion of EPCR^High^ HSCs (Supplementary Fig. 7C–H). These data suggest that robust JAK2 activation, rather than general cell proliferative activity, plays a predominant role in allowing Glud1 inhibition to effectively maintain HSCs, although the potential confounding effects of other parallel cytokine signaling pathways cannot be entirely ruled out.

To further investigate how Glud1 affects JAK–STAT signaling, we focused on its physical interaction with STAT5a^25^. Indeed, we also confirmed the potential of Glud1 to interact with STAT5 in A-6, which is a hematopoietic progenitor cell line by using immunoprecipitation and western blot (Fig. 5G). Proximity ligation assay (PLA) revealed that some HSCs in the steady state exhibited a few foci representing Glud1-STAT5a association (Fig. 5H, I). Interestingly, when ex vivo culture significantly elevated intracellular Glu level in HSCs (Fig. 2F), both the frequency of HSCs exhibiting the foci and the number of foci per PLA-positive cell increased (Fig. 5H–J). However, R162 treatment and low Gln conditions (which decrease intracellular Glu levels (Fig. 2F)) remarkably reduced the foci number per PLA-positive cell (Fig. 5H–J). Importantly, R162 treatment and low Gln conditions did not affect phosphorylation levels of STAT5 in HSCs (Fig. 5K and Supplementary Fig. 7I). These data indicate that Glud1-STAT5a associations were reduced under conditions where the suppression of Glud1-mediated reactions supports HSC maintenance (Fig. 5L). Thus, our results exhibited a distinct HSC state in which the maintenance of self-renewal capacity under Glud1 inhibition is characterized by robust JAK–STAT activity and a catabolism-dependent modulation of the Glud1-STAT5a association.

### Glud1 inhibition enables the effective expansion of functional HSCs under conventional culture conditions

**Fig. 6:**
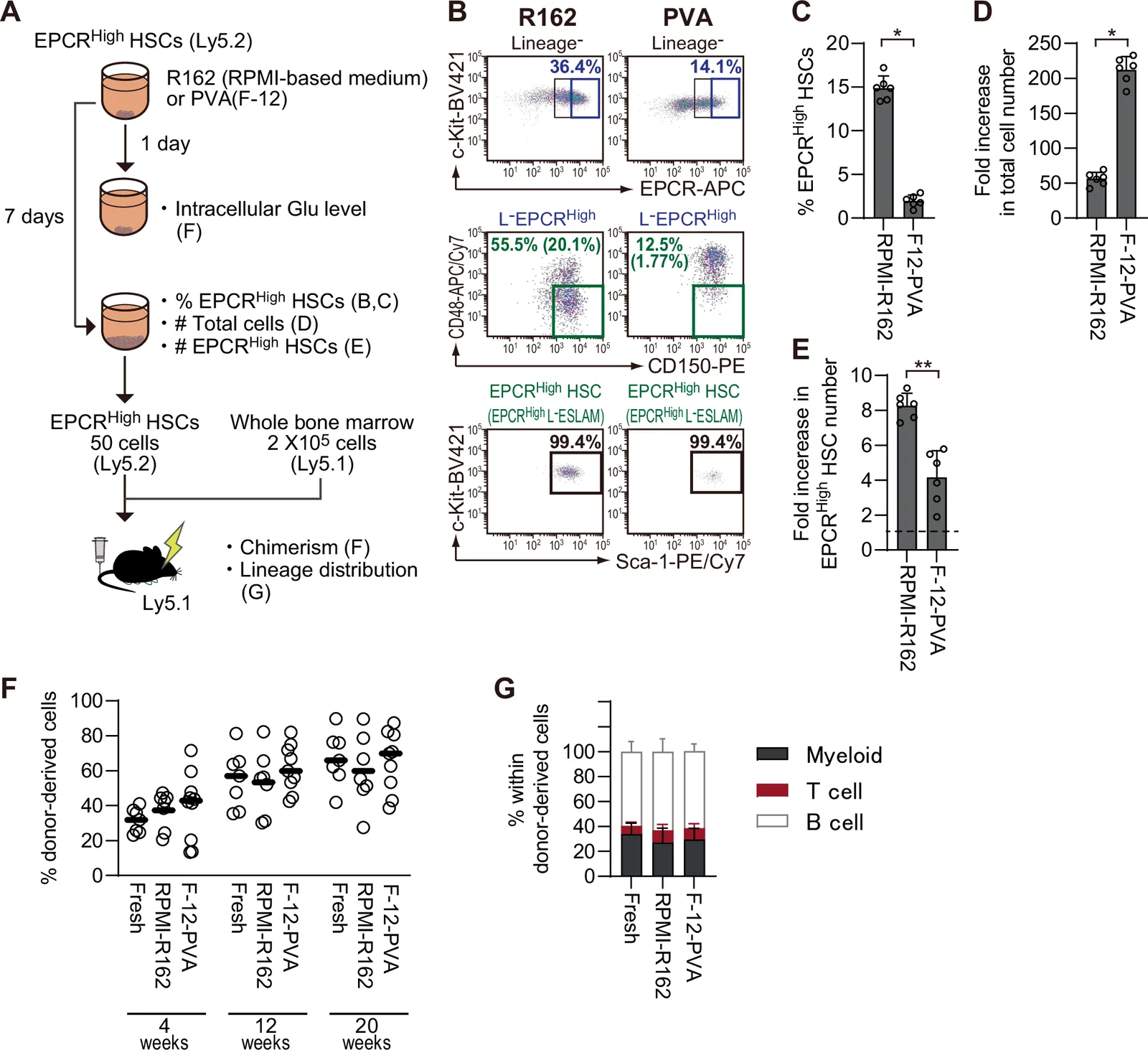
Glud1 inhibition enables more effective ex vivo expansion of HSCs under the conventional culture conditions. **A** Strategy to analyze EPCR^High^ HSCs (EPCR^High^ L^-^ESLAM cells) expanded by R162 treatment in RPMI-based medium or the F12-PVA system for 7 days. **B–E** The frequency of EPCR^High^ HSCs (**B**,**C**), total cell number (**D**), and EPCR^High^ HSC number (**E**) after a 7-day culture of EPCR^High^ HSCs under indicated conditions. Numbers or numbers within parentheses in the dot plots represent the percentage within the indicated gate or whole culture cells, respectively. Data are means ± S.D. (*n* = 4 biological replicates, two independent experiments; *p < 0.0001, **p = 0.0006 using the two-tailed unpaired *t*-test). **F**,**G** Chimerism of donor-derived cells in peripheral blood (PB) after transplantation (**F**). The lineage distribution (**G**) of reconstituted hematopoietic cells within donor-derived cells 20 weeks after transplantation. Data are means ± S.D. (*n* = 7 biological replicates, two independent experiments, no significant difference by one-way ANOVA with Tukey’s test).

Next, we further examined the potential of Glud1 inhibition for ex vivo expansion of HSCs. Interestingly, the R162 Glud1 inhibitor continued to expand EPCR^High^ HSCs even when the culture period was extended by 7 days, whereas the control conditions were not effective (Supplementary Fig. 8). Moreover, R162 yielded an approximately 2-fold greater number of EPCR^High^ HSCs over 7 days of culture compared to the F12-PVA system, which is regarded as a gold standard culture condition for HSC expansion (Fig. 6A–E)^4^. Indeed, functional HSCs expanded by the F12-PVA system are also primarily enriched within the EPCR^High^ CD48^-^ population of SLAM LSK fraction^19^. Importantly, 50 EPCR^High^ HSCs expanded under these culture conditions, when transplanted, showed similar engraftment activity that was comparable with freshly uncultured HSCs (Fig. 6F, G). These data suggest that Glud1 inhibition resulted in a more effective expansion of EPCR^High^ HSCs compared to the F12-PVA system, particularly over the 7-day culture period.

**Fig. 7:**
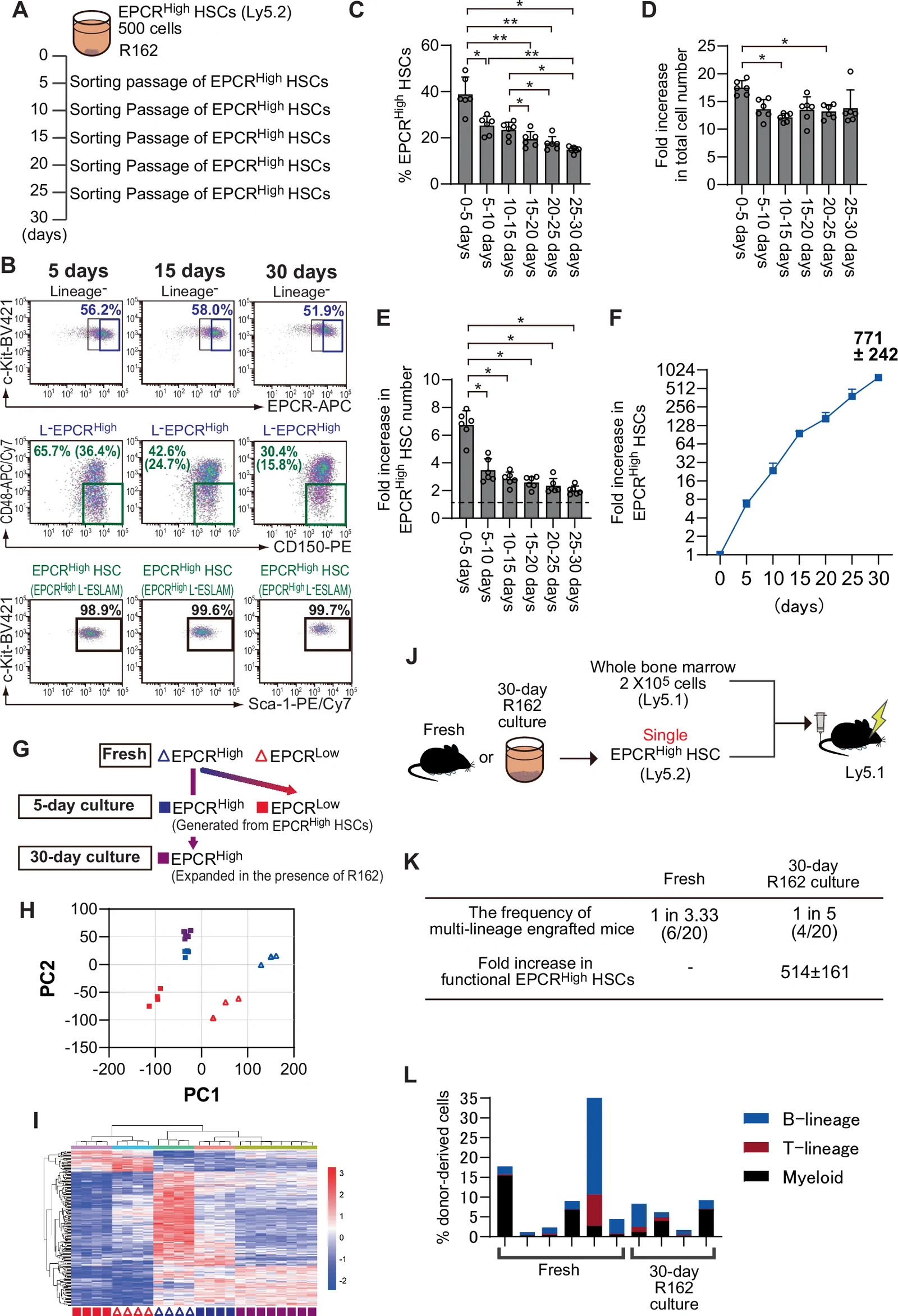
Glud1 inhibition enables long-term maintenance and expansion of HSCs under the conventional culture conditions. **A–F** After the primary culture of 500 EPCR^High^ HSCs (EPCR^High^ L^-^ESLAM cells) with R162 in RPMI-based medium, 500 of those HSCs were sorted and passaged every 5 days or a total of 30 days (**A**). EPCR^High^ HSC frequency (**B**,**C**), total cell number (**D**) or EPCR^High^ HSC number (**E**) after primary or every passage culture. Numbers or numbers within parentheses in the dot plots represent the percentage within the indicated gate or whole culture cells, respectively. Estimated expansion efficiency of phenotypic EPCR^High^ HSCs over 30-day passage culture (**F**). Numbers in the graph represent the fold increase in phenotypic EPCR^High^ HSCs over 30-day culture Data are means ± S.D. (*n* = 6 biological replicates, three independent experiments, *p < 0.01, **p < 0.05 using one-way ANOVA with Tukey’s test; exact *P* values in Source Data). **G–I** Sample information for EPCR^High^ HSCs or EPCR^Low^ (EPCR^Low^ L^-^ESLAM) cells in the steady state, obtained after a 5-day or 30-day culture of EPCR^High^ HSCs (**G**). The principal component analysis (PCA) plot for EPCR^High^ HSCs or EPCR^Low^ cells in the steady state or after a 5-day or 30-day culture, based on RNA-seq analyses (**H**). The heatmap of hierarchical clustering analysis for EPCR^High^ HSCs or EPCR^Low^ cells in the steady state or obtained after a 5-day or 30-day culture of EPCR^High^ HSCs, based on RNA-seq analyses (**I**). **J–L** Single-cell transplantation of EPCR^High^ HSC in the steady state or obtained after a 30-day culture into lethally irradiated mice along with competitor cells (**J**). The frequency of recipient mice showing multi-lineage (myeloid, T and B lineage) reconstitution 16 weeks after the transplantation (**K**). The fold increase in functional EPCR^High^ HSCs was calculated from these reconstitution frequencies and the expansion efficiency (referred to Fig. 7F). Chimerism and lineage distribution of donor-derived cells in the peripheral blood of each recipient (**L**). (Two independent experiments).

Next, to further examine the positive effect of HSC maintenance, we performed a 30-day long-term culture of HSCs in the presence of R162. During the 30-day culture, EPCR^High^ HSCs were maintained by cell sorting and passaging every 5 days (Fig. 7A). Indeed, each 5-day passage culture maintained EPCR^High^ HSCs at 15 to 25% of the total obtained cells, yielding an approximately 15-fold increase in total cell number (Fig. 7B–D). Therefore, a 2- to 3-fold increase in the number of EPCR^High^ HSCs continued with each 5-day passage culture, eventually estimating a more than 700-fold increase in EPCR^High^ HSCs (Fig. 7E, F). Importantly, Principal Component Analysis (PCA) following RNA-seq demonstrated that EPCR^High^ HSCs expanded over 30 days (30D EPCR^High^ HSCs) still maintained higher PC2 values, a characteristic shared with both EPCR^High^ HSCs in the steady state and those maintained after a 5-day culture (Fig. 7G, H). Furthermore, when focusing on EPCR^High^-featured genes, 30D EPCR^High^ HSCs showed a similar gene expression pattern compared to EPCR^High^ HSCs generated after a 5-day culture (Fig. 7I). These data suggest that EPCR^High^ HSCs, when expanded over a 30-day long-term culture under Glud1 inhibition, maintained a stem cell-like gene expression profile.

Finally, we validated the frequency of functional stem cells within 30D EPCR^High^ HSC using single cell transplantation. The transplantation of a single, freshly uncultured EPCR^High^ HSC resulted in 30% (6/20) of recipients achieving multi-lineage reconstitution, while a single 30D EPCR^High^ HSC enabled 20% (4/20) of recipients to do so (Fig. 7K, L). Given these frequencies of engraftable stem cells within each EPCR^High^ HSC fraction, R162 treatment achieved an approximately 770-fold increase in phenotypic EPCR^High^ HSCs after a 30-day culture, which calculatedly corresponds to an approximately 500-fold increase in functional HSCs. Thus, our findings indicate that Glud1 inhibition is a core factor enabling the long-term ex vivo maintenance and expansion of HSCs under conventional culture conditions.

## Discussion

Herein, we focused on the lower intracellular Glu levels observed in self-renewing HSCs during BM regeneration. We then discovered that Glud1 inhibition enabled the ex vivo expansion of HSCs by robustly maintaining the frequency of EPCR^High^ HSCs without altering their proliferation activity during ex vivo culture (Fig. 3). Importantly, the Glud1 inhibitor R162 increased the number of cells belonging to the most primitive cluster (Re-cluster 0) in parallel with a decrease in cells along a specific differentiation trajectory (Re-clusters 0 → 2 → 3) characterized by robust STAT activity (Fig. 4E–K). These findings indicate that HSC expansion driven by Glud1 inhibition is attributed to the active preservation of HSCs that would otherwise undergo differentiation under robust STAT activity, rather than merely modulating general cell division. In sharp contrast, Glud1 inhibition did not affect the number of cells along the alternative differentiation pathway (Re-clusters 0 → 1 → 4) characterized by relatively lower STAT activity (Fig. 4E–K). Given that the beneficial effect of Glud1 inhibition on HSC maintenance is dependent on robust JAK2-STAT signaling (Fig. 5A–F), these parallel datasets give rise to the possibility that Glud1 inhibition preferentially acts upon or expands a specific HSC subset that inherently maintains high JAK–STAT activity throughout the culture. Simultaneously, the reduced EPCR^low/-^ pool upon R162 treatment (Fig. 3H) may reflect a distinct compositional bias resulting from the relative preservation of cells on these alternative branches (Re-clusters 1 and 4), rather than a generalized suppression of all committed progenitors. Collectively, these insights indicate that Glud1 inhibition actively guides HSCs into self-renewal under robust STAT activity by blocking the specific differentiation trajectory and may shape the distinct progenitor landscape through this trajectory-specific bottleneck.

While Glud1 inhibition did not affect total cell number during the 4-day culture (Fig. 3), cultures extending beyond 5 days showed a negative effect of R162 treatment on total cell number (Supplementary Fig. 7D). Importantly, EPCR^High^ HSCs inherently exhibit significantly lower divisional activity compared to EPCR^Low^ cells during culture^7^. Therefore, the negative effect of R162 on total cell number observed after 5 days or later appears to be attributable to the higher frequency of EPCR^High^ HSCs rather than a direct impact of Glud1 inhibition.

A recent report shows that the F12-PVA system employs PVA instead of serum albumin, enabling a more than 200-fold expansion of functional HSCs over a 28-day culture^4^. Indeed, the F12-PVA conditions appear to expand HSCs after 7-day culture mainly by increasing total cell number rather than the frequency of EPCR^High^ HSCs, whereas R162 treatment did the opposite (Fig. 6A–E). This suggests that Glud1 inhibition facilitates HSC expansion through a mechanism distinct from that of PVA. Therefore, combining R162 with the PVA system may enable a more effective expansion of HSCs. However, we could not test this possibility, because R162 compound did not dissolve in the F12-PVA medium, likely due to the absence of serum albumin. Screening for a suitable solvent will thus be required to combine these culture systems.

Although R162, a Glud1 inhibitor, reduced the αKG abundance and increased the NAD^+^/NADH ratio in HSCs, simply modifying the outputs of Glud1-related metabolism failed to either cancel or recapitulate the positive effect of R162 on HSC maintenance (Fig. S4). These data strongly indicate that such global metabolic shifts or their associated systemic responses are independent of the primary mechanism through which Glud1 inhibition maintains HSCs. However, these observations do not exclude the possibility that Glu catabolism suppressed by Glud1 inhibition may affect HSC regulation through a localized or complex-dependent mechanism. Reportedly, Glud1 physically interacts with the NF-κB complex, and Glud1-generated αKG binds to and activates IKKβ, which could not be mimicked by simple dmαKG addition^26^. This suggests that in certain contexts where Glud1 is incorporated into a complex, only the αKG locally supplied by Glud1 enables the activation of αKG-dependent factors within that complex, which may be one reason why simple αKG addition did not affect the effect of R162.

Moreover, our scMulti-omic analysis findings indicate that the expression of JAK-dependent genes under conditions of robust STAT activity is regulated by Glud1 catabolic activity (Fig. 4E–L and Supplementary Fig. 6O–R), suggesting that Glud1 modulates STAT function. Indeed, GLUD1 also interacts with the STAT5A^27^, a finding that was consistent with our results (Fig. 5G–J). Notably, inhibiting Glud1-mediated glutamate catabolism disrupted the Glud1–STAT5a interaction without affecting STAT5 phosphorylation levels (Fig. 5H–K), reinforcing the idea that Glud1 alters STAT5 function through physical association rather than by modulating its canonical activation. In addition to IKKβ, αKG activates the Ten-eleven translocation (TET), Jumonji domain-containing protein (JMJD), or Prolyl hydroxylase (PHD) families^28^. Therefore, if a STAT-based complex contains αKG-dependent factors, the association between Glud1 and the STAT family may be critical for regulating the functions of that complex. Although our findings showed that Glud1 inhibition maintained HSCs concurrently with suppressing the Glud1–STAT5a association (Fig. 5H–J), the precise molecular mechanisms by which this interaction regulates HSC fate remain to be elucidated through future multifaceted investigations.

Glud1 inhibition, coupled with robust JAK2 signaling, maintained HSCs during division, leading to HSC expansion (Fig. 5A–F). Conversely, some reports suggest that suppressing the JAK–STAT pathway may also maintain HSCs. Indeed, a lower dose of a JAK inhibitor improves the self-renewal capacity in human HSCs in vitro through optimizing the adaptation of HSCs into culture environments^29^. Moreover, unphosphorylated STAT5 contributes to HSC maintenance by upregulating transcriptional programs for quiescence and stemness^30^. These reports collectively suggest that the appropriate suppression of JAK–STAT signaling maintains HSCs. Given that STAT5 activation stimulates glutamine metabolism through Myc^31,32^, excessive activation of glutamine metabolism likely induces HSC differentiation through promoting Glud1-related reactions. Therefore, GLUD1 inhibition may be one of the mechanisms by which JAK–STAT suppression helps HSCs adapt to the culture environments. The resulting effect of robust JAK–STAT signaling coupled with Glud1 inhibition thus appears to represent an optimized balance that specifically contributes to HSC expansion.

HSCs during BM regeneration exhibited lower intracellular Glu levels even compared to those cultured under low Gln conditions (Fig. 2F), despite their robust divisional activity (Fig. 2B). This finding is in contrast with the in vitro result showing Glu-dependent proliferation (Fig. 2J). BM regeneration after transplantation still relies on Glu-dependent aminotransferase reactions^15^, suggesting that several factors within the BM support Glu-dependent aminotransferases in vivo even under low intracellular Glu. Given that Glu is utilized by both aminotransferases and Glud1, robust Glud1 inhibition may allow aminotransferases to preferentially utilize limited Glu. The enzymatic activity of Glud1 is allosterically suppressed by ATP, GTP, long-chain fatty acids, estrogens, and NADH^33^. Moreover, SIRT4 inhibits Glud1 activity through ADP-ribosylation^34^. Therefore, such negative regulators of Glud1 may contribute to self-renewing divisions during BM regeneration, potentially acting in conjunction with an unknown mechanism that decreases intracellular Glu levels in dividing HSCs.

In this study, we demonstrated that R162 treatment guided HSCs into self-renewal in vitro, enabling approximately 500-fold expansion of functional HSCs during 30-day culture. As illustrated in our finding summary (Supplementary Fig. 9), Glud1 inhibition under robust JAK2-STAT5 signaling suppresses the physical interaction between Glud1 and STAT5a. This altered interaction state coincides with the maintenance of HSC-specific chromatin accessibility at potential STAT5-binding regions and the preservation of the primitive transcriptional signature. Consequently, these multi-layered molecular features are closely associated with a blockade of the specific differentiation trajectory (Re-clusters 0 → 2 → 3 in Fig. 4), aligning with the active guiding of HSCs into self-renewal. However, some EPCR^High^ HSCs maintained in the presence of R162 produced differentiated progenitor cells during subsequent passage cultures, which suggests the existence of a differentiation pathway that circumvents Glud1 inhibition (such as the Re-clusters 0 → 1 → 4 trajectory in Fig. 4). Moreover, Glud1 inhibition was not effective in maintaining HSCs, showing suppressed cytokine signaling. Therefore, incorporating another culture system capable of maintaining such an HSC subset with lower JAK–STAT activity may further enhance overall HSC expansion when combined with our R162 system. To expand HSCs and develop ex vivo expansion of human HSCs for clinical applications, we will likely need not only optimization of our Glud1 inhibition system but also a strategy that accounts for such stem cell heterogeneity and various differentiation pathways more effectively.

## Methods

### Ethics

All animal experiments in this study were performed in strict accordance with the guidelines and relevant ethical regulations of Kumamoto University. The study protocol was formally reviewed and approved by the Institutional Animal Care and Use Committee of Kumamoto University (Approval/Protocol Number: A2023-058, A2025-076). Additionally, all recombinant DNA experiments were approved by the Gene Recombination Experiment Safety Committee of Kumamoto University (Approval Number: 6-061).

### Antibodies for flow cytometry

The following monoclonal antibodies were used in cell sorting and flow cytometric analyses to identify EPCR^High^ HSCs: BV421-conjugated anti-c-Kit (2B8), PE-conjugated anti-CD150 (TC15-12F12.2), APC/Cy7-conjugated anti-CD48 (HM48-1), APC-conjugated anti-EPCR (eBio1560, eBioscience, San Diego, CA), PE/Cy7-conjugated anti-Sca1 (E13-161.7), FITC or PerCP/Cy5.5-conjugated anti-B220/CD45R (RA3-6B2), FITC or PerCP/Cy5.5-conjugated anti-Mac-1 (M1/70), FITC or PerCP/Cy5.5-conjugated anti-Gr-1 (RB6-8C5), FITC or PerCP/Cy5.5-conjugated anti-CD3ε (145-2C11), and FITC or PerCP/Cy5.5-conjugated anti-TER119 (TER119).

The following monoclonal antibodies were used in flow cytometric analyses to analyze peripheral chimerism after the transplantation: FITC-conjugated anti-CD45.2 (104), PE-conjugated anti-CD45.1 (A20), BV421-conjugated anti-B220/CD45R (RA3-6B2), PE/Cy7-conjugated anti-Mac-1 (M1/70), PE/Cy7-conjugated anti-Gr-1 (RB6-8C5), APC-conjugated anti-CD4 (RM4-5) and APC-conjugated anti-CD8 (53-6.72). All antibodies were obtained from BioLegend (San Diego, CA).

To assess STAT5 phosphorylation in HSCs after 1-day culture, Alexa®488-conjugated anti-STAT5(pY694) (47/Stat5(pY694), BD Biosciences, Franklin Lakes, NJ) was used.

### Materials availability

No unique materials were generated during this study.

### Animal experiments

C57BL/6-Ly5.2 and C57BL/6-Ly5.1 mice (8–12 weeks of age) were obtained from Sankyo Lab Service Corporation (Tokyo, Japan) and Japan SLC Inc. (Shizuoka, Japan), respectively. All mice were housed in a specific-pathogen-free (SPF) facility with ad libitum access to food and water under controlled environmental conditions: a 12-h light/12-h dark cycle, an ambient temperature of 22 °C ± 2 °C, and a relative humidity of 50 ± 10%. To identify self-renewing HSCs during hematopoietic regeneration, mice were intravenously administered 250 mg/kg 5-FU (Kyowa Hakko Kirin, Tokyo, Japan).

For the isolation of primary donor HSCs and 5-FU administration, exclusively male C57BL/6-Ly5.2 mice were utilized to minimize physiological variability (e.g., hormonal fluctuations) and ensure a highly homogeneous stem cell source. For the transplantation assays, both male and female C57BL/6-Ly5.1 mice were used as recipients. Sex was not considered as an experimental variable in our study design and statistical analysis. Because our primary objective was to evaluate the fundamental, intrinsic stemness and repopulating capacity of the donor HSCs, the recipient mice served primarily as an in vivo engraftment system. Consequently, data from both male and female recipients were pooled for all analyses.

### Cell sorting and flow cytometric analyses

Briefly, to purify HSCs from whole BM cells, c-Kit^+^ cells were enriched by MicroBeads CD117 (Miltenyi Biotec Inc., Bergisch Gladbach, Germany; Cat# 130-091-224) and Auto MACS Neo separator (Miltenyi Biotec Inc.), following the manufacturer’s procedure. Freshly enriched cells or whole culture cells were stained with the indicated antibody cocktails (see Supplementary Table 1 for antibody details, including catalog numbers and dilutions) in PBS containing 2% FBS for 30 min on ice. Target cells were then sorted using MoFlo XDP or CytoFLEX SRT (Beckman Coulter, Brea, CA), after 1 μg/ml propidium iodide (PI) was added to select live cells. In addition, FACS Canto II (BD Biosciences), Moflo XDP, and CytoFLEX SRT were used for flow cytometric analyses. In the case of phosphorylated STAT5 (pY694), we used a PerFix EXPOSE Kit (Beckman Coulter) for cell fixation and permeabilization, following the manufacturer’s instructions. After the permeabilization, cells were stained with the indicated antibody (see Supplementary Table 1 for antibody details, including catalog numbers and dilutions) in PBS containing 2% FBS for 60 min on ice. Collected data (FCS files) were analyzed on Kaluza software v2.3 (Beckman Coulter). Representative gating strategies, including doublet and dead cell exclusion, are shown in Fig. S1A, D, 3, 4A, 7A.

### Long-term competitive repopulation assays

Sorted cells derived from C57BL/6-Ly5.2 were i.v. transplanted along with 2 × 10^5^ competitor cells derived from C57BL/6-Ly5.1 congenic mice into lethally irradiated (total of 10 Gy) C57BL/6-Ly5.2 mice. For 4, 12 and 20 weeks after transplantation, the chimerism and lineage distribution in donor cells were assessed in recipient mice. In the case of single cell transplantation, recipients showing more than 0.5% in each myeloid, B- and T-lymphoid lineages of chimerism were considered as multilineage-reconstituted mice.

### RNA sequencing

We prepared libraries for RNA-seq. Briefly, one hundred cells were directly sorted into a PCR tube filled with lysis buffer^35^ and subsequently the first strand of cDNA was synthesized by using PrimeScript RT reagent Kit (TAKARA Bio Inc., Kusatsu, Japan) and not-so random primers. To synthesize the 2nd strand, Klenow Fragment (3’−5’ exo-) (New England Biolabs Inc., Ipswich, MA) and complement chains of not-so-random primers were used. After double-stranded cDNA were purified using AMPure XP beads (Beckman Coulter), the RNA-Seq library was prepared and amplified using the Nextera XT DNA Sample Prep Kit (Illumina Inc., San Diego, CA), following the manufacturer’s procedure. Deep sequencing was conducted by the HiSeq 2000 system or NovaSeq X Plus (Illumina). Trimming, alignment (to mouse genome mm10), and read counts were performed by Trimmomatic v0.39, Hisat2 v2.2.1, and featureCounts v2.0.1, respectively. The normalization and comparison between groups were performed using DESeq2 v 1.46.0. Using these transcriptome data, we performed a Principal Component Analysis (PCA) and a Hierarchical Cluster Analysis (HCA) using DESeq2 v 1.46.0 and pheatmap v1.0.13 in R-base v4.4.2, respectively. Moreover, gene set enrichment analysis (GSEA) was performed using GSEA v4.3.2 software (http://www.broad.mit.edu/gsea). All gene sets used in the GSEA were obtained from the Broad Institute database unless otherwise stated. The raw and processed RNA-seq datasets have been deposited in the Gene Expression Omnibus (GEO) under accession numbers [GSE279409] and [GSE338821] (see Data Availability).

### Omni-ATAC-seq

Omni-ATAC-seq was performed with minor modifications^7,36^. In brief, sorted HSCs were lysed with ATAC-resuspension buffer containing 1% digitonin^36^ and subsequently centrifuged at 500 × g for 10 min at 4 °C. The cell pellet was washed with ATAC-resuspension buffer to exclude mitochondria. The resulting nuclear pellets were subjected to a transposition reaction using Tagment DNA Enzyme TDE1 (Illumina Inc.). Following PCR amplification using barcoded primers (see Supplementary Table 2 for primer sequences^37^), the ATAC-seq libraries were purified using AMPure XP beads (Beckman Coulter). Deep sequencing was performed on HiSeq 2000 system (Illumina) with 150-bp paired-end reads. For data analysis, adapter trimming, alignment (to mouse genome mm10), peak calling, and quantification were performed using Trimmomatic v0.39, Bowtie2 v2.4.2, MACS2 v2.2.7.1, and HOMER v4.11, respectively. Heatmaps were prepared using deepTools v3.5.1. The newly generated ATAC-seq datasets have been deposited in the GEO database under accession number [GSE279407] (see Data Availability). The ATAC-seq data of EPCR^High^ and EPCR^Low^ HSCs in the steady state were downloaded from GSE187014 and reanalyzed^7^.

### Measurement of intracellular amino acid level

Glu levels were assessed using the Glutamine/Glutamate-Glo™ Assay (Promega, Madison, WI) according to the manufacturer’s protocol. To assess Asp, Ala, and Ser levels, the Nexera X2 high-performance liquid chromatography system (Shimadzu, Kyoto, Japan) was used, after 2 × 10^4^ sorted cells were denatured by 0.1 N HCl and o-phthaldialdehyde, 3-mercaptopropionic acid, and boric acid were added to the cell lysate to fluorescently label free amino acids.

### EdC uptake assay

First, 150 mg/kg of EdC (Tokyo Chemical Industry, Tokyo, Japan) was i.p. administered. After 24 h, whole BM cells were stained with the indicated antibody cocktails (see Supplementary Table 1 for antibody details, including catalog numbers and dilutions) in PBS containing 2% FBS for 30 min on ice. Subsequently, EdC uptake in indicated cells was assessed using a Click-iT Plus EdU Alexa Fluor 488 Flow Cytometry Assay kit (Thermo Fisher Scientific, Waltham, MA), following the manufacturer’s instructions.

### HSC cultures

HSCs were sorted and cultured in RPMI-based medium (Supplementary Table 3; Gmep, Kurume, Japan) supplemented with 10 ng/ml mouse SCF (R&D Systems, Minneapolis, MN), 100 ng/ml mouse TPO (R&D Systems), and 2% AlbuMAX™ I Lipid-Rich BSA (Thermo Fisher Scientific), 1× penicillin streptomycin (Thermo Fisher Scientific), 1× Insulin-Transferrin-Selenium-Ethanolamine (ITS-X) (Thermo Fisher Scientific) and 10 mM HEPES (Thermo Fisher Scientific) in a 96-well U-bottom plate. To prepare low Gln conditions, the original Gln concentration (2 mM) was reduced to 0.02 mM. Specific factors were pharmacologically inhibited using the following reagents: 250 μM aminooxyacetic acid (AOA, an aminotransferase inhibitor; Sigma-Aldrich; 200× stock in H_2_O), 30 μM R162 (a Glud1 inhibitor; Merck; 1,000× stock in DMSO), 1.5 μM ruxolitinib (a JAK1/2 inhibitor; Selleck; 1,000× stock in DMSO), 1.5 μM fedratinib (a JAK2 inhibitor; Selleck; 1,000× stock in DMSO), and 4 μM SCH772984 (an Erk1/2 inhibitor; Selleck; 2,500× stock in DMSO). To examine the effect of Glud1-related metabolites, 2 mM dmαKG (Sigma-Aldrich; 6.37 M liquid stock) or 3 mM nicotinamide riboside (NR; Selleck; 100 mM stock in H_2_O) were added to the medium. For all experiments involving DMSO-solubilized compounds, an equivalent concentration of DMSO was used as a vehicle control. The F12-PVA-based culture system was employed based on a protocol originally developed by Wilkinson et al^4^. The medium consisted of Ham’s F-12 medium (Thermo Fisher Scientific) supplemented with 10 ng/ml mouse SCF (R&D Systems, Minneapolis, MN), 100 ng/ml mouse TPO (R&D Systems), and 0.1% PVA (Sigma-Aldrich, Cat# P8136), 1× Penicillin-Streptomycin-Glutamine (Thermo Fisher Scientific), 1× Insulin-Transferrin-Selenium-Ethanolamine (ITS-X) (Thermo Fisher Scientific) and 10 mM HEPES (Thermo Fisher Scientific).

### *Glud1* knockout (KO) using CRISPR/Cas9

Gene editing of HSCs was performed using CRISPR/Cas9 system as previously described^38^ with minor modifications. Briefly, L^-^ESLAM HSCs were sorted and subjected to a 1-day pre-culture under the normal culture condition described above. The sgRNA/Cas9 complex was assembled by mixing 1 µg of the Cas9 protein (Thermo Fisher Scientific) with 1 µg of a mixture of three sgRNAs targeting *Glud1* (5’-GCAGCAGCGCTTCGCCCAGA-3’, 5’-GCAGACTCAGCCGCGCTGCT-3’, 5’-CGGCGTGAGCCCGGGTTGCG-3’; Synthego, Redwood City, CA) or control sgRNA (Thermo Fisher Scientific). The mixture was incubated for 30 min on ice. Finally, the pre-cultured cells were then electroporated with the complex using a single pulse at 1700 V and 20 ms.

To validate KO efficiency, the genome was extracted from the cells 24 h after the electroporation. The region including the target sites of the three sgRNAs was amplified using the primer pair (Forward: 5’-AGCTCCGGTCTTTACAGCTCC-3’; Reverse: 5’-CGAAGAAGCCCTCCACCATCTTGAA-3’). These PCR products were then sequenced by NextSeq 1000 system (Illumina). Knockout efficiency was calculated by analyzing the polymorphism of sequenced products using CRISPResso2 v2.2.14. To examine the effect of *Glud1* KO on HSCs, EPCR^High^ HSCs (EPCR^High^ L^-^ESLAM cells) were sorted and cultured under normal conditions for 5 days.

### NAD^+^/NADH measurement

Following the manufacturer’s protocol, 10,000 sorted HSCs were used to assess the ratio of NAD^+^ to NADH by using NAD/NADH-Glo™ Assays (Promega).

### αKG measurement

Following the manufacturer’s protocol, 150,000 sorted HSCs were used to assess the level of αKG by using α-Ketoglutarate Assay Kit (DOJINDO LABORATORIES, Kumamoto, Japan).

### Single-cell multi-ome analysis

Nuclei were isolated from whole cells obtained after a 5-day culture of EPCR^High^ HSCs in the absence or presence of R162 (30 µM), following the Nuclei Isolation for Single Cell Multiome ATAC + Gene Expression Sequencing protocol (10x Genomics, Pleasanton, CA), and then resuspended in a cell concentration of 5000 cells/µl. Cells were processed using a Chromium Next GEM Chip J Single Cell Kit (10xGenomics, PN-1000230), Chromium Next GEM Single Cell Multiome Reagent Kit A (10x Genomics, PN-1000284), and Chromium Next GEM Single Cell Multiome ATAC Kit A (10x Genomics, PN-1000281), following the manufacturer’s User Guide. Targeted nuclei recovery was around 9000. The single-cell RNA and ATAC sequencing libraries were prepared using Dual Index Kit TT Set A (10x Genomics, 1000215) and Single Index Kit N Set A (10x Genomics, 1000212), respectively. The resulting libraries were sequenced by PE50 sequencing using a DNBSEQ-G400 system (MGI Tech, Shenzhen, China). The raw and processed scMultiome datasets have been deposited in GEO under accession number [GSE279490] (see Data Availability). Sequenced data were processed using CellRanger ARC software v2.0.2, with alignment to the mouse (mm10) genome. Clustering and gene expression analyses were performed using the combination of Seurat v5.4.0 and Signac v1.16.0 packages on R-base v4.4.2^39,40^. The gene sets used for module score calculations—including MolO, NoMO^20^, vWF-HSCs^22^, HSCs, MPP1–5^21^, PreMegEs^23^, and MkPs^22^—are listed in Supplementary Data 1. Monocle 3 v1.4.26 and CytoTRACE2 v1.1.0 were employed for pseudotime analysis and root cell identification, respectively^41,42^. RNA velocity analysis and Transcription factor motif analysis was conducted using scVelo v0.3.3 and chromVAR v1.28.0 based on the JASPAR database (JASPAR2020 v0.99.10), respectively^43,44^. JAK-dependent genes were identified as those significantly down-regulated following Ruxolitinib treatment (fold-increase >2 and p < 0.05)^30^. Correlation coefficients between gene expression levels and chromatin accessibility were calculated by R v4.4.2. To identify potential STAT5-binding peaks, overlapping regions between all detected scATAC-seq peaks from our multiome analysis and STAT5a/b-binding sites in blood cells (retrieved from ChIP-atlas (https://chip-atlas.org/); Supplementary Data 2) were extracted by using bedtools v2.30.0.

### Immunoprecipitation (IP) and Western blotting (WB)

Whole cell lysate was prepared from 2 × 10^7^ A-6 cells (RIKEN BRC, RCB1517) using Modified RIPA buffer (50 mM Tris-HCl, pH 7.5, 150 mM NaCl, 1% NP-40, 0.5% sodium deoxycholate, 1 mM EDTA, 10% glycerol) supplemented with protease inhibitor (Thermo fisher, 87786). Subsequently, the lysate was treated with MgCl₂ (2 mM final) and Benzonase (25 U/ml, Millipore) for nuclease treatment. For IP, Pierce^TM^ Crosslink Magnetic IP/Co-IP Kit (Thermo Fisher Scientific) were incubated with 10 µg of anti-STAT5 antibody (Santa Cruz Biotechnology, Dallas, TX, A-9, Cat # sc-74442) to prepare antibody-captured beads, following the manufacturer’s procedure. The mouse isotype control antibody (Cell Signaling Technology, G3A1, Cat # 5415S) serves as control. The A-6 cell lysates were then mixed with the antibody-captured beads and incubated for 2 h at 4 °C with rotation. The beads were subsequently washed twice, and immunoprecipitated proteins were eluted with LDS sample buffer (Thermo Fisher Scientific) at 95 °C for 10 min to elute. The resulting IP samples, along with an input control, were analyzed by SDS-PAGE and WB using a primary antibody against Glud1 (1/1000 dilution; Cell Signaling Technology, D9F7P, Cat# 12793 T) and HRP-conjugated anti-secondary rabbit IgG antibody (1/2000 dilution; Cell Signaling Technology, Lot#34, Cat# 7074P2). To detect immunoreactive bands, SuperSignal West Femto Maximum Sensitivity Substrate (Thermo Fisher Scientific) was used.

### Proximity ligation assay

According to the manufacturer’s procedure, we employed Duolink® In Situ Proximity Ligation Assay Kit (Sigma-Aldrich) to examine the association between Glud1 and STAT5a in HSCs. Briefly, HSCs were fixed with 2% paraformaldehyde and permeabilized with 0.1% Triton X-100 in PBS. Permeabilized cells were incubated with Duolink® blocking solution at 37 °C for 1 h. Cells were treated with primary rabbit or goat antibodies against STAT5a (1/200 dilution; Cell Signaling Technology, D2O6Y, Cat# 94205S) or GLUD1/2, (1/200 dilution; Thermo Fisher Scientific, Lot# 79522760, Cat# PA5-19267) for overnight at room temperature. Negative control samples were stained with rabbit IgG isotype control (1/200 dilution; Cell Signaling Technology, DA1E, Cat# 3900S) and goat IgG isotype control (1/200, Thermo Fisher Scientific, Lot#ZA-385455, Cat# 02-6202). Cells were washed, then stained with PLUS-oligonucleotide-conjugated anti-Rabbit IgG antibody (1/5 dilution; Sigma-Aldrich, Lot# SLCC5674, Cat# DUO82002-100RXN) and MINUS-oligonucleotide-conjugated anti-Goat IgG antibody (1/5 dilution; Sigma-Aldrich, Lot# SLCR2992, Cat# DUO82006-100RXN) for 1 h at 37 °C. After washing, samples were treated with Duolink® Ligation solution for 1 h at 37 °C to form rolling circle DNA. The circle DNA was subsequently amplified using Duolink® Amplification solution for 10 h at 37 °C. Cells were washed, stained with Detection solution for 30 min at 37 °C, and counterstained with DAPI to visualize nuclei. Coverslips were then mounted using ProLong™ Diamond Antifade Mountant (Thermo Fisher Scientific). PLA signals and nuclei were assessed using a confocal fluorescence microscope system (FV1200; Olympus, Tokyo, Japan). Fiji software v1.54p (NIH) was used to process confocal images and quantify the number of foci per cell. The frequency of HSCs having foci was calculated using more than 20 cells.

### Statistical analysis

Unless otherwise indicated, data are presented as means, and error bars indicate ± standard deviation (S.D.) from more than three independent biological replicates obtained from more than two independent experiments. In each experiment, the statistical tests used are indicated in the figure legends. Briefly, in the case of parametric comparisons between two groups and more than two groups, statistical analysis was performed using an unpaired two-sided *t-*test and one-way analysis of variance (ANOVA) with Tukey’s multiple comparison test, respectively. Significance was assumed for *p < 0.01 for **p < 0.05, and N.S. denotes not significant. Analyses were performed using GraphPad Prism v11.0.0 (GraphPad Software, Inc., San Diego, CA). The enrichment of genes exhibiting high correlation (*r* > 0.5) between gene expression levels and chromatin accessibility was assessed by using the hypergeometric test in R v4.4.2.

### Reporting summary

Further information on research design is available in the Nature Portfolio Reporting Summary linked to this article.

## Supplementary information

Supplementary Information

Description of Additional Supplementary Files

Supplementary Data 1

Supplementary Data 2

Reporting Summary

Transparent Peer Review file

## Source data

Source Data

## Data Availability

All reported data and additional information required to reanalyze the data reported in this paper will be shared by the lead contact upon reasonable request. RNA-seq, ATAC-seq, and scMultiome-seq data are accessible in the GEO under accession number GSE279407, GSE279409, GSE338821 and GSE279490. Source data are provided with this paper.
