# Supplementary material for: Suppressing glutamate dehydrogenase guides hematopoietic stem cell divisions into self-renewal
Source: Nat Commun. 2026 Sep 5;17:10558. doi: 10.1038/s41467-026-77151-6 (PMC13642937; doi:10.1038/s41467-026-77151-6)
Supplement: Supplementary file 1 [file 41467_2026_77151_MOESM1_ESM.pdf]

SUPPLEMENTARY INFORMATION

Suppressing glutamate dehydrogenase guides hematopoietic stem cell divisions into self-renewal

Michihiro Hashimoto[1], Michie Uchikawa[1], Yuichiro Arima[2], Akiyasu Iwase[3], Ayano Yahagi[4], Mizuki Ishimaru[1], Tomomasa Yokomizo[4], Ayako Nakamura-Ishizu[4], Yosuke Tanaka[5], Kenichi Miharada[6], Hiroki Kurihara[3,7], Goro Sashida[8], Toshio Suda[5,9]* and Terumasa Umemoto[1,10]*

1) Laboratory of Hematopoietic Stem Cell Engineering, International Research Center for Medical Sciences, Kumamoto University, Kumamoto, Japan
2) Department of Anatomy, Faculty of Life Sciences, Kumamoto University, Kumamoto, Japan
3) Isotope Science Center, The University of Tokyo, Tokyo, Japan
4) Department of Microscopic Anatomy and Developmental Biology, Tokyo Women's Medical University, Tokyo, Japan
5) Laboratory of Stem Cell Regulation, International Research Center for Medical Sciences, Kumamoto University, Kumamoto, Japan
6) Laboratory of Proteostasis in Stem Cell, International Research Center for Medical Sciences, Kumamoto University, Kumamoto, Japan
7) Laboratory of Multicellular dynamics, International Research Center for Medical Sciences, Kumamoto University, Kumamoto, Japan
8) Laboratory of Transcriptional Regulation in Leukemogenesis, International Research Center for Medical Sciences, Kumamoto University, Kumamoto, Japan
9) Institute of Hematology, Blood Diseases Hospital, Chinese Academy of Medical Sciences & Peking Union Medical College, Tianjin, China
10) Research & Investigation Department, Project Division, the Chemo-Sero-Therapeutic Research Institute (KAKETSUKEN), Kumamoto, Japan

Correspondence and requests for materials should be addressed to T.U. (email: umemoto@kumamoto-u.ac.jp) and T.S. (email: sudato@keio.jp)

Supplementary Figure 1

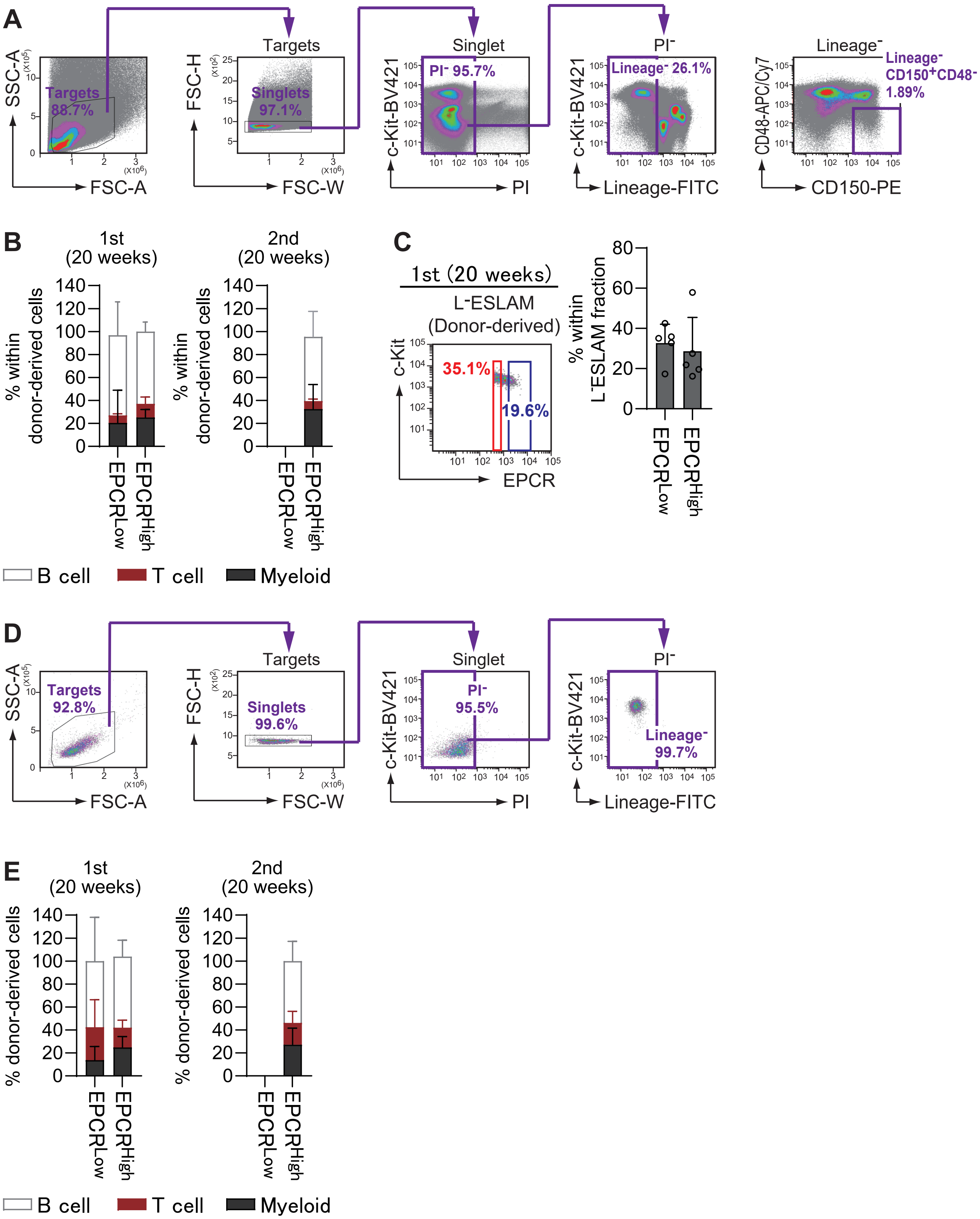

Supplementary Figure 1. The comparison between EPCR^{High} and EPCR^{Low} cells within L$^-$ESLAM fractions.

(A) Gating strategy for Lineage$^-$CD150$^+$CD48$^-$ cells, including sequential steps for doublet and propidium iodide (PI)$^+$ exclusion, used in Figure 1A to identify EPCR^{High} or EPCR^{Low} cells within the Lineage$^-$EPCR$^+$CD150$^+$CD48$^-$ (L$^-$ESLAM) HSC fraction. Numbers in dot plots represent the percentage of indicated gate.

(B) Lineage distribution of reconstituted hematopoietic cells within donor-derived cells 20 weeks after primary (1st) or secondary (2nd) transplantation of freshly isolated EPCR^{High} or EPCR^{Low} L$^-$ESLAM cells (Related to Figure 1C). Data are means ± S.D. (n = 5 biological replicates, two independent experiments, no significant difference by the two-tailed unpaired *t*-test).

(C) Frequency of EPCR^{High} or EPCR^{Low} cells within the bone marrow (BM) L$^-$ESLAM fraction after primary transplantation of EPCR^{High} HSCs. Data are shown as means ± S.D. (n = 5 biological replicates, two independent experiments, no significant difference via the two-tailed unpaired *t*-test).

(D) Representative gating strategy, incorporating doublet and PI$^+$ exclusion, for Lineage$^-$EPCR$^+$ cells used in Figures 1E, 2J, 3C, 5A, 6B, 7B, Supplementary Figures 4F, 5D, 7C, and 8B to identify EPCR^{High} or EPCR^{Low} cells within the L-ESLAM HSC fraction. Numbers in dot plots represent the percentage of indicated gate.

(E) Lineage distribution of reconstituted hematopoietic cells within donor-derived cells 20 weeks after 1st or 2nd transplantation. Donor cells were EPCR^{High} or EPCR^{Low} L$^-$ESLAM cells obtained after 4-day culture of EPCR^{High} L$^-$ESLAM cells (Related to Figure 1J). Data are means ± S.D. (n = 6 biological replicates, two independent experiments, no significant difference by the two-tailed unpaired *t*-test).

Supplementary Figure 2

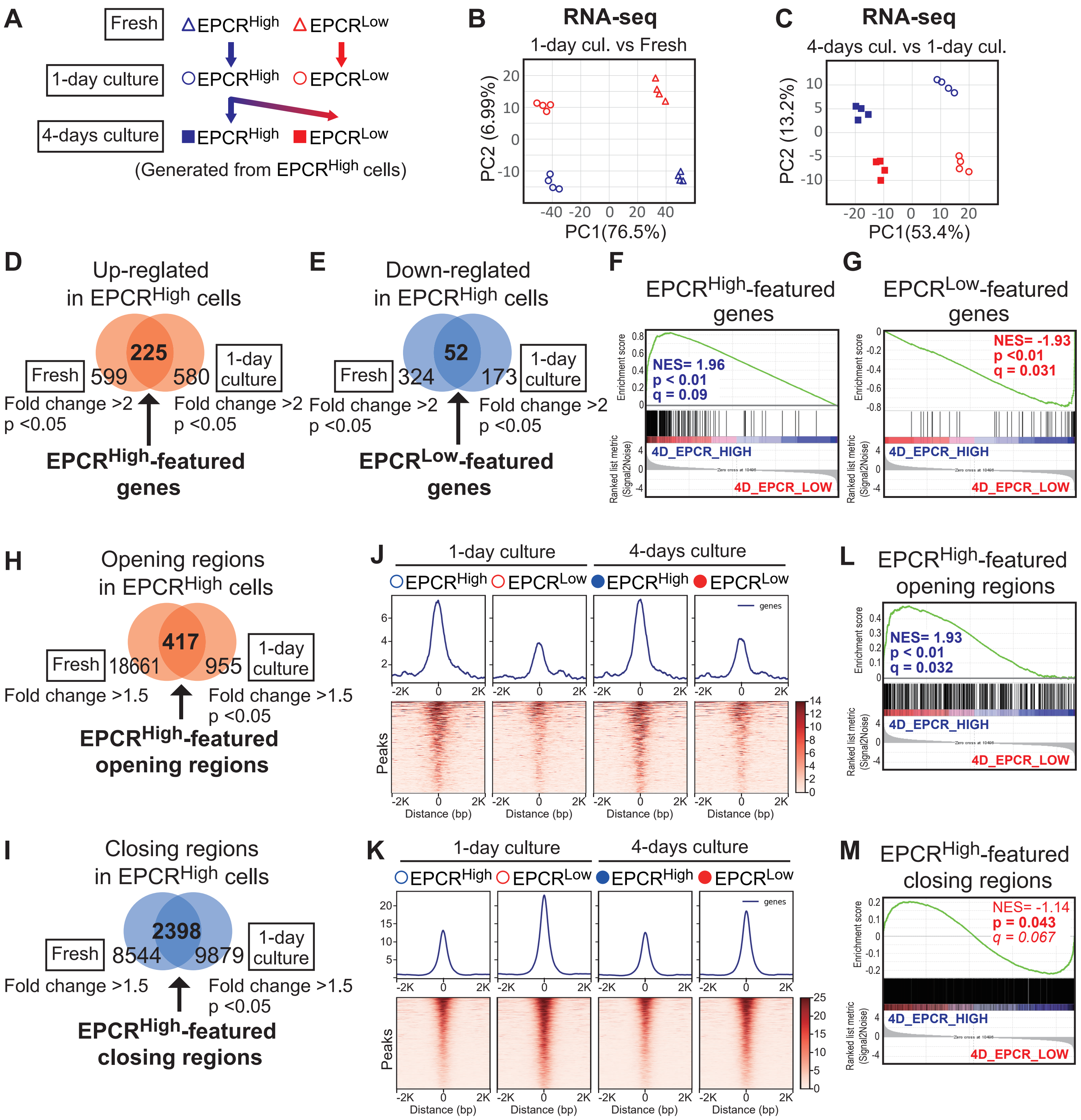

Supplementary Figure 2. Gene expression and chromatin accessibility in EPCR^{High} and EPCR^{Low} cells within L^{-}ESLAM fractions.

(A) Sample information for EPCR^{High} or EPCR^{Low} L^{-}ESLAM cells in the steady state, after a 1-day culture or after a 4-day culture in RPMI-based medium.

(B, C) Principal Component Analysis (PCA) plots based on RNA-seq for EPCR^{High} or EPCR^{Low} L^{-}ESLAM cells comparing steady state vs. 1-day culture (B) and 1-day vs. 4-day culture (C).

(D, E) Extraction of EPCR^{High}- featured (D, upregulated) or EPCR^{Low}- featured (E, downregulated) genes. Featured genes represent shared candidates between steady state and 1-day culture conditions.

(F, G) Enrichment of EPCR^{High}-featured (F) or EPCR^{Low}-featured (G) genes comparing 4-day cultured EPCR^{High} vs. EPCR^{Low} cells within the L^{-}ESLAM HSC fraction. NES and q values represent normalized enrichment scores and FDR, respectively.

(H, I) Extraction of EPCR^{High}-featured opening (H, more accessible) or closing (I, more closed) regions. Featured regions represent shared candidates between steady state and 1-day culture conditions

(J, K) Average plot (top) and heatmap (bottom) of EPCR^{High}-featured opening or closing regions in indicated samples.

(L, M) Enrichment of genes related to EPCR^{High}-featured opening (L) or closing (M) regions comparing 4-day cultured EPCR^{High} vs. EPCR^{Low} cells within the L^{-}ESLAM fraction.

Supplementary Figure 3

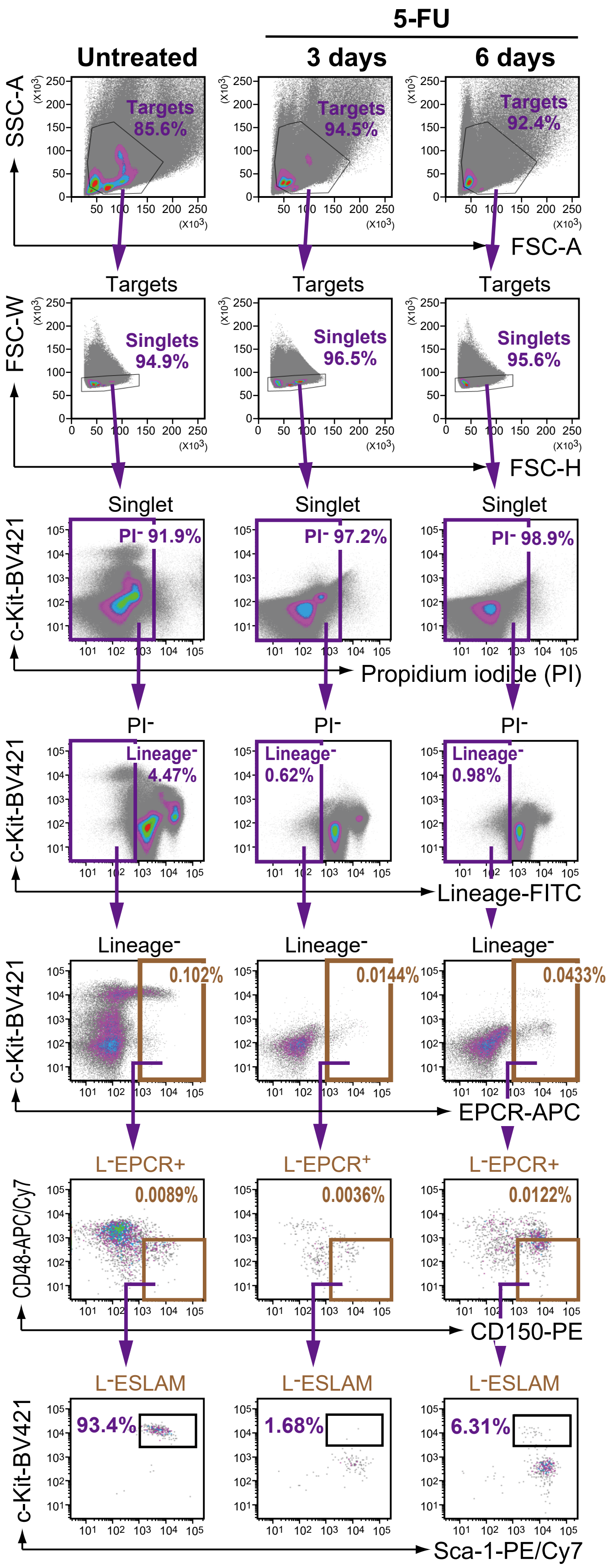

105

Supplementary Figure 3. The gating strategy for HSCs in the steady state during BM regeneration.

The representative gating strategy, incorporating doublet and PI^+ exclusion, to analyze HSCs (L^-ESLAM HSCs) in the steady state or after 5-FU administration (250 mg/kg, i.v.). These doublet and PI^+ exclusion to extract $Lineage^-$ cells were also used in Figure 2A. Numbers written by purple or brown color in dot plots represent the percentage of indicated gate or within whole BM cells, respectively.

Supplementary Figure 4

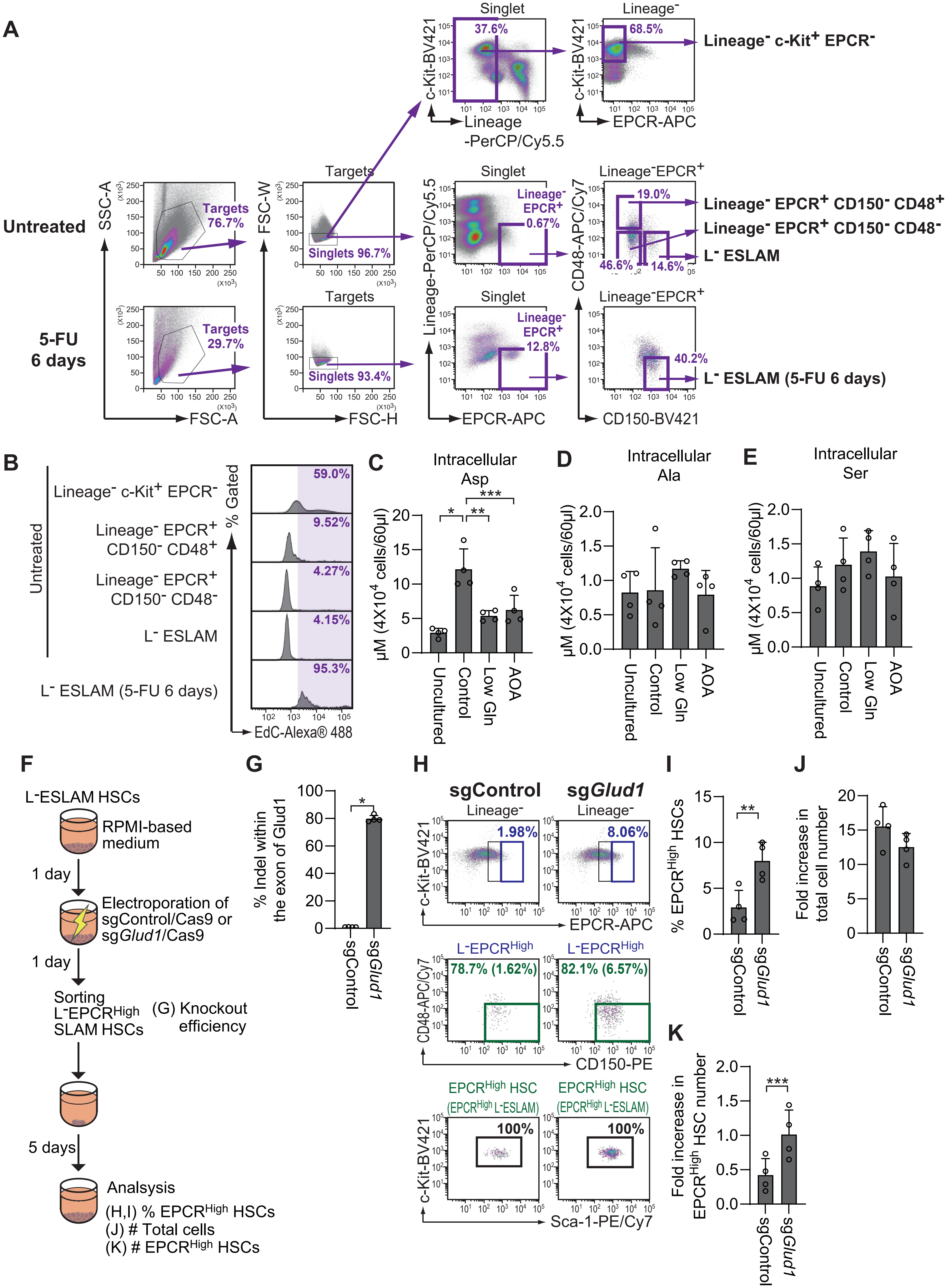

Supplementary Figure 4. The effect of suppressing Glu catabolism on HSC maintenance during *ex vivo* culture.

(A) The representative gating strategy, incorporating doublet exclusion, to define indicated fractions in the steady state or after 5-FU administration (250 mg/kg, i.v.). Numbers in dot plots represent the percentage of indicated gate.

(B) The representative histogram to analyze EdC uptake in indicated fractions in the steady state or after 5-FU administration. Purple area and numbers in histograms represent the gate defining EdC^+ cell and the percentage of EdC^+ cells, respectively.

(C-E) Intracellular levels of Aspartate (Asp) (C), Alanine (Ala) (D), and Serine (Ser) (E) in HSCs after a 1-day culture under the indicated conditions in RPMI-based medium. Data are shown as means ± S.D. (n = 4 biological replicates, two independent experiments; **p* <0.0001, ***p* = 0.0014, ****p* = 0.004 using one-way ANOVA with Tukey's test).

(F-K) Strategy to examine the effect of *Glud1* knockout (KO) using Crispr/Cas9 on HSCs during *ex vivo* culture in RPMI-based medium (F). *Glud1* KO efficiency 1 day after electroporation of the sgRNA/Cas9 complex (G). The frequency of $EPCR^{High}$ L^- ESLAM cells (H, I) and total cell number (J), and $EPCR^{High}$ HSC number (K) after a 5-day culture of electroporated $EPCR^{High}$ HSCs. Numbers or numbers within parentheses in the dot plots represent the percentage within the indicated gate or whole culture cells, respectively. Data are shown as means ± S.D. (n = 4 biological replicates, two independent experiments; **p* <0.0001, ***p* = 0.011, ****p* = 0.039 using the two-tailed unpaired *t*-test).

Supplementary Figure 5

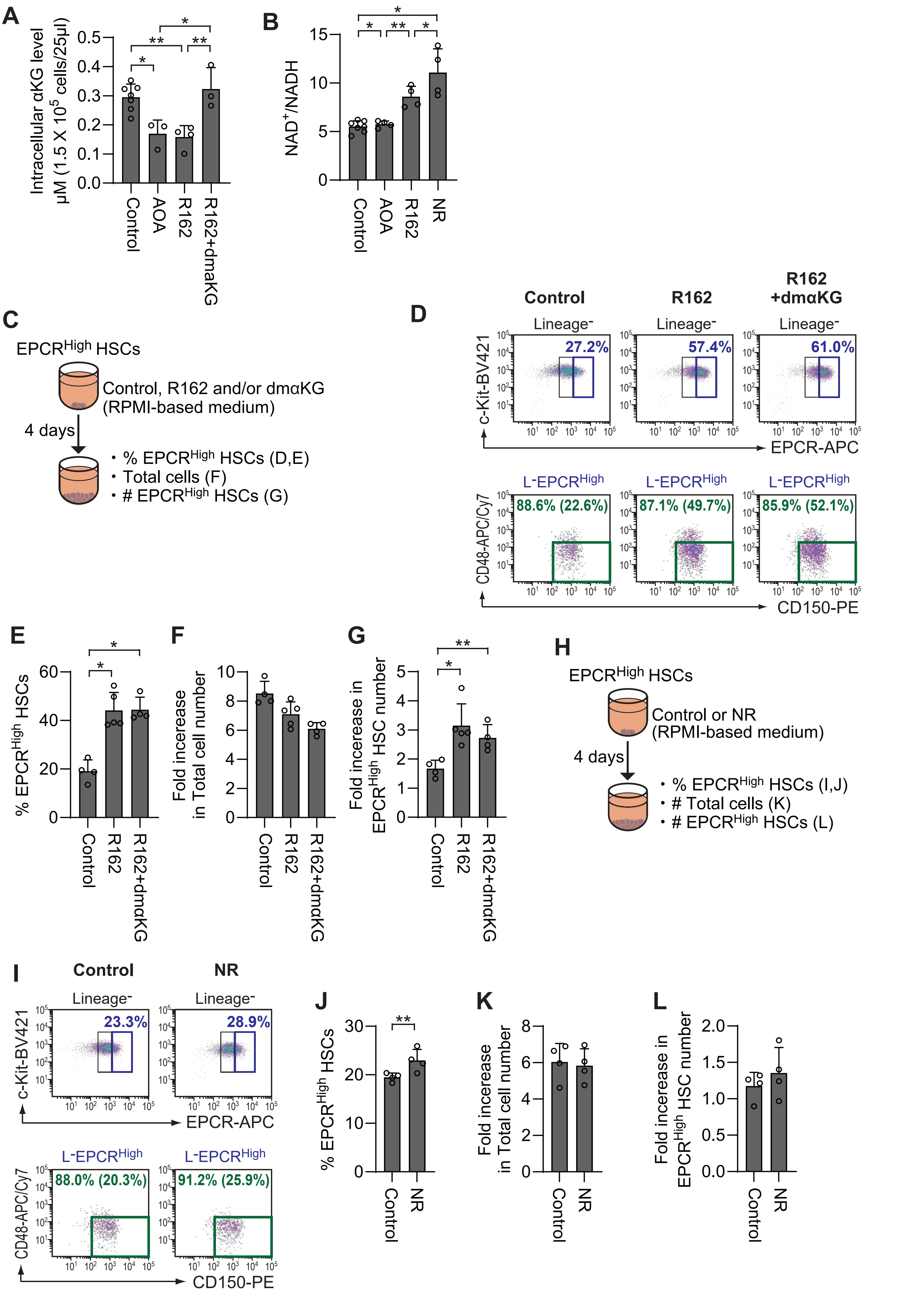

Supplementary Figure 5. The effect of modifying Glu-related metabolism on HSC maintenance during *ex vivo* culture.

(A, B) The effects of R162 on αKG (A) and NAD^+/NADH ratio (B) in HSCs after a 1-day culture in RPMI-based medium. Data are shown as means ± S.D. (n = 7 biological replicates for Control from four independent experiments; n = 3 for AOA, n = 4 for R162 and n =3 for R162 + dmαKG from two independent experiments, *p < 0.01 using one-way ANOVA with Tukey's test; Exact *P* values in the Source Data).

(C-G) The effects of dimethyl-αKG (dmαKG), a cell membrane-permeable αKG precursor, during a culture of $EPCR^{High}$ HSCs in RPMI-based medium for 4 days in the presence of R162 (a Glud1 inhibitor) (C). The frequency of $EPCR^{High}$ L^-ESLAM HSCs (D and E), total cell number (F), and $EPCR^{High}$ HSC number (G). Numbers or numbers within parentheses in the dot plots represent the percentage within the indicated gate or whole culture cells, respectively. Data are shown as means ± S.D. (n = 4 biological replicates for Control, n = 5 for R162 and n =4 for R162 + dmαKG, two independent experiments, *$p < 0.01$ using one-way ANOVA with Tukey's test; Exact *P* values in the Source Data).

(H-L) The effects of NAD^+ precursors (Nicotinamide Riboside (NR)) on HSCs during a 4-day culture in RPMI-based medium (H). $EPCR^{High}$ L^-ESLAM HSC frequency (I and J), total cell number (K), and $EPCR^{High}$ HSC number (L) after a 4-day culture of $EPCR^{High}$ HSCs in the presence of NR. Data are shown as means ± S.D. (n = 4 biological replicates, two independent experiments, **$p < 0.05$ using the two-tailed unpaired *t*-test).

Supplementary Figure 6-1

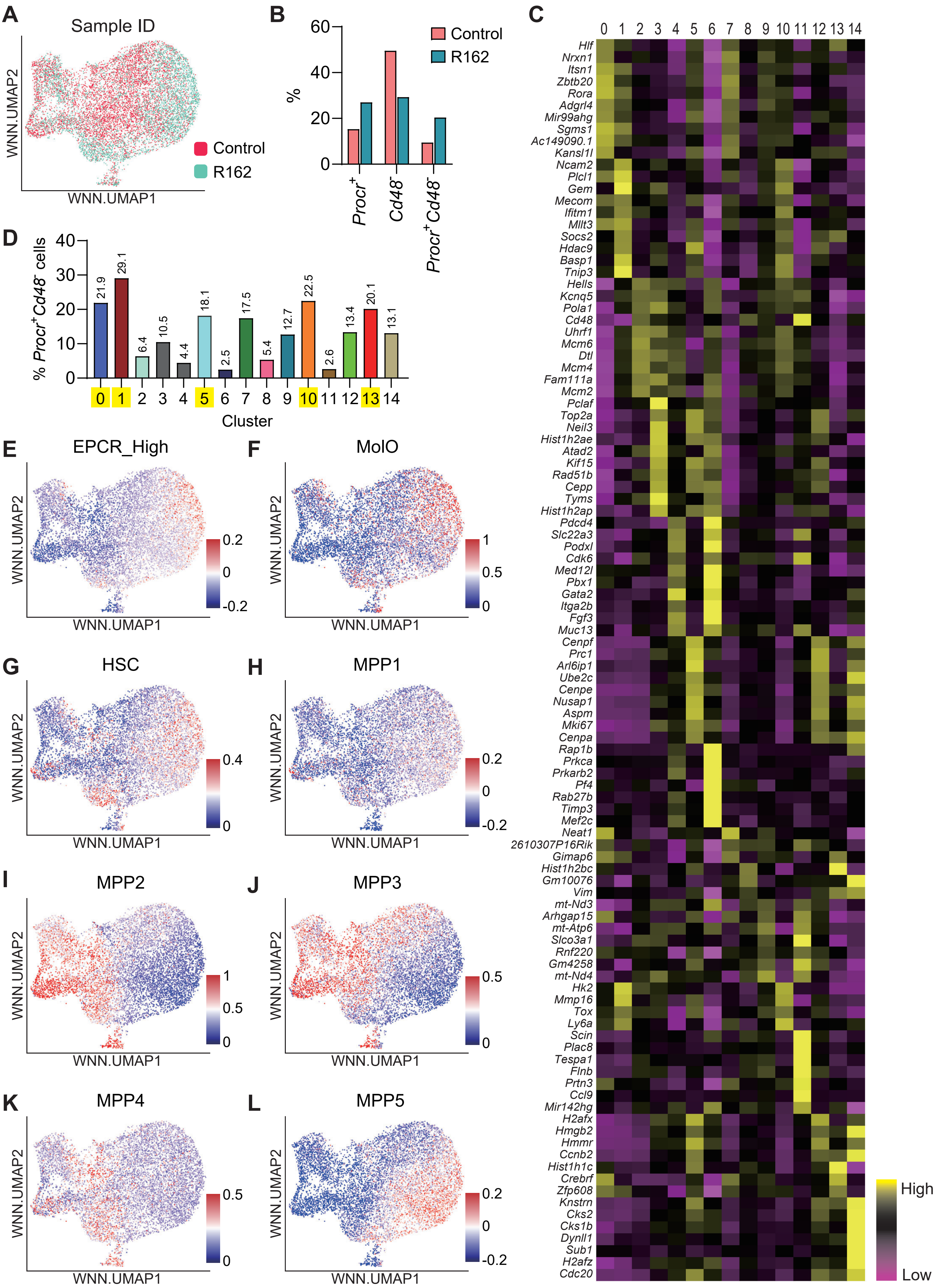

Supplementary Figure 6-2

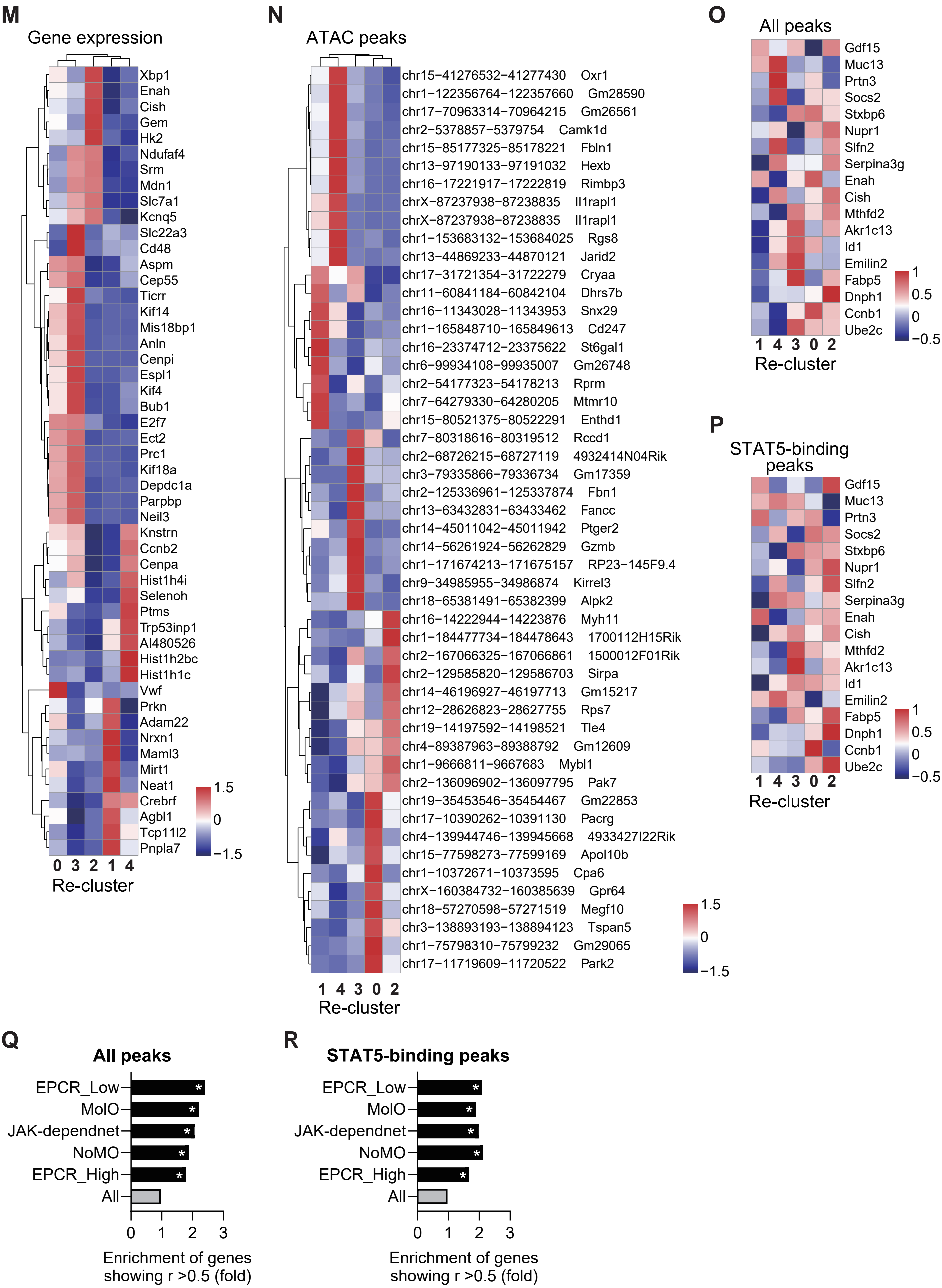

Supplementary Figure 6. Featured genes in each cluster sorted based on gene expression patterns.

(A) Feature plot for cells cultured in the absence (Control) or presence of R162.

(B) Frequencies of *Procr*$^+$, *Cd48*$^-$ and *Procr*$^+$*Cd48*$^-$ cells after the culture in the absence or presence of R162.

(C) The heatmap representing top 10 differentially expressed genes (DEGs) for each cluster.

(D) The frequency of *Procr*$^+$*Cd48*$^-$ cells within each cluster after the culture. Highlighted bars indicate the top five clusters with the highest *Procr*$^+$*Cd48*$^-$ cell frequency.

(E-L) Module scores of EPCR High feature (E), MolO (F), HSC (G), MPP1–5 (H–L) [1, 2].

(M) Heatmap representing top 10 of DEGs in each re-cluster.

(N) Heatmap representing top 10 of most accessible regions in each re-cluster.

(O) Heatmap representing chromatin accessibility at JAK-dependent gene loci across the re-clusters.

(P) Heatmap representing the accessibility of potential STAT5-binding peaks within JAK-dependent gene loci for each re-cluster.

(Q and R) Enrichment of genes exhibiting a high correlation ($r > 0.5$) between gene expression and chromatin accessibility, calculated across all detected peaks (Q) and specifically at potential STAT5-binding peaks (R). Asterisks indicate statistically significant enrichment determined by the hypergeometric test.

Supplementary Figure 7

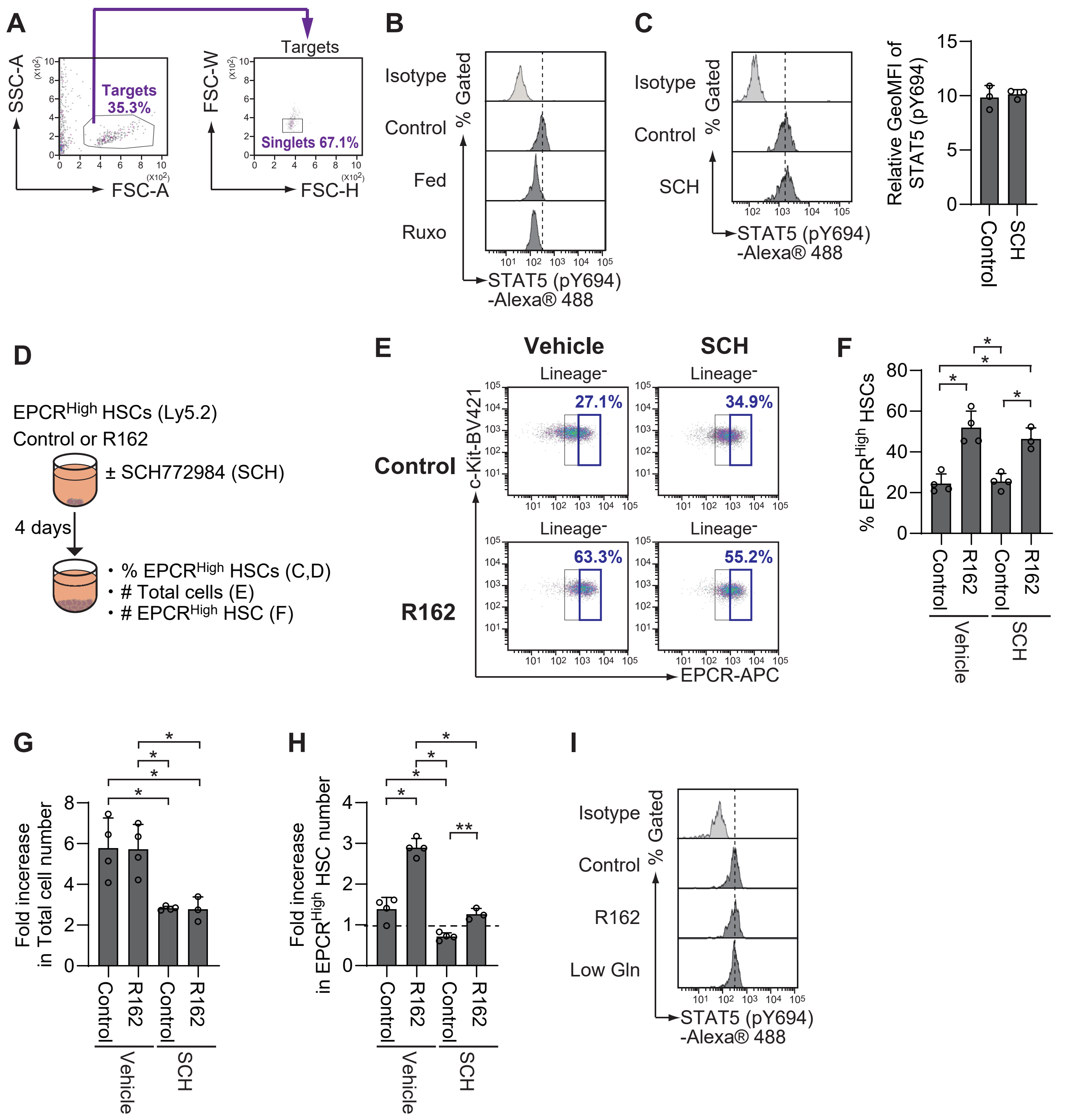

Supplementary Figure 7. The effect of Erk1/2 inhibition on R162-dependent HSC maintenance

(A) Representative gating strategy, incorporating doublet exclusion, to analyze phosphorylated STAT5 (pY694) used in Supplementary Figures S7B, C and I. Numbers in dot plots represent the percentage of indicated gate.

(B) The representative histogram showing phosphorylated STAT5 (pY694) level in HSCs after a 1-day culture in the absence or presence of fedratinib (Fed) or Ruxolitinib (Ruxo) (related to Figure 5A).

(C) Relative geometric means of the fluorescence intensity (GeoMFI) for phosphorylated STAT5 (pY694) in HSCs after a 1-day culture in the absence or presence of SCH772984 (SCH), an Erk1/2 inhibitor. Data are shown as means ± S.D. (n = 3 biological replicates, two independent experiments, no significant difference by the two-tailed unpaired *t*-test).

(D) Strategy to examine the impact of an Erk1/2 inhibitor on the positive effect of R162 (a Glud1 inhibitor) on the maintenance or expansion of $EPCR^{High}$ HSCs ($EPCR^{High}$ L^- ESLAM HSCs) during a 4-day culture in RPMI-based medium.

(E-H) $EPCR^{High}$ HSC frequency (E, F), total cell number (G), and $EPCR^{High}$ HSC number (H) after the culture under indicated conditions. Numbers in the dot plots represent the percentage within the indicated gate. Data are shown as means ± S.D. (n = 3 biological replicates for the R162 group under SCH treatment and n = 4 biological replicates for all other groups, two independent experiments, *$p < 0.01$ using one-way ANOVA with Tukey's test; Exact *P* values in the Source Data).

(I) The representative histogram showing phosphorylated STAT5 (pY694) level in HSCs after a 1-day culture under conventional (Control), Glud1 inhibition (R162) and Low glutamine (Low Gln) condition. (related to Figure 5K).

Supplementary Figure 8

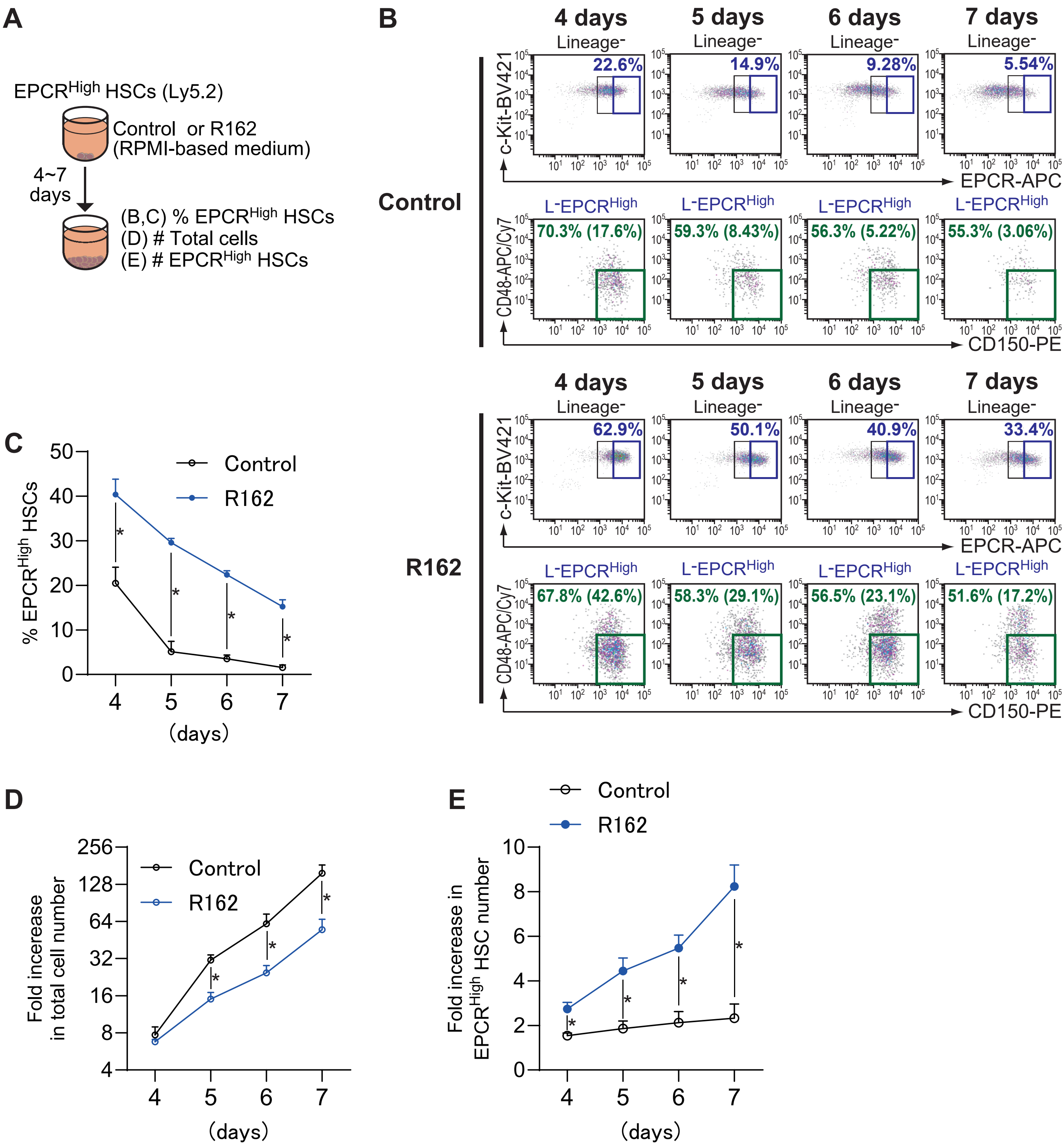

Supplementary Figure 8. The positive effect of R162 on ex vivo expansion of HSCs during more than 5 days of culture.

(A) Strategy to analyze $EPCR^{High}$ HSCs cultured in RPMI-based medium for over 5 days in the absence or presence of R162.

(B-E) The $EPCR^{High}$ $L^{-}ESLAM$ HSC frequency (B and C), total cell number (D), and $EPCR^{High}$ HSC number (E) after indicated period of culture. Numbers or numbers within parentheses in the dot plots represent the percentage within the indicated gate or whole culture cells, respectively. Data are shown as means ± S.D. (n = 4 biological replicates, two independent experiments, *$p < 0.01$ using the two-tailed unpaired *t*-test; Exact *P* values in the Source Data).

Supplementary Figure 9

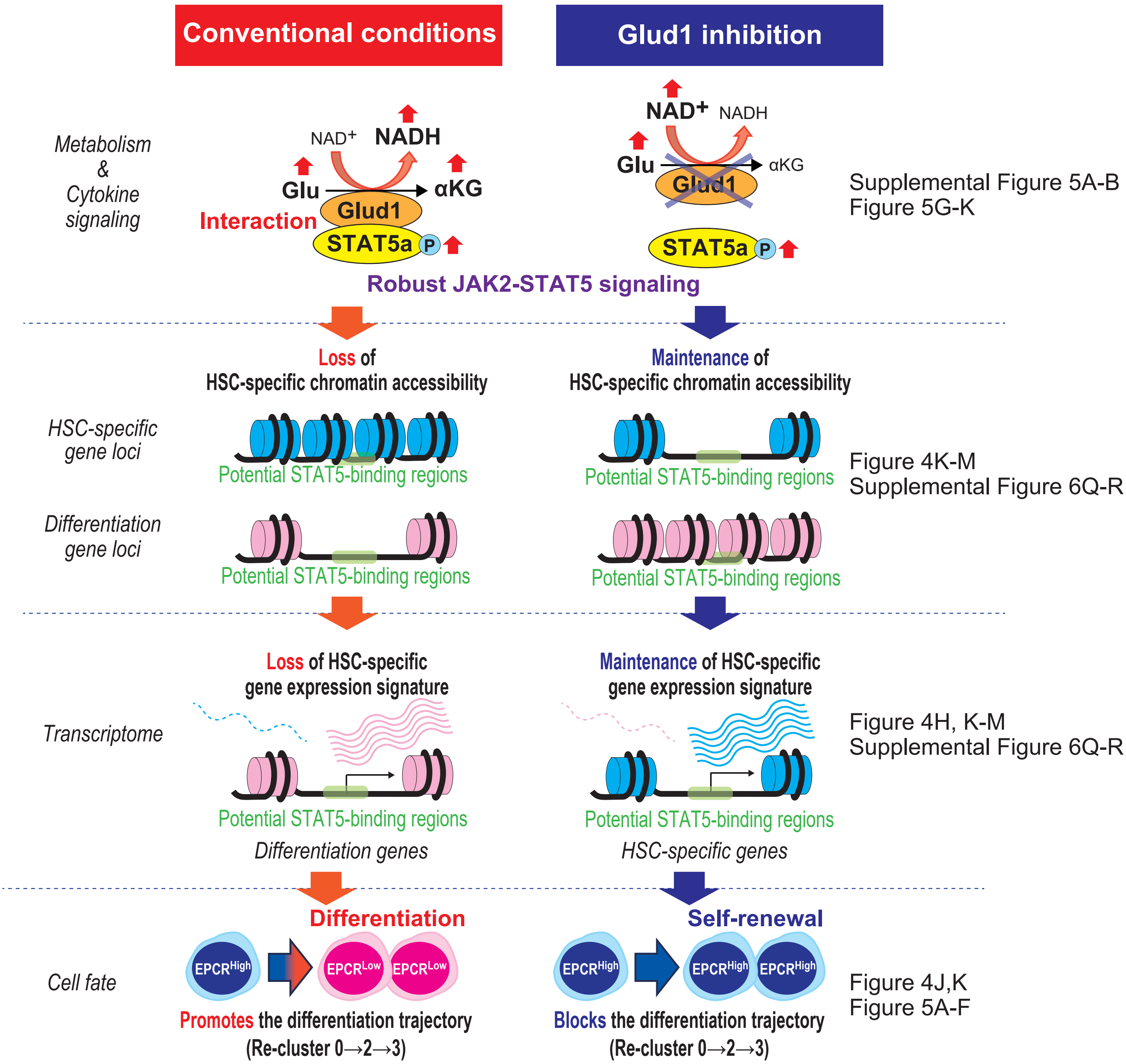

Supplementary Figure 9. Schematic summary of multi-layered cellular states with Glud1 inhibition.

A visual summary of the parallel changes observed across multi-layered cellular states during *ex vivo* HSC culture.

(Left) Under conventional conditions with robust JAK2-STAT5 signaling, Glud1 physically interacts with STAT5a during active glutamate (Glu) catabolism without affecting its phosphorylation. This state is associated with a loss of HSC-specific chromatin accessibility and primitive gene expression signatures near potential STAT5-binding regions, thereby promoting the specific differentiation trajectory (Re-clusters 0→2→3 in Figure 4).

(Right) Conversely, R162-mediated Glud1 inhibition disrupts the Glud1–STAT5a interaction, which is accompanied by altered global metabolite abundance (reduced αKG and an increased NAD^+/NADH ratio). This altered interaction state coincides with the maintenance of HSC-specific chromatin accessibility and the primitive transcriptome, thereby blocking this specific differentiation trajectory and actively guiding HSCs into self-renewal.

Supplementary Table 1. List of antibodies used for flow cytometry

Color	Antigen	Dilution	Clone	Company	Cat #.	Purpose
FITC	Sca-1	1/400	E13-161.7	Biolegend	122506	HSC identification
FITC	Gr-1	1/400	RB6-8C5,	Biolegend	108406,	HSC identification
FITC	CD11b	1/400	M1/70	Biolegend	101206	HSC identification
FITC	TER119	1/400	TER119	Biolegend	116206	HSC identification
FITC	CD45R/B220	1/400	RA3-6B2	Biolegend	103206	HSC identification
FITC	CD3ε	1/400	145-2C11	Biolegend	100306	HSC identification
PE	CD150	1/400	TC15-12F12.2	Biolegend	115904	HSC identification
PE-Cy7	Sca-1	1/400	E13-161.7	Biolegend	122514	HSC identification
APC	EPCR	1/200	eBio1560 (1560)	Thermo Fisher Scientific	17-2012-82	HSC identification,<br>EdC (L^{-}ESLAM), EdC (LEK)
APC-Cy7	CD48	1/400	HM48-1	Biolegend	103432	HSC identification,<br>EdC (L^{-}ESLAM), EdC (LEK)
BV421	c-Kit	1/400	2B8	Biolegend	105828	HSC identification, EdC (LEK)
FITC	CD45.2	1/200	104	Biolegend	109806	Donor cell chimerism
PE	CD45.1	1/200	A20	Biolegend	110708	Donor cell chimerism
PE-Cy7	Gr-1	1/400	RB6-8C5,	Biolegend	108416	Donor cell chimerism
PE-Cy7	CD11b	1/400	M1/70	Biolegend	101216	Donor cell chimerism
APC	CD4	1/400	RM4-5	Biolegend	100516	Donor cell chimerism
APC	CD8a	1/400	53-6.7	Biolegend	100712	Donor cell chimerism
BV421	CD45R/B220	1/400	RA3-6B2	Biolegend	103240	Donor cell chimerism
PerCp-Cy5.5	Gr-1	1/400	RB6-8C5,	Biolegend	108428	EdC (L^{-}ESLAM), EdC (LEK)
PerCp-Cy5.5	CD11b	1/400	M1/70	Biolegend	101228	EdC (L^{-}ESLAM), EdC (LEK)
PerCp-Cy5.5	TER119	1/400	TER119	Biolegend	116226	EdC (L^{-}ESLAM), EdC (LEK)
PerCp-Cy5.5	CD45R/B220	1/400	RA3-6B2	Biolegend	103236	EdC (L^{-}ESLAM), EdC (LEK)
PerCp-Cy5.5	CD3ε	1/400	145-2C11	Biolegend	100328	EdC (L^{-}ESLAM), EdC (LEK)
BV421	CD150	1/400	TC15-12F12.2	Biolegend	115926	EdC (L^{-}ESLAM)
Alexa®488	STAT5(pY694)	1/10	47/Stat5(pY694)	BD Biosciences	612598	STAT5 (pY694)
Alexa®488	Isotype control	1/10	MOPC-21	BD Biosciences	557782	STAT5 (pY694)

Supplementary Table 1. List of antibodies used for flow cytometry

Comprehensive antibody information including specific working dilutions, clone, the manufacturer, catalog numbers, and purpose in this study.

Supplementary Table 2. PCR Primers for ATAC-seq library

Ad1_noMX:	AATGATACGGCGACCACCGAGATCTACACTCGTCGGCAGCGTCAGATGTG
Ad2.1_TAAGGCGA	CAAGCAGAAGACGGCATACGAGATTCGCCTTAGTCTCGTGGGCTCGGAGATGT
Ad2.2_CGTACTAG	CAAGCAGAAGACGGCATACGAGATCTAGTACGGTCTCGTGGGCTCGGAGATGT
Ad2.3_AGGCAGAA	CAAGCAGAAGACGGCATACGAGATTTCTGCCTGTCTCGTGGGCTCGGAGATGT
Ad2.4_TCCTGAGC	CAAGCAGAAGACGGCATACGAGATGCTCAGGAGTCTCGTGGGCTCGGAGATGT
Ad2.5_GGACTCCT	CAAGCAGAAGACGGCATACGAGATAGGAGTCCGTCTCGTGGGCTCGGAGATGT
Ad2.6_TAGGCATG	CAAGCAGAAGACGGCATACGAGATCATGCCTAGTCTCGTGGGCTCGGAGATGT
Ad2.7_CTCTCTAC	CAAGCAGAAGACGGCATACGAGATGTAGAGAGGTCTCGTGGGCTCGGAGATGT
Ad2.8_CAGAGAGG	CAAGCAGAAGACGGCATACGAGATCCTCTCTGGTCTCGTGGGCTCGGAGATGT
Ad2.9_GCTACGCT	CAAGCAGAAGACGGCATACGAGATAGCGTAGCGTCTCGTGGGCTCGGAGATGT
Ad2.10_CGAGGCTG	CAAGCAGAAGACGGCATACGAGATCAGCCTCGGTCTCGTGGGCTCGGAGATGT
Ad2.11_AAGAGGCA	CAAGCAGAAGACGGCATACGAGATTGCCTCTTGTCTCGTGGGCTCGGAGATGT
Ad2.12_GTAGAGGA	CAAGCAGAAGACGGCATACGAGATTCCTCTACGTCTCGTGGGCTCGGAGATGT
Ad2.13_GTCGTGAT	CAAGCAGAAGACGGCATACGAGATATCACGACGTCTCGTGGGCTCGGAGATGT
Ad2.14_ACCACTGT	CAAGCAGAAGACGGCATACGAGATACAGTGGTGTCTCGTGGGCTCGGAGATGT
Ad2.15_TGGATCTG	CAAGCAGAAGACGGCATACGAGATCAGATCCAGTCTCGTGGGCTCGGAGATGT
Ad2.16_CCGTTTGT	CAAGCAGAAGACGGCATACGAGATACAAACGGGTCTCGTGGGCTCGGAGATGT
Ad2.17_TGCTGGGT	CAAGCAGAAGACGGCATACGAGATACCCAGCAGTCTCGTGGGCTCGGAGATGT
Ad2.18_GAGGGGTT	CAAGCAGAAGACGGCATACGAGATAACCCCTCGTCTCGTGGGCTCGGAGATGT
Ad2.19_AGGTTGGG	CAAGCAGAAGACGGCATACGAGATCCCAACCTGTCTCGTGGGCTCGGAGATGT
Ad2.20_GTGTGGTG	CAAGCAGAAGACGGCATACGAGATCACCACACGTCTCGTGGGCTCGGAGATGT
Ad2.21_TGGGTTTC	CAAGCAGAAGACGGCATACGAGATGAAACCCAGTCTCGTGGGCTCGGAGATGT
Ad2.22_TGGTCACA	CAAGCAGAAGACGGCATACGAGATTGTGACCAGTCTCGTGGGCTCGGAGATGT
Ad2.23_TTGACCCT	CAAGCAGAAGACGGCATACGAGATAGGGTCAAGTCTCGTGGGCTCGGAGATGT
Ad2.24_CCACTCCT	CAAGCAGAAGACGGCATACGAGATAGGAGTGGGTCTCGTGGGCTCGGAGATGT

Supplementary Table 2. PCR Primers for ATAC-seq library

The sequence of PCR primers for library amplification in Omni-ATAC-seq.

Supplementary Table 3. The formula of medium used for HSC culture

		(mg/L)
INORGANIC SALTS	Calcium nitrate (Ca(NO3)2 4H2O)	100
	Potassium chloride (KCl)	400
	Magnesium sulfate (MgSO4)	48.84
	Zinc Sulfate Heptahydrate	0.432
	Cupric Sulfate Pentahydrate	0.0013
	Ferric Nitrate Nonahydrate	0.05
	Ferrous Sulfate Heptahydrate	0.417
	Sodium chloride (NaCl)	6000
	Sodium bicarbonate (NaHCO3)	2000
	Sodium Phosphate (Na2HPO4)	800.85
OTHER COMPONENTS	Glutathione Reduced	1
	Phenol red	5
	HEPES	2.38
AMINO ACIDS	L-Arginine	200
	L-Alanine	4.45
	L-Asparagine-H20	50
	L-Aspartic Acid	20
	L-Cystine	50
	L-Glutamic Acid	20
	Glycine	10
	L-Histidine	15
	L-Hydroxyproline	20
	L-Isoleucine	50
	L-Leucine	50
	L-Lysine hydrochloride	40
	L-Methionine	15
	L-Phenylalanine	15
	L-Proline	20
	L-Serine	30
	L-Threonine	20
	L-Tryptophan	5
	L-Tyrosine	20
	L-Valine	20
	L-Glutamine	300
VITAMINS	D-Biotin	0.2
	D-Ca Pantothenate	0.25
	Choline Chloride	3
	Folic Acid	1
	i-Inositol	35
	Niacinamide	1
	p-Aminobenzoic Acid (PABA)	1
	Pyridoxine HCl	1
	Riboflavin	0.2
	Thiamine HCl	1
	Vitamin B12	0.005
	Linoleic acid	0.042
	Hypoxanthine	2.1
	Putrescine+2HCL	0.081
	Thioctic Acid	0.105
	Thymidine	0.365
	Glucose	4500
	Sodium Pyruvate	110

Supplementary Table 3. The status of medium for HSC culture.

RPMI 1640 medium was subjected to minor modification for HSC culture.

SUPPLEMENTARY REFERENCES

1. Wilson, N.K. *et al.* Combined Single-Cell Functional and Gene Expression Analysis Resolves Heterogeneity within Stem Cell Populations. *Cell Stem Cell* **16**, 712-724 (2015).
2. Sommerkamp, P. *et al.* Differential Alternative Polyadenylation Landscapes Mediate Hematopoietic Stem Cell Activation and Regulate Glutamine Metabolism. *Cell Stem Cell* **26**, 722-738 e727 (2020).
