# Supplementary material for: Suppressing glutamate dehydrogenase guides hematopoietic stem cell divisions into self-renewal
Source: Nat Commun. 2026 Sep 5;17:10558. doi: 10.1038/s41467-026-77151-6 (PMC13642937; doi:10.1038/s41467-026-77151-6)
Supplement: Supplementary file 2 [file 41467_2026_77151_MOESM2_ESM.pdf]

Description of Additional Supplementary Files

File Name: Supplementary Data 1
Description: Gene sets for module score analysis. Comprehensive list of gene sets utilized for calculating module scores in single-cell multiome analyses.

File Name: Supplementary Data 2
Description: STAT5-binding sites in blood cells retrieved from ChIP-atlas (https://chip-atlas.org/). Genomic coordinates of STAT5a/b-binding sites in blood cells retrieved from the ChIP-atlas database, utilized to identify potential STAT5-binding peaks in scATAC-seq data.
