# Supplementary material for: Suppressing glutamate dehydrogenase guides hematopoietic stem cell divisions into self-renewal
Source: Nat Commun. 2026 Sep 5;17:10558. doi: 10.1038/s41467-026-77151-6 (PMC13642937; doi:10.1038/s41467-026-77151-6)
Supplement: Supplementary file 3 [file 41467_2026_77151_MOESM5_ESM.pdf]

nature portfolio

Corresponding author(s): Terumasa Umemoto

Last updated by author(s): Jul 15, 2026

Reporting Summary

Nature Portfolio wishes to improve the reproducibility of the work that we publish. This form provides structure for consistency and transparency in reporting. For further information on Nature Portfolio policies, see our Editorial Policies and the Editorial Policy Checklist.

Statistics

For all statistical analyses, confirm that the following items are present in the figure legend, table legend, main text, or Methods section.

n/a	Confirmed	
☐	☒	The exact sample size (*n*) for each experimental group/condition, given as a discrete number and unit of measurement
☐	☒	A statement on whether measurements were taken from distinct samples or whether the same sample was measured repeatedly
☐	☒	The statistical test(s) used AND whether they are one- or two-sided<br>*Only common tests should be described solely by name; describe more complex techniques in the Methods section.*
☐	☒	A description of all covariates tested
☒	☐	A description of any assumptions or corrections, such as tests of normality and adjustment for multiple comparisons
☐	☒	A full description of the statistical parameters including central tendency (e.g. means) or other basic estimates (e.g. regression coefficient) AND variation (e.g. standard deviation) or associated estimates of uncertainty (e.g. confidence intervals)
☐	☒	For null hypothesis testing, the test statistic (e.g. *F*, *t*, *r*) with confidence intervals, effect sizes, degrees of freedom and *P* value noted<br>*Give P values as exact values whenever suitable.*
☒	☐	For Bayesian analysis, information on the choice of priors and Markov chain Monte Carlo settings
☐	☒	For hierarchical and complex designs, identification of the appropriate level for tests and full reporting of outcomes
☐	☒	Estimates of effect sizes (e.g. Cohen's *d*, Pearson's *r*), indicating how they were calculated

Our web collection on statistics for biologists contains articles on many of the points above.

Software and code

Policy information about availability of computer code

Data collection	Flow cytometry: Moflo XDP, CytoFLEX SRT (Beckman Coulter), FACS Canto II (BD Biosciences))<br>NGS: HiSeq 2000, NovaSeq X Plus (Illumina), DNBSEQ-G400 (MGI Tech)<br>Microplate reader: Synergy H1 (BioTeK)<br>Imaging (Confocal microscope): FV1200-IX-KU (Olympus)<br>Imaging (WB): Amersham Imager 600 (GE Healthcare)
Data analysis	Flow cytometry: Kaluza v2.3<br>Gene set enrichment analysis: GSEA v4.3.2<br>RNA-seq:Trimmomatic v0.39, Hisat2 v2.2.1, featureCounts v2.0.1, R-base v4.4.2, DESeq2 v 1.46.0, pheatmap v1.0.13<br>ATAC-Seq:Trimmomatic v0.39, Bowtie2 v 2.4.2, MACS2 v 2.2.7.1, HOMER v4.11, deepTools v3.5.1<br>Deletion check for Crispr/Cas9: CRISPResso2 v2.2.14<br>scMultipme analysis: Cellranger-arc v2.0.2, R v4.4.2, Seurat v5.4.0, Signac v1.16.0, Monocle 3 v1.4.26,CytoTRACE2 v1.1.0, scVelo v0.3.3, chromVAR v1.28.0, JASPAR2020 v0.99.10 ,bedtools v2.30.0<br>Image anlaysis: Fiji software v.1.54p<br>Statistical analysis: GraphPad Prism v11.0.0, R v4.4.2

For manuscripts utilizing custom algorithms or software that are central to the research but not yet described in published literature, software must be made available to editors and reviewers. We strongly encourage code deposition in a community repository (e.g. GitHub). See the Nature Portfolio guidelines for submitting code & software for further information.

Data

Policy information about availability of data

All manuscripts must include a data availability statement. This statement should provide the following information, where applicable:

- Accession codes, unique identifiers, or web links for publicly available datasets
- A description of any restrictions on data availability
- For clinical datasets or third party data, please ensure that the statement adheres to our policy

RNA-seq, ATAC-seq, and scMultiome-seq data are accessible through NCBI Gene Expression Omnibus, accession number GSE279407, GSE279409, GSE338821 and GSE279490.

Research involving human participants, their data, or biological material

Policy information about studies with human participants or human data. See also policy information about sex, gender (identity/presentation), and sexual orientation and race, ethnicity and racism.

Reporting on sex and gender	N/A
Reporting on race, ethnicity, or other socially relevant groupings	N/A
Population characteristics	N/A
Recruitment	N/A
Ethics oversight	N/A

Note that full information on the approval of the study protocol must also be provided in the manuscript.

Field-specific reporting

Please select the one below that is the best fit for your research. If you are not sure, read the appropriate sections before making your selection.

☒ Life sciences ☐ Behavioural & social sciences ☐ Ecological, evolutionary & environmental sciences

For a reference copy of the document with all sections, see nature.com/documents/nr-reporting-summary-flat.pdf

Life sciences study design

All studies must disclose on these points even when the disclosure is negative.

Sample size	No prospective sample-size calculations were performed. Sample size was determined retrospectively to be adequate on the basis of the consistency and magnitude.
Data exclusions	No data was excluded.
Replication	All experiments described in this study were independently reproduced at least two times except for scMultione analysis. scMultione analysis was performed once due to costs.
Randomization	Samples and animals were assigned to groups randomly.
Blinding	Blinding was not performed in the measurements or quantitative analyses (e.g total cell number, the frequency of HSCs, chimerism etc).The researchers were aware of the group to which the sample belonged during data acquisition. However, all experiments were performed in the same workflow.

Reporting for specific materials, systems and methods

We require information from authors about some types of materials, experimental systems and methods used in many studies. Here, indicate whether each material, system or method listed is relevant to your study. If you are not sure if a list item applies to your research, read the appropriate section before selecting a response.

Materials & experimental systems

n/a	Involved in the study	
☐	☒	Antibodies
☐	☒	Eukaryotic cell lines
☒	☐	Palaeontology and archaeology
☐	☒	Animals and other organisms
☒	☐	Clinical data
☒	☐	Dual use research of concern
☒	☐	Plants

Methods

n/a	Involved in the study	
☒	☐	ChIP-seq
☐	☒	Flow cytometry
☒	☐	MRI-based neuroimaging

Antibodies

Antibodies used

Color, Antigen, Dilution, Clone (or Lot# if polyclonal), Company, Cat #., Purpose
FITC, Sca-1, 1/400, E13-161.7, Biolegend, 122506, HSC identification
FITC, Gr-1, 1/400, RB6-8C5, Biolegend, 108406, HSC identification
FITC, CD11b, 1/400, M1/70, Biolegend, 101206, HSC identification
FITC, TER119, 1/400,TER119, Biolegend, 116206, HSC identification
FITC, CD45R/B220, 1/400, RA3-6B2, Biolegend, 103206, HSC identification
FITC, CD3ε, 1/400, 145-2C11, Biolegend, 100306, HSC identification
PE, CD150, 1/400, TC15-12F12.2, Biolegend, 115904, HSC identification
PE-Cy7, Sca-1, 1/400, E13-161.7, Biolegend, 122514, HSC identification
APC, EPCR, 1/200, eBio1560 (1560), Thermo Fisher Scientific, 17-2012-82, "HSC identification, EdC (L-ESLAM), EdC (LEK)"
APC-Cy7, CD48, 1/400, HM48-1, Biolegend, 103432,"HSC identification, EdC (L-ESLAM), EdC (LEK)"
BV421, c-Kit, 1/400, 2B8, Biolegend, 105828,"HSC identification, EdC (LEK)"
FITC, CD45.2, 1/200, 104, Biolegend, 109806, Donor cell chimerism
PE, CD45.1, 1/200, A20, Biolegend, 110708, Donor cell chimerism
PE-Cy7,Gr-1, 1/400, RB6-8C5, Biolegend, 108416, Donor cell chimerism
PE-Cy7,CD11b, 1/400,M1/70, Biolegend, 101216, Donor cell chimerism
APC , CD4, 1/400,RM4-5, Biolegend, 100516, Donor cell chimerism
APC , CD8a, 1/400,53-6.7,Biolegend,100712,Donor cell chimerism
BV421 , CD45R/B220, 1/400, RA3-6B2, Biolegend, 103240, Donor cell chimerism
PerCp-Cy5.5, Gr-1, 1/400, RB6-8C5, Biolegend, 108428, "EdC (L-ESLAM), EdC (LEK)"
PerCp-Cy5.5, CD11b, 1/400, M1/70, Biolegend, 101228,"EdC (L-ESLAM), EdC (LEK)"
PerCp-Cy5.5, TER119, 1/400, TER119, Biolegend, 116226,"EdC (L-ESLAM), EdC (LEK)"
PerCp-Cy5.5, CD45R/B220, 1/400, RA3-6B2, Biolegend, 103236,"EdC (L-ESLAM), EdC (LEK)"
PerCp-Cy5.5, CD3ε, 1/400, 145-2C11, Biolegend,100328, "EdC (L-ESLAM), EdC (LEK)"
BV421, CD150, 1/400, TC15-12F12.2, Biolegend, 115926, EdC (L-ESLAM)
Alexa®488, STAT5(pY694), 1/10, 47/Stat5(pY694), BD Biosciences, 612598, STAT5 (pY694)
Alexa®488, Isotype control, 1/10, MOPC-21, BD Biosciences, 557782, STAT5 (pY694)
Unconjugated, STAT5, 10 μg, A-9, Santa Cruz Biotechnolog, sc-74442, IP
Unconjugated, Isotype control, 10 μg, G3A1, Cell Signaling Technology, 5415S, IP
Unconjugated, Glud1, 1/1000, D9F7P,Cell Signaling Technology 12793T, WB
HRP, rabbit IgG, 1/2000, Lot#34, Cell Signaling Technology, 7074P2 , WB
Unconjugated, STAT5, 1/200, D2O6Y, Cell Signaling Technology, 94205S, PLA
Unconjugated, GLUD1/2, 1/200, Lot#79522760, D2O6Y, Thermo Fisher Scientific, PA5-19267, PLA
Unconjugated, rabbit IgG isotype control, 1/200, DA1E, Cell Signaling Technology, 3900S, PLA
Unconjugated, goat IgG isotype control, 1/200, Lot#ZA-385455, 1/200, Thermo Fisher Scientific,02-6202, PLA
PLUS-oligonucleotide,Rabbit IgG, 1/5, Lot#SLCC5674, Sigma-Aldrich, DUO82002-100RXN, PLA
MINUS-oligonucleotide, Goat IgG, 1/5, Lot#SLCR2992, Sigma-Aldrich, DUO82006-100RXN, PLA

Validation

All antibodies used in our manuscript have been validated by provided manufacturers or previous publications.

Eukaryotic cell lines

Policy information about cell lines and Sex and Gender in Research

Cell line source(s)

A-6 (ES-derived) cells (RCB1517) were originally obtained from the RIKEN BioResource Research Center (BRC).sx

Authentication

Cell line authentication was not additionally performed after receipt.

Mycoplasma contamination

According to the provider, the cell line tested negative for mycoplasma contamination.

Commonly misidentified lines
(See ICLAC register)

This cell line is not listed in the ICLAC database of commonly misidentified cell lines.

Animals and other research organisms

Policy information about studies involving animals; ARRIVE guidelines recommended for reporting animal research, and Sex and Gender in Research

Laboratory animals	C57BL/6-Ly5.2 and C57BL/6-Ly5.1 mice were obtained from Japan SLC Inc. (Shizuoka, Japan) and Sankyo Lab Service Corporation (Tokyo, Japan), respectively. Each strain used was between 8 and 12 weeks of age. All mice were housed in a specific-pathogen-free (SPF) facility with ad libitum access to food and water under controlled environmental conditions: a 12-h light/12-h dark cycle, an ambient temperature of 22 ± 2 °C, and a relative humidity of 50 ± 10%.
Wild animals	N/A
Reporting on sex	Donor HSCs were isolated exclusively from male C57BL/6 mice to ensure a uniform stem cell pool, avoiding hormonal fluctuations. Both male and female mice were used as transplant recipients. Sex was not considered as an experimental variable because the study focused strictly on the intrinsic repopulating capacity of the donor cells, with recipients functioning solely as the in vivo engraftment environment. Data from all recipients were therefore pooled for the analyses.
Field-collected samples	N/A
Ethics oversight	The study protocol was formally reviewed and approved by the Institutional Animal Care and Use Committee of Kumamoto University (Approval/Protocol Number: A2023-058, A2025-076).

Note that full information on the approval of the study protocol must also be provided in the manuscript.

Plants

Seed stocks	N/A
Novel plant genotypes	N/A
Authentication	N/A

Flow Cytometry

Plots

Confirm that:

☒ The axis labels state the marker and fluorochrome used (e.g. CD4-FITC).

☒ The axis scales are clearly visible. Include numbers along axes only for bottom left plot of group (a 'group' is an analysis of identical markers).

☒ All plots are contour plots with outliers or pseudocolor plots.

☒ A numerical value for number of cells or percentage (with statistics) is provided.

Methodology

Sample preparation	*Describe the sample preparation, detailing the biological source of the cells and any tissue processing steps used.*
Instrument	Cells were analyzed or sorted by Moflo XDP or CytoFLEX SRT (Beckman Coulter).
Software	Collected data (FCS files) were analyzed on Kaluza software (Beckman Coulter).
Cell population abundance	Since HSCs are a rare population, it is not realistic to check the purity of sorted cells at every time. Conversely, we checked sorting purity and efficiency as a constantly pilot experiment
Gating strategy	We always prepared negative control samples stained with isotype control antibodies to decide all gating.

☒ Tick this box to confirm that a figure exemplifying the gating strategy is provided in the Supplementary Information.
