# Supplementary material for: Suppressing glutamate dehydrogenase guides hematopoietic stem cell divisions into self-renewal
Source: Nat Commun. 2026 Sep 5;17:10558. doi: 10.1038/s41467-026-77151-6 (PMC13642937; doi:10.1038/s41467-026-77151-6)
Supplement: Supplementary file 4 [file 41467_2026_77151_MOESM6_ESM.pdf]

nature portfolio

Peer Review File

Suppressing glutamate dehydrogenase guides hematopoietic stem cell divisions into self-renewal

Corresponding Author: Dr Terumasa Umemoto

This file contains all reviewer reports in order by version, followed by all author rebuttals in order by version.

Parts of this Peer Review File have been redacted as indicated to maintain the confidentiality of unpublished data.

Version 0:

Reviewer comments:

Reviewer #1

(Remarks to the Author)
This manuscript (Umemoto et al.,) presents an intriguing biological hypothesis in which modulation of Glud1 activity influences haematopoietic stem cell (HSC) states across metabolic, signaling, and chromatin layers. The authors propose that changes in glutamate–αKG metabolism, a potential non-canonical interaction between Glud1 and STAT5a, and alterations in differentiation trajectories collectively form a coherent mechanistic model. However, much of the evidence offered remains within the realm of prediction and assumption rather than demonstrated causality, and the integrative mechanism presented is, at present, still largely hypothetical.
The central concept—that Glud1 may connect amino-acid metabolism with stem-cell state regulation through unconventional signaling roles—is genuinely novel and could ultimately prove impactful. With appropriate additional data to strengthen mechanistic depth, the manuscript has the potential to become substantially more compelling. In this sense, although a significant amount of work is still required, I believe that depending on the authors' revisions, a path towards Major Revision—and possibly even Minor Revision—remains open. Strengthening metabolic flux analysis, proteomics, and epigenetic links would considerably enhance the manuscript's scientific solidity.

Major Comments
The manuscript proposes a model in which Glud1 regulates HSC biology through interconnected metabolic, signalling, and chromatin pathways, yet the current dataset does not provide sufficient mechanistic depth to support such a multi-layered framework.
• The authors focus heavily on glutamate and αKG metabolism but do not provide absolute quantification of these metabolites, nor do they employ ^13C-labelled tracers, making it impossible to evaluate actual Glu→αKG flux.
• Without direct metabolic flux measurements, it remains uncertain whether Glud1 inhibition genuinely alters αKG production, TCA cycle throughput, or downstream metabolic pathways in a manner that could causally influence HSC state transitions.
• The metabolic interpretation relies on relative abundance measurements rather than comprehensive metabolomics, making it difficult to discern whether transcriptional changes arise from true metabolic modulation or from secondary stress responses.
• The manuscript suggests that Glud1 interacts with STAT5a and influences its function, yet no phospho-proteomic analysis or quantitative assessment of STAT5a phosphorylation dynamics is presented to substantiate this claim at the protein level.
• The absence of proteome-wide data prevents assessment of whether Glud1 inhibition induces broader rewiring of metabolic enzymes, signaling cascades, or post-translational modifications relevant to HSC regulation.
• No experiments investigate whether changes in αKG availability affect epigenetic dioxygenases such as TET or JmjC, leaving a major conceptual gap in linking amino acid metabolism to chromatin remodeling.
• Epigenetic outputs—including alterations in 5hmC or histone methylation—are not assessed, despite their central relevance to how metabolic perturbations shape stem-cell identity.
• Although multimode and ATAC-seq data are presented, these are not integrated with metabolic or proteomic findings, resulting in a fragmented mechanistic narrative that does not convincingly connect the three layers.
• The reported chromatin accessibility differences are modest and not attributed to defined metabolic or signaling inputs, weakening the argument that Glud1 modulates chromatin structure in a functionally meaningful manner.
• The pseudo time trajectory analysis appears reliant on predefined phenotypic markers rather than unbiased metabolic or proteomic features, raising the possibility of circular reasoning in defining the "primitive" HSC state.
• The use of extremely low-glutamine culture conditions likely imposes non-physiological metabolic stress, yet the

manuscript does not examine how such artificial constraints themselves affect amino-acid handling, proteome stability, or HSC state independent of Glud1.
• The manuscript currently depends too heavily on transcriptional signatures, and would benefit from a more integrated mechanistic narrative connecting metabolism, protein-level signaling, and epigenetic regulation.
• Overall, although the conceptual hypothesis is intellectually appealing, the lack of depth across metabolic, proteomic, and epigenetic dimensions prevents the authors from presenting a fully substantiated biological mechanism at this stage.

Reviewer #2

(Remarks to the Author)
Despite multiple efforts and success to optimize in vitro culture conditions, expansion of hematopoietic stem cells (HSCs) while remaining their stemness capacity still remains a challenge. In this manuscript they discover that suppression of Glud1, a key enzyme for glutamate metabolism, in vitro supports the maintenance and expansion of functional HSCs. The mechanism of Glud1 inhibition on HSC self-renewal maintenance is coupled to JAK-STAT signalling. The observations are interesting and will have a great impact on improving in vitro culture conditions for functional HSCs.

- The data in Figure 1 on EPCRhi cells are interesting, however several groups have by now shown EPCR high expressing stem cells to be the most potent one, so the data is not really novel.
- Figure 2B, 5FU data: only absolute numbers of EPCRhi HSCs are shown over time. However, it would be informative to also show these analysis for all opther HSC populations for which the Glu and EdC analysis are shown in figure 2D and 2E
- Figure 3B-F: Glud1 inhibition results in elevated frequency of EPCRhi HSCs without reducing total cell numbers: what happens to the composition of the cultures in the presence of Glud1 inhibition regarding progenitor cell numbers, differentiation?
- Figure 4: both re-clusters 1 and 2 show significant enrichment of JAK-STAT signaling, however for cluster 1 this results in HSC maintenance and for cluster 2 a reduction in numbers. How do the authors explain these differences, when JAK-STAT signaling should be leading here?
- Figure 5: JAK inhibitors alone already result in reduced total numbers (Figure 5E) and a reduction in the fold increase in EPCRhi HSCs (Figure 5F): thus, the effect of inhibiting JAK/STAT signalling alone is already dominant. The combined treatment showing the same as JAK inhibition alone can thus solely be the result of JAK inhibition, and not necessarily show that Glud1 effects are via JAK/STAT.
- The ip and WB in figure 5G is not convincing. There might be a very weak band…
- It is too bad that the authors are not able to perform R162 treatment in the PVA cultures. Did they check the Glu levels in PVA cultures? How are they compared to the levels in their conventional cultures?
- The longterm cultures in Figure 7 are interesting. Is there a change that one starts to select for specific subclones and thus is looking a clonal expansion?

Expansion of mouse HSCs is interesting however the main clinical interest is with human HSCs. Therefore the impact of the story would significantly increase if human cultures were tested for Glu levels and inhibition of Glud1 would be tested in these human cultures.

Reviewer #3

(Remarks to the Author)
1) Summary and overall assessment

In this manuscript, Hashimoto and colleagues identify the glutamate dehydrogenase (Glud1) inhibitor R162 as an efficient strategy to enhance self-renewal divisions while preserving a bona fide HSC phenotype, both transcriptionally and functionally. The major impact of the work lies in the identification of a novel compound and metabolic strategy that enables robust expansion of murine phenotypic HSCs ex vivo.

The central claims of the study are, in my view, well supported by the data presented:

Low-glutamine culture conditions preserve HSC identity ex vivo but do not efficiently drive expansion.

Genetic (sgGlud1) or pharmacological (R162) inhibition of Glud1 leads to a marked increase in phenotypic and functional HSCs, accompanied by true expansion.

STAT5 appears to physically and functionally interact with Glud1, supporting a metabolic–transcriptional coupling mechanism that is well aligned with existing literature.

Combining R162 with PVA-F12 culture conditions further enhances LT-HSC expansion, likely defining a new best-in-class medium composition for the ex vivo expansion of multipotent HSCs.

This last point alone makes it highly likely that the manuscript will be widely used and cited by the stem cell community.

2) Major comments

Clarity regarding culture media

Throughout the manuscript, several distinct basal media are used in R162 experiments (RPMI-based versus F12-PVA-based formulations). It would greatly improve clarity if each figure (or at least each experiment) explicitly stated which basal medium was used. This is particularly important given that media composition clearly modulates the magnitude of the observed effects.

DMSO controls

I did not find any explicit mention of DMSO being used as a vehicle control, nor the final DMSO concentration employed. Given that R162 is dissolved in DMSO, this information is important to report clearly in the Methods.

This point is not merely technical: in our own hands (unpublished observations), 0.1% DMSO causes mild but reproducible effects on LT-HSC expansion. Interestingly, this aligns with recent reports describing radioprotective effects of DMSO mediated through LT-HSCs (e.g. Free Radic Biol Med 2020; DOI: 10.1016/j.freeradbiomed.2020.03.021). I am confident the appropriate controls were performed, but explicitly stating the vehicle control and DMSO percentage would strengthen rigor and reproducibility.

Extension to human HSCs

The effects of R162 on murine HSC expansion are sufficiently strong that it is difficult not to wonder whether similar outcomes would be observed in human HSC cultures. Even a limited set of exploratory experiments using human cells—well within the technical scope of this excellent group—would substantially enhance the impact and translational relevance of the study.

NAD^+ metabolism and NR supplementation

The authors show that R162 treatment or sgGlud1 increases NAD^+ levels in HSCs, yet supplementation with nicotinamide riboside (NR) does not phenocopy these effects. It would be helpful to know whether the NR treatment used here actually increases intracellular NAD^+ levels in HSCs. If this is already established in the literature, citing it would help; otherwise, directly measuring NAD^+ following NR treatment would clarify interpretation.

Single-cell analysis of R162-treated HSCs

R162-treated HSCs clearly undergo a substantial shift in cell state. While the clustering and enrichment analyses are informative, several aspects could be strengthened:

Replicates: There is no explicit mention of biological replicates for the single-cell experiments. If replicates were not included, conclusions drawn from these analyses should be appropriately tempered.

HSC subsetting: When defining HSC clusters, it is unclear why cluster 5 was excluded. This cluster appears Cd48^low and expresses Procr, and supplementary figures show expression of established HSC markers such as Mecom, Mllt3, Itsn1, and Mir99ahg. The high expression of Top2a and Mki67 suggests these may represent cycling HSCs. Applying cell-cycle correction or explicitly discussing this population would strengthen the analysis.

STAT-related programs: Despite the proposed STAT5–Glud1 interaction, there is no focused analysis of STAT target gene expression or chromatin accessibility. Given that scMultiome data were generated, a deeper interrogation of differentially accessible regions and transcription factor motifs would be highly valuable. This dataset is particularly important, as high-quality scMultiome data from HSC cultures remain relatively scarce.

Non-HSC populations: The remaining clusters appear to represent various activated HSC/MPP states and megakaryocyte-biased progenitors. Were any notable changes observed in these compartments upon R162 treatment?

Global $Procr^+$ frequency: Across the full dataset, were Procr-expressing clusters enriched in the R162 condition?

HSC state signatures: Additional analyses examining R162-induced changes in established HSC signatures (dormancy, cell cycle, lineage bias, etc.) would further solidify the mechanistic conclusions.

3) Minor comments

The Methods section describing NAD^+ and αKG measurements states “following manufacturer’s protocol,” but the reagent kits used are not specified.

An input control appears to be missing from the immunoprecipitation experiments.

Several antibody fluorochromes are not listed in the Methods.

4) Typographical errors

Line 534: “The resulting IP samples was analyzed by SDS-PAGE” → should be were.

Figure 7K: “The frequency of multi-lineage engfarted mice” → should be engrafted.

Version 1:

Reviewer comments:

Reviewer #1

(Remarks to the Author)
The manuscript has been clearly improved compared to the initial submission, and the authors are to be commended for their careful and constructive response to the reviewers’ comments. In particular, the inclusion of additional data and the reorganization of the text have substantially strengthened the reproducibility and overall persuasiveness of the central observation that Glud1 inhibition contributes to the maintenance and expansion of HSC. The authors have also appropriately tempered their interpretation of the Glud1 and STAT5a interaction, reframing it as a correlative association rather than a definitive mechanistic driver, which helps avoid overstatement. Furthermore, the additional validation of the dependence on JAK–STAT signaling provides important functional support and clarifies the biological relevance of the findings.
But, despite these improvements, the central mechanistic framework of the study remains insufficiently defined. In the revised manuscript, multiple layers including metabolic changes αKG and NAD^+/NADH, the Glud1 and STAT5a interaction, and alterations in chromatin and transcriptional states are presented, but it is still unclear how these are causally integrated. As a result, while each dataset is individually interesting, the study does not yet converge on a single, coherent mechanistic explanation, leaving the overall message somewhat difficult to interpret.

A particularly important issue is that it remains unclear whether the effects of Glud1 inhibition reflect active instruction of cell fate or, alternatively, the selective preservation or expansion of a pre-existing HSC subset. This distinction has direct implications for the interpretation of the results and should be addressed either experimentally or through a more explicit and balanced discussion. In addition, the conclusion that the observed effects are independent of αKG appears to be based primarily on the exclusion of global metabolic changes, without sufficiently considering the possibility of localized or complex-dependent metabolic regulation.

Reviewer #2

(Remarks to the Author)
Thank you to the authors for responding to my comments and suggestions. The manuscript greatly improved upon revision. Just a few remarks in response:

- Regarding my point to Figure 3B-F: if the absolute pool of EPCRlow cells is reduced, is the composition within this pool the same? Do you have similar levels of the more committed progenitors or is there a bias towards one of the other? EPCRlow cells is a great mix of different cell populations.
- The additional detailed analysis on Glud1-STAT5a in figures 4 and 5 looks really interesting. As the authors conclude it does not uncover the whole mechanism yet, but it definitely increases the impact of their story
- The additional data on ERK inhibition still does not formally proof the point raised regarding the potential confounding effect. However, this will also be a difficult one to dissect.

Reviewer #3

(Remarks to the Author)
Hashimoto et al. have substantially revised their manuscript on Glud1, addressing all of my comments. I am convinced that the strength of the findings has been improved by the modifications. Simultaneously, I think the authors have tempered comments where the evidence was less robust. Still, I think there is substantial cross validation of the key results with various approaches. Thus, I remain convinced about the main conclusions and think that Glud1 inhibition. My main reservation is the lack of validation in human cultures, but the authors discuss a reasonable path forward for progress in translation. Overall, I think the manuscript has been improved leaps and bounds and the impact of the findings warrant publication and discussion by the community.

Version 2:

Reviewer comments:

Reviewer #1

(Remarks to the Author)
The revised MS has reached a standard where it provides useful information to the readers of this journal.

Reviewer #2

(Remarks to the Author)
Thank you to the authors for responding to my remarks. This is an interesting manuscript that will attract great attention in the field.

Open Access This Peer Review File is licensed under a Creative Commons Attribution 4.0 International License, which permits use, sharing, adaptation, distribution and reproduction in any medium or format, as long as you give appropriate credit to the original author(s) and the source, provide a link to the Creative Commons license, and indicate if changes were made.
In cases where reviewers are anonymous, credit should be given to 'Anonymous Referee' and the source.
The images or other third party material in this Peer Review File are included in the article's Creative Commons license, unless indicated otherwise in a credit line to the material. If material is not included in the article's Creative Commons license and your intended use is not permitted by statutory regulation or exceeds the permitted use, you will need to obtain permission directly from the copyright holder.
To view a copy of this license, visit https://creativecommons.org/licenses/by/4.0/

We would like to thank the reviewers for careful and constructive criticism of our paper entitled "Suppressing glutamate dehydrogenase guides hematopoietic stem cell divisions into self-renewal". To address the reviewers' comments and strengthen the study, we have performed additional experiments and incorporated new data. Moreover, we have reorganized the manuscript and revised the text to improve clarity and presentation. In the revised manuscript, all new data, text, and updated figure numbers are highlighted in red. Our point-by-point responses to the reviewers' suggestions and critiques are provided below

Reviewer #1

This manuscript (Umemoto et al.,) presents an intriguing biological hypothesis in which modulation of Glud1 activity influences haematopoietic stem cell (HSC) states across metabolic, signaling, and chromatin layers. The authors propose that changes in glutamate–αKG metabolism, a potential non-canonical interaction between Glud1 and STAT5a, and alterations in differentiation trajectories collectively form a coherent mechanistic model. However, much of the evidence offered remains within the realm of prediction and assumption rather than demonstrated causality, and the integrative mechanism presented is, at present, still largely hypothetical.

The central concept—that Glud1 may connect amino-acid metabolism with stem-cell state regulation through unconventional signaling roles—is genuinely novel and could ultimately prove impactful. With appropriate additional data to strengthen mechanistic depth, the manuscript has the potential to become substantially more compelling. In this sense, although a significant amount of work is still required, I believe that depending on the authors' revisions, a path towards Major Revision—and possibly even Minor Revision—remains open. Strengthening metabolic flux analysis, proteomics, and epigenetic links would considerably enhance the manuscript's scientific solidity.

We thank the reviewer for their careful review of our manuscript and for providing constructive and insightful advice. Despite the identification of numerous factors critical for hematopoietic stem cell (HSC) self-renewal, the *ex vivo* maintenance and expansion of murine HSCs remain a significant challenge. While the use of polyvinyl alcohol (PVA) instead of bovine serum albumin (BSA) has recently enabled the *ex vivo* expansion of functional mouse HSCs (Wilkinson er al, *Nature,* 2019, 571(7763): 117-121), alternative methodologies remain scarce. In this context, the primary contribution of our study is the demonstration that Glud1 inhibition enables long-term culture and functional expansion of mouse HSCs under conventional, serum-free conditions using BSA. Notably, the expansion efficiency in our culture system exceeds that of existing methods, including the PVA-based system.

We agree with the reviewer that metabolic flux analysis would provide higher metabolic resolution. However, performing such assays on primary HSCs is technically prohibitive due to extreme cell scarcity; a single mouse yields only ~10^4 HSCs, whereas standard isotope-tracing assays typically require at least 1 x 10^6 cells per sample. Importantly, our data indicate that the beneficial effect of Glud1 inhibition on HSC maintenance is independent of changes in global αKG abundance or the NAD^+/NADH ratio, a finding further supported by additional data in the revised version (Figure S4). Therefore, we believe that further investigation into global αKG availability or NAD^+/NADH ratios is unlikely to elucidate the primary mechanism by which Glud1 inhibition supports HSC maintenance. Accordingly, we specified this important conclusion in Results and Discussion sections of the revised manuscript (Page 9, line 219–221 and Page 16, line 379–381).

Instead, our integrated scMultiome data indicated that the molecular framework supporting HSC identity—at both the transcriptional and epigenetic levels—relies on a synergy between Glud1-mediated regulation and the active JAK–STAT signaling axis through additional analyses (Figures 4 and S5). Based on these observations, we confirmed that the beneficial effect of Glud1 inhibition on HSC maintenance is functionally dependent on robust JAK–STAT signaling (Figure 5A–F). Furthermore, we observed that Glud1 interacts with STAT5a in HSCs, and that this interaction is suppressed by Glud1 inhibition or low glutamine (Gln) conditions without affecting STAT5 phosphorylation levels (Figure 5H–K). Collectively, our findings exhibited a distinct HSC state in which self-renewal capacity is maintained through a combination of robust JAK–STAT activity and the catabolism-dependent modulation of the Glud1–STAT5a association. While we acknowledge that proteomic profiling could further substantiate this mechanism, the current dataset focusing on JAK–STAT signaling in the revised version is sufficient to demonstrate a linkage between Glud1-mediated glutamate (Glu) catabolism and STAT-dependent HSC regulation.

As pointed out by the reviewer, the precise molecular mechanism by which the Glud1–STAT5a association regulates HSC maintenance warrants further investigation. Therefore, we have addressed this in the revised Discussion by clarifying the current interpretative limits and highlighting the need for future multifaceted investigations (Page 16, line 399–Page 17, line 402). Moreover, we have tempered our interpretation of the Glud1–STAT5a association throughout the manuscript, characterizing it as a correlative cellular state associated with self-renewal rather than a confirmed mechanistic driver (e.g. Page 2, line 62–65; Page 4, line 110–113; Page 13, line 306–309; Page 18, line 434–436). Nevertheless, we believe our findings provide significant insights into JAK–STAT-mediated HSC regulation and offer a potent strategy for *ex vivo* HSC expansion.

Major Comments

The manuscript proposes a model in which Glud1 regulates HSC biology through interconnected metabolic, signalling, and chromatin pathways, yet the current dataset does not

provide sufficient mechanistic depth to support such a multi-layered framework.

• The authors focus heavily on glutamate and αKG metabolism but do not provide absolute quantification of these metabolites, nor do they employ ^13C-labelled tracers, making it impossible to evaluate actual Glu→αKG flux.

We agree with the reviewer that absolute metabolite quantification and metabolic flux analysis are valuable for confirming the precise enzymatic impact of R162 on HSCs. Regarding absolute quantification, we have already provided the absolute levels of intracellular αKG in our manuscript, confirming a significant decrease upon R162 treatment (Figure S4A and B). We also examined intracellular Glu levels but found that Glud1 inhibition did not significantly alter them (see Response Figure A below). Since the intracellular pool of Glu is approximately 100-fold larger than that of αKG, any decrease in this conversion would have a much more noticeable impact on αKG levels than on total Glu levels. Thus, absolute quantification alone remains insufficient to evaluate the precise Glu-to-αKG flux.

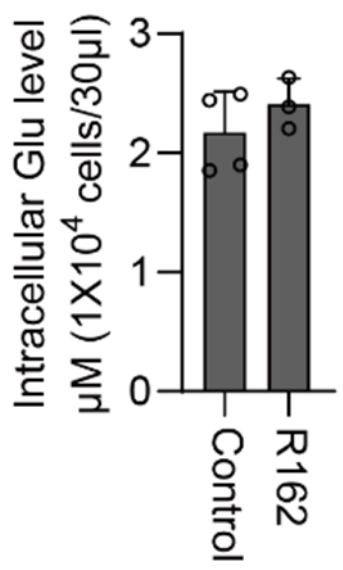

Response Figure A. The effect of R162 on intracellular Glu level in HSCs
After HSCs were cultured for 1-day in the absence or presence of R162, intracellular Glu level in HSCs were examined.

We acknowledge that ^{13}C-isotope tracing would be the ideal method to evaluate this flux directly. However, performing such analysis on primary HSCs is technically prohibitive due to their extreme scarcity; a single mouse yields only ~10^4 HSCs, whereas standard flux analysis typically requires at least 1 x 10^6 cells per sample. Consequently, we have focused our interpretation on the observed decrease in αKG levels and the increased NAD^+/NADH ratio (Figure S4A and B), rather than on the total conversion rate from Glu to αKG. We have updated the corresponding sections in the manuscript to clarify this scope (Page 8, Line 208–210).

Importantly, our functional data indicate that the positive effect of Glud1 inhibition on HSC maintenance is not driven by changes in global αKG abundance. Supplementation with dimethyl-αKG (dmαKG) successfully restored αKG levels in R162-treated cells but failed to abolish or influence the effect on HSC maintenance (Figure S4A and C–G). Furthermore, inhibition of transaminase activity by AOA reduced intracellular αKG levels to an extent similar to R162, yet did not contribute to HSC maintenance (Figures 3A–H and S4A). These results strongly suggest that the beneficial effects of R162 are not causally linked to global αKG availability. Therefore, we believe the central conclusions of our study remain solid despite the absence of isotope-based flux data. In the revised version, we have incorporated new data regarding intracellular αKG levels and the NAD^+/NADH ratio into Figure

S4A and S4B respectively. Accordingly, we have updated the corresponding explanations and explicitly stated this conclusion in the Results and Discussion section (Page 9, line 211−221 and Page 16, line 379−381).

• Without direct metabolic flux measurements, it remains uncertain whether Glud1 inhibition genuinely alters αKG production, TCA cycle throughput, or downstream metabolic pathways in a manner that could causally influence HSC state transitions.

We agree that direct metabolic flux measurements would provide definitive resolution regarding the impact of Glud1 inhibition on αKG production and TCA cycle throughput. However, our experimental data strongly suggest that the changes in global αKG abundance induced by R162 are not the primary drivers of the observed HSC maintenance. As demonstrated in our study, restoring αKG levels through the supplementation of dimethyl-αKG did not abolish or significantly alter the positive effect of Glud1 inhibition on HSC maintenance (Figure S4A and C–G). Furthermore, while the inhibition of transaminases by AOA reduced intracellular αKG to a similar extent as R162, it failed to promote HSC maintenance (Figures 3A–H and S4A). These results strongly indicated that the beneficial effects of R162 are not causally linked to changes in global αKG availability. Instead, through integrated scMultiome analyses, we identified a non-canonical role of Glud1 associated with robust JAK–STAT signaling. While isotope-based flux analysis would undoubtedly offer additional metabolic resolution, we believe it is not required to support the central conclusion of this study—that Glud1 maintains HSC stemness via a mechanism independent of global αKG flux.

• The metabolic interpretation relies on relative abundance measurements rather than comprehensive metabolomics, making it difficult to discern whether transcriptional changes arise from true metabolic modulation or from secondary stress responses.

As noted in our previous responses, our data indicate that changes in global αKG abundance are not responsible for the positive effect of Glud1 inhibition on HSC maintenance. Consequently, we do not consider the decrease in global αKG abundance to be the primary trigger for the transcriptional changes observed upon Glud1 inhibition.

To further address the reviewer's concern regarding secondary stress, we conducted a Gene Set Enrichment Analysis (GSEA) using our transcriptome data to assess whether Glud1 inhibition induces generalized cellular stress responses. We confirmed that the transcriptional programs observed were not consistent with stress-related pathways, including reactive oxygen species (ROS), oxidative stress, the unfolded protein response (UPR), apoptosis, or p53 activation (see Table A for reviewers below). While we acknowledge that comprehensive metabolomics could further refine the metabolic landscape, these transcriptional findings strongly suggest that the observed changes are a direct result of Glud1-mediated modulation rather than secondary stress responses.

Upregulated in Control

NAME	ES	NES	NOM p-val	FDR q-val	FWER p-val
HALLMARK_REACTIVE_OXYGEN_SPECIES_PATHWAY	0.375709	1.2935724	0.1459566	0.97278607	0.741
HALLMARK_PEROXISOME	0.25477397	1.1448358	0.17933723	1	0.95
HALLMARK_UNFOLDED_PROTEIN_RESPONSE	0.22043443	0.9960101	0.38947368	0.9366462	1
HALLMARK_UV_RESPONSE_DN	0.25095275	0.9936322	0.49309665	0.8840975	1
HALLMARK_APOPTOSIS	0.18631504	0.8553297	0.8669355	0.941357	1

Upregulated in R162

NAME	ES	NES	NOM p-val	FDR q-val	FWER p-val
HALLMARK_DNA_REPAIR	-0.26589382	-1.0403824	0.45472836	1	1
HALLMARK_HYPOXIA	-0.2457372	-1.0124602	0.38574424	0.8265297	1
HALLMARK_P53_PATHWAY	-0.23204556	-0.98776436	0.48247424	0.74893326	1
HALLMARK_UV_RESPONSE_UP	-0.24180828	-0.96879554	0.47887325	0.7344573	1
HALLMARK_INFLAMMATORY_RESPONSE	-0.19754864	-0.9265514	0.654321	0.73879033	1

ES: Enrichment Score

NES: Normalized enrichment Score

Table A for reviewers. The effect of R162 on stress response in HSCs.
After HSCs were cultured for 1 day in the absence (control) or presence of R162, gene expression profiles were analyzed by RNA-Seq. Using these transcriptome data and gene sets related to stress responses, gene set enrichment analysis were performed to assess whether Glud1 inhibition induces secondary stress responses.

• The manuscript suggests that Glud1 interacts with STAT5a and influences its function, yet no phospho-proteomic analysis or quantitative assessment of STAT5a phosphorylation dynamics is presented to substantiate this claim at the protein level.

We have already demonstrated that R162 treatment or low-Gln conditions disrupt the physical association between Glud1 and STAT5a in HSCs (Figure 5H–J) without significantly affecting STAT5 phosphorylation levels (Figure 5K). Importantly, Glud1 inhibition with R162 promotes functional HSC maintenance (Figure 3). Moreover, re-analysis of our integrated scMultiome data indicates that the molecular framework supporting HSC identity—at both the transcriptional and epigenetic levels—relies on a synergy between Glud1-mediated regulation and the active JAK–STAT signaling axis (Figure 4 and S5). These results suggest that Glud1 influences STAT5 function via a non-canonical, phosphorylation-independent mechanism involving their physical association, although the precise molecular basis of this regulation remains to be fully elucidated.

In response to the reviewer's suggestion, we have now explicitly addressed the potential nature of this interpretation and highlighted the necessity for further multifaceted investigations in the revised Discussion (Page 16, line 399–Page 17, line 402). Furthermore, we have tempered our interpretation of the Glud1–STAT5a association throughout the manuscript, characterizing it as a correlative cellular state associated with self-renewing divisions rather than a definitive mechanistic driver (e.g. Page 2, line 62–65; Page 4, line 110–113; Page 13, line 306–309; Page 18, line 434–436).

• The absence of proteome-wide data prevents assessment of whether Glud1 inhibition induces broader rewiring of metabolic enzymes, signaling cascades, or post-translational modifications relevant to HSC regulation.

We agree that proteome-wide analyses would provide additional insight into the potential global rewiring of metabolic enzymes, signaling pathways, and post-translational modifications upon Glud1 inhibition. However, our findings suggest that the central mechanism supporting HSC maintenance is highly focused rather than dependent on broad proteomic remodeling.

Indeed, to screen for changes relevant to HSC maintenance, we initially performed bulk transcriptome comparisons. However, these revealed no extensive or characteristic global changes in HSCs upon Glud1 inhibition, as evidenced by the limited number of differentially expressed genes and the absence of significantly enrichened gene sets (see Response Figure B and Table B below). This suggests that Glud1 inhibition does not induce large-scale transcriptomic or metabolic rewiring. To achieve the high resolution necessary for capturing subtle transcriptional and chromatin-state changes, we utilized integrated scMultiome profiling. This approach indicated that the molecular framework supporting HSC identity relies on a specific synergy between Glud1-mediated regulation and the active JAK–STAT signaling axis (Figures 4 and S5). Consistent with this, we validated that the beneficial effects of Glud1 inhibition are functionally dependent on robust JAK–STAT signaling using JAK2 inhibitors (Figure 5A–F). Furthermore, Glud1 inhibition suppressed the physical Glud1–STAT5a interaction without affecting STAT5 phosphorylation (Figure 5H–K), suggesting a non-canonical modulation of STAT5 function. Thus, while our data do not support extensive global rewiring, they provide robust evidence for a focused mechanism where Glud1 inhibition maintains HSCs through its specific interplay with the JAK–STAT pathway.

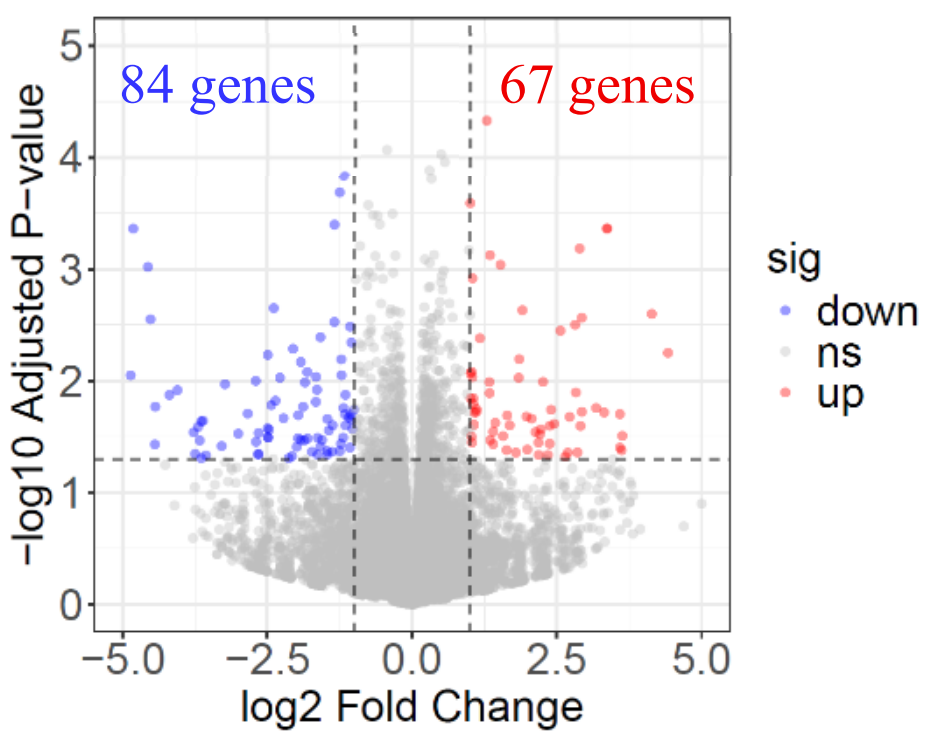

Figure B for reviewers. The deferentially expressed genes between EPCR^{High} HSCs maintained under control conditions and under Glud1 inhibition.
After EPCR^{High} HSCs were cultured for 4 days in the absence (control) or presence of R162, maintained EPCR^{High} HSCs were isolated and subjected to RNA-Seq. Red or blue dots represent genes in up-regulated (Red) or down-regulated (Blue) by R162 treatment. Threshold was set at fold change >2 and p<0.05.

Upregulated in Control

NAME	ES	NES	NOM p-val	FDR q-val	FWER p-val
HALLMARK_E2F_TARGETS	-0.59067327	-1.7118734	0.06613226	0.18735519	0.097
HALLMARK_G2M_CHECKPOINT	-0.50744724	-1.619972	0.055214725	0.18148905	0.206
HALLMARK_MYC_TARGETS_V1	-0.5087321	-1.3966365	0.17319588	0.5375115	0.582
HALLMARK_ESTROGEN_RESPONSE_LATE	-0.299838	-1.3646148	0.05367793	0.5816887	0.763
HALLMARK_DNA_REPAIR	-0.38664016	-1.3284843	0.2237354	0.57804114	0.803

Upregulated in R162

NAME	ES	NES	NOM p-val	FDR q-val	FWER p-val
HALLMARK_ANGIOGENESIS	0.39134264	1.4030622	0.08695652	1	0.589
HALLMARK_HEDGEHOG_SIGNALING	0.42746538	1.3402042	0.16465864	0.99755436	0.795
HALLMARK_BILE_ACID_METABOLISM	0.24772178	1.0791502	0.29282868	1	1
HALLMARK_IL6_JAK_STAT3_SIGNALING	0.26729158	1.053857	0.38854805	1	1
HALLMARK_ALLOGRAFT_REJECTION	0.23062278	1.0503715	0.30942622	1	1

ES: Enrichment Score

NES: Normalized enrichment Score

Table B for reviewers. The result of gene set enrichment analysis.
After EPCR^{High} HSCs were cultured for 4 days in the absence (control) or presence of R162, maintained EPCR^{High} HSCs were sorted and subjected to RNA-Seq followed by gene set enrichment analysis. Tables showed that top5 showing highest normalized enrichment score (NES) in each condition.

• No experiments investigate whether changes in αKG availability affect epigenetic dioxygenases such as TET or JmjC, leaving a major conceptual gap in linking amino acid metabolism to chromatin remodeling.

We agree with the reviewer that αKG-dependent epigenetic dioxygenases, such as members of the TET and JmjC families, represent a critical mechanistic link between amino acid metabolism and chromatin regulation. However, as demonstrated in our study, restoring global αKG levels through dimethyl-αKG (dmαKG) supplementation failed to abolish or significantly alter the positive effect of R162 on HSC maintenance (Figure S4A and C–G). Therefore, our experimental evidence strongly suggests that changes in global αKG availability are not the primary causal factor driving the effect of Glud1 inhibition on HSC maintenance. While we cannot entirely exclude the possibility that subtle or locus-specific epigenetic regulation is mediated by these dioxygenases—potentially through localized metabolic pools rather than global abundance—our data indicate that global αKG-mediated chromatin remodeling is not the primary determinant in this context. Accordingly, we have expanded our discussion to address this conceptual framework and specify the possibility of localized epigenetic regulation in the revised manuscript (Page 16, Line 376–399).

• Epigenetic outputs—including alterations in 5hmC or histone methylation—are not assessed, despite their central relevance to how metabolic perturbations shape stem-cell identity.

We agree that epigenetic outputs, including DNA and histone modifications such as 5-

hydroxymethylcytosine (5hmC) and histone methylation, represent a central mechanism through which metabolic perturbations influence stem cell identity. However, our experimental data strongly indicate that changes in global αKG availability are not the primary causal factor driving HSC maintenance upon Glud1 inhibition. In light of this, we believe that αKG-dependent epigenetic regulation is unlikely to be the primary determinant of the effects observed in our study. To further substantiate this, we confirmed that R162 treatment does not significantly alter the global levels of DNA and histone methylation (see Response Figure C below). While we acknowledge that the possibility of subtle or locus-specific changes in chromatin or DNA methylation cannot be entirely excluded—and may indeed contribute to R162-mediated HSC maintenance—we suggest that such global epigenetic remodeling is not the driving force. Accordingly, we have specified this conceptual scope and highlighted the necessity for future investigations into locus-specific epigenetic regulation in Discussion section of the revised version (Page 16, Line 376–399).

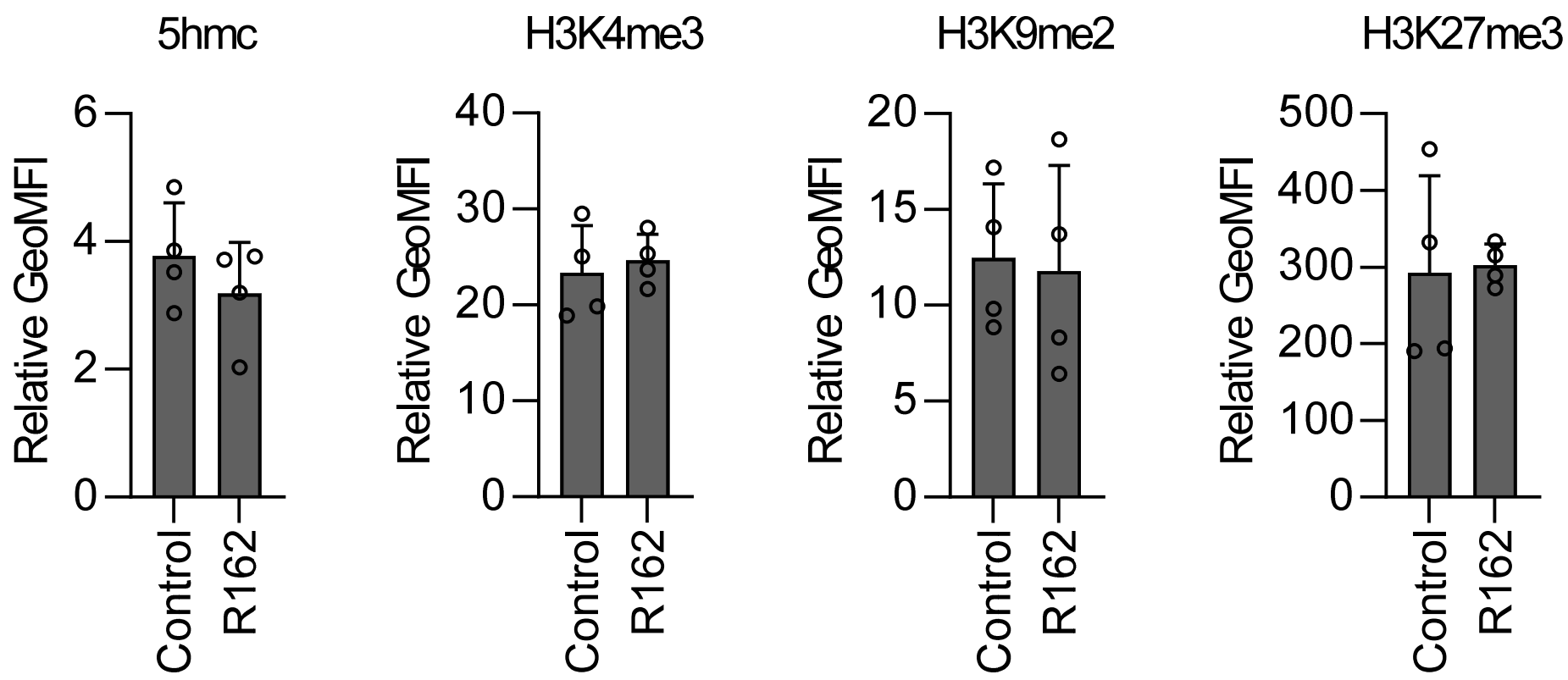

Response Figure C. The effect of R162 on DNA or histone methylation in HSCs.
After HSCs were cultured for 1 day in the absence or presence of R162, the levels of 5hmC, H3K4me3 H3K9me2 and H3K27me3 were examined.

- **Although multimode and ATAC-seq data are presented, these are not integrated with metabolic or proteomic findings, resulting in a fragmented mechanistic narrative that does not convincingly connect the three layers.**

We agree that integrating metabolic, proteomic, and epigenetic layers is essential for a comprehensive mechanistic interpretation. However, as noted in our previous responses and based on our experimental evidence (Figure S4), we found that global metabolic alterations—specifically changes in αKG levels and the NAD^+/NADH ratio—are independent of the Glud1 inhibition-mediated effect on HSC maintenance.

Instead, through refined analysis, our integrated scMultiome data indicated that the molecular framework supporting HSC identity relies on a specific synergy between the regulation of Glud1 catabolic activity and the active JAK–STAT signaling axis at both the transcriptional and epigenetic

levels (Figures 4 and S5; Page 9, line 222–Page 11, line 281). We bridged the gap between these layers by demonstrating that Glud1 physically interacts with STAT5a at the protein level. Glud1 inhibition disrupts this association without affecting STAT5 phosphorylation, indicating a phosphorylation-independent regulatory mechanism (Figure 5I–K). We further validated this integration by showing that the functional benefits of Glud1 inhibition are dependent on robust JAK–STAT signaling (Figures 5A–F). Collectively, these findings provide a coherent narrative linking Glud1 catabolic activity to the regulation of gene expression and chromatin accessibility under robust JAK–STAT signaling, characterizing a specific HSC 'state' that supports self-renewal.

However, the precise molecular mechanism by which the Glud1–STAT5a association regulates STAT5 function remains to be fully elucidated. As suggested, we have explicitly stated the potential nature of the Glud1–STAT5a association and highlighted the necessity for further multifaceted investigations in the revised Discussion (Page 16, line 399–Page 17, line 402). Moreover, we have tempered our interpretation throughout the manuscript, characterizing this interaction as a correlative cellular state associated with self-renewing divisions rather than a definitive mechanistic driver (e.g. Page 2, line 62–65; Page 4, line 110–113; Page 13, line 306–309; Page 18, line 434–436).

• The reported chromatin accessibility differences are modest and not attributed to defined metabolic or signaling inputs, weakening the argument that Glud1 modulates chromatin structure in a functionally meaningful manner.

We acknowledge the reviewer's concern regarding the subtle chromatin accessibility differences observed in our initial analysis. To address this and define the HSC fraction more objectively, we focused our analysis on the five clusters (0, 1, 5, 10, and 13) showing the highest enrichment of *Procr* (EPCR)$^{+}$ *Cd48*$^{-}$ cells (Figures 4A–D and S5A–D). This refined HSC population was then subjected to sub-clustering and trajectory analysis, which identified two distinct differentiation pathways originating from re-cluster 0 (Trajectory 1: 0→2→3; Trajectory 2: 0→1→4) (Figure 4E–H). Notably, R162 treatment significantly increased the cell counts in the most primitive re-cluster 0 while decreasing the population in re-cluster 3 (Figure 4J), indicating that Glud1 inhibition suppresses the 0→2→3 differentiation trajectory. Importantly, while re-clusters 0, 2, and 3 shared a common enrichment of STAT family motifs within their open chromatin regions, they exhibited distinct gene expression patterns, chromatin accessibility profiles, and differential enrichment of other transcription factor motifs (Figures 4K and S5M–N). These findings suggest that Glud1 inhibition maintains the primitive HSC state by suppressing a specific differentiation trajectory characterized by defined chromatin structural changes and gene expression patterns. We believe that this refined definition of the HSC fraction clarifies the functional significance of Glud1 inhibition in regulating and maintaining the epigenetic landscape of HSCs.

Accordingly, we have incorporated new data (Figures 4D, S5A–D and N), replaced previous data

with these refined data (Figures 4E–H, J, K and S5M) and updated the corresponding descriptions (Page 9, line 222–Page 10, line 253 and Page 25, line 595–606).

- **The pseudo time trajectory analysis appears reliant on predefined phenotypic markers rather than unbiased metabolic or proteomic features, raising the possibility of circular reasoning in defining the "primitive" HSC state.**

We agree that defining trajectory origins based solely on predefined phenotypic markers could raise concerns regarding circular reasoning. Consequently, in the revised version, we introduced an unbiased computational approach using CytoTRACE2 to identify root cells within the pseudotime analysis performed by Monocle3. CytoTRACE2 independently identified the cells with the top five highest root cell scores exclusively within re-cluster 0 (Figure 4E and F). Furthermore, re-cluster 0 exhibited the highest module scores for several established HSC-related gene sets compared to all other re-clusters (Figure 4H). These results demonstrate that the objective definition of root cells in our pseudotime analysis is consistently supported by both unbiased stemness prediction algorithms and global transcriptomic profiles.

Accordingly, in the revised version, we have replaced the previous data with these new data (Figure 4F and H) and updated corresponding explanations (Page 10, line 237–242 and Page 25, line 597–598). We thank the reviewer for this constructive advice, which has significantly enhanced the objectivity and rigor of our trajectory analysis.

- **The use of extremely low-glutamine culture conditions likely imposes non-physiological metabolic stress, yet the manuscript does not examine how such artificial constraints themselves affect amino-acid handling, proteome stability, or HSC state independent of Glud1.**

We agree that extremely low-Gln culture conditions may impose metabolic constraints. In our study, we already disentangled the effects of low-Gln conditions by showing that they suppress the proliferative activity of HSCs, evidenced by a significant decrease in total cell numbers after 4 days of culture (Figure 2L). Importantly, while the Glud1 inhibitor R162 effectively promoted HSC maintenance (Figures 3C–E), it did not negatively impact total cell numbers (Figure 3F). Conversely, the suppressive effect of low-Gln on proliferation was recapitulated by treatment with aminooxyacetic acid (AOA), an inhibitor of aminotransferases—the other primary enzymatic pathway for Glu catabolism (Figure 3F). These data indicate that the inhibition of proliferation under low-Gln conditions is primarily attributed to aminotransferase inhibition rather than Glud1 inhibition. Mechanistically, both low-Gln conditions and AOA treatment significantly decreased intracellular levels of aspartate (Asp) (Figures S3A–C). Given that HSCs express extremely low levels of Asp transporters and rely heavily on de novo Asp synthesis via aminotransferase reactions for hematopoietic regeneration (Qi et al., *Cell Stem Cell*, 2021, 28(11): 1982-1999 e1988), our findings

suggest that the suppression of HSC proliferation under low-Gln is a consequence of attenuated Asp synthesis.

Therefore, we conclude that low-Gln conditions exert two distinct metabolic effects: (1) a suppressive effect on proliferative activity caused by impaired aminotransferase activity and Asp depletion, and (2) a specific, positive effect on stem cell maintenance attributed to Glud1 inhibition. These explanations have been already described to the Results section (Page 7, line 176–Page 8, line 195)."

- **The manuscript currently depends too heavily on transcriptional signatures, and would benefit from a more integrated mechanistic narrative connecting metabolism, protein-level signaling, and epigenetic regulation.**

We agree that integrating metabolic, protein-level signaling, and epigenetic regulation is essential for a more robust mechanistic interpretation. As noted in our previous responses, our experimental data indicate that changes in global αKG abundance or the NAD^+/NADH ratio are not the primary drivers of Glud1-inhibition-mediated HSC maintenance.

Instead, through refined analysis, our integrated scMultiome indicated that the molecular framework supporting HSC identity relies on a specific synergy between Glud1 catabolic activity and the active JAK–STAT signaling axis at both the transcriptional and epigenetic levels (Figures 4 and S5). Specifically, JAK-dependent genes were differentially expressed across re-clusters 0, 2, and 3 (Figure 4L), despite these clusters sharing a similarly high enrichment of STAT-related binding motifs (Figure 4K). Moreover, these re-clusters showed distinct module scores for HSC/differentiation signatures 2 (Figure 4H). Furthermore, in addition to these JAK-dependent genes, these signature gene sets for HSCs (e.g., EPCR High and MolO) and differentiation (e.g., EPCR Low and NoMO) showed a significant, approximately 2-fold enrichment of genes with high expression–accessibility correlation ($r > 0.5$) compared to the genome-wide average (Figures 4M and S5Q). Importantly, this enrichment pattern remained robust even when the analysis was restricted to potential STAT5-binding regions (Figures 4M and S5R). Notably, Glud1 inhibition suppressed a specific differentiation trajectory (re-clusters 0→2→3) characterized by distinct module scores for these HSC/differentiation signatures and high STAT motif accessibility (Figures 4E–H, J and K). Based on these observations, we hypothesized that the beneficial effects of R162 on HSC self-renewal are functionally dependent on robust JAK–STAT activity. We validated this connection by demonstrating that pharmacological inhibition of JAK2 significantly attenuated the effects of R162 on HSC maintenance (Figures 5A–F). At the protein level, we confirmed that Glud1 physically interacts with STAT5a in HSCs. This association is disrupted by Glud1 inhibition or low-glutamine (Gln) conditions without altering STAT5 phosphorylation levels. Collectively, these findings exhibited a distinct HSC state in which the maintenance of self-renewal capacity under Glud1 inhibition is characterized by robust JAK–STAT activity and a catabolism-

dependent modulation of the Glud1-STAT5a association.

Accordingly, we have incorporated new data (Figures 4D, L, M and S5A–D and N–R), replaced previous data with these refined data (Figures 4E–H, J, K and S5M) and updated the corresponding descriptions (Page 9, line 222–Page 11, line 281). While the precise molecular details of how this association regulates STAT5 function remain to be fully elucidated, we believe our integrated dataset provides a compelling narrative linking metabolic state to signaling outputs. As suggested, we have clarified the potential nature of this interpretation and highlighted the need for future studies (Page 16, line 399–Page 17, line 402). Additionally, we have tempered our claims throughout the manuscript, characterizing the Glud1–STAT5a association as a correlative cellular state associated with self-renewal rather than a definitive mechanistic driver (e.g. Page 2, line 62–65; Page 4, line 110–113; Page 13, line 306–309; Page 18, line 434–436).

- **Overall, although the conceptual hypothesis is intellectually appealing, the lack of depth across metabolic, proteomic, and epigenetic dimensions prevents the authors from presenting a fully substantiated biological mechanism at this stage.**

We thank the reviewer for their interest in our model and for providing such constructive feedback. As discussed in our previous responses, although R162 induces changes in global αKG abundance and the NAD^+/NADH ratio, our experimental evidence demonstrates that these global metabolic shifts are not the primary drivers of the effects of Glud1 inhibition on HSC maintenance (Figure S4). Consequently, rather than focusing on global metabolism, we identified the interplay between Glud1 and JAK–STAT signaling as a critical regulatory axis. Refined analysis of our integrated scMultiome data indicates that the molecular framework supporting HSC identity relies on a specific synergy between Glud1 catabolic activity and the active JAK–STAT signaling axis at both the transcriptional and epigenetic levels (Figures 4 and S5). We established a mechanistic link by demonstrating that HSC maintenance under Glud1 inhibition is strictly dependent on robust JAK–STAT signaling (Figures 5A–F). Furthermore, we confirmed that Glud1 physically interacts with STAT5a in HSCs. Crucially, while Glud1 inhibition or low-glutamine (Gln) conditions disrupt this interaction, they do so without altering STAT5 phosphorylation levels. These findings characterize a distinct HSC state where the maintenance of self-renewal capacity is associated with both robust JAK–STAT activity and a catabolism-dependent Glud1–STAT5a association pattern.

Accordingly, we have incorporated new data (Figures 4D, L, M and S5A–D and N–R), replaced previous data with these refined data (Figures 4E–H, J, K and S5M) and updated the corresponding descriptions (Page 9, line 222–Page 11, line 281). While our study provides multi-layered evidence bridging metabolism, protein interaction, and the epigenetic landscape, we acknowledge that the precise molecular details of how the Glud1–STAT5a association modulates STAT5 function remain to be fully elucidated. As suggested, we have clarified this in the revised Discussion by addressing the

potential nature of this interpretation (Page 16, line 399–Page 17, line 402). Moreover, we have tempered our claims throughout the manuscript, characterizing the association as a correlative cellular state associated with self-renewing divisions rather than a definitive mechanistic driver (e.g. Page 2, line 62–65; Page 4, line 110–113; Page 13, line 306–309; Page 18, line 434–436).

Reviewer #2 (Remarks to the Author)

Despite multiple efforts and success to optimize in vitro culture conditions, expansion of hematopoietic stem cells (HSCs) while remaining their stemness capacity still remains a challenge. In this manuscript they discover that suppression of Glud1, a key enzyme for glutamate metabolism, in vitro supports the maintenance and expansion of functional HSCs. The mechanism of Glud1 inhibition on HSC self-renewal maintenance is coupled to JAK-STAT signalling. The observations are interesting and will have a great impact on improving in vitro culture conditions for functional HSCs.

We thank the reviewer for the careful review of our manuscript and for providing constructive and valuable advice. In accordance with the reviewer's suggestions, we have extensively revised our manuscript and believe that the revised version is significantly improved. We appreciate the insightful feedback once again.

- The data in Figure 1 on EPCRhi cells are interesting, however several groups have by now shown EPCR high expressing stem cells to be the most potent one, so the data is not really novel.

We agree with the reviewer that $EPCR^{High}$ HSCs have been established as one of the most primitive HSC populations in the steady state by several groups, including our own. However, there have been few reports demonstrating that the $EPCR^{High}$ phenotype remains a robust and convincing marker of functional HSCs after exposure to conventional *ex vivo* culture conditions. In this study, we confirmed that $EPCR^{High}$ L^-ESLAM HSCs are clearly distinguishable from $EPCR^{Low}$ L^-ESLAM cells *in vivo* as well as *in vitro*, not only in terms of stem cell function but also in their chromatin accessibility profiles.

By identifying these differences within the HSC fraction, we were able to focus on the transformation from the $EPCR^{High}$ to the $EPCR^{Low}$ state as one of the earliest steps in HSC differentiation. This approach was instrumental in identifying the key factors required for successful *ex vivo* HSC expansion. Therefore, we believe that the detailed comparison between the $EPCR^{High}$ and $EPCR^{Low}$ fractions, particularly in the *in vitro* context, is essential for the narrative of this manuscript. To acknowledge the existing literature as suggested, we have cited several seminal papers regarding the primitivity of $EPCR^{High}$ HSCs in Results section of the revised version (Page 5, Line 126).

- Figure 2B, 5FU data: only absolute numbers of EPCRhi HSCs are shown over time. However, it would be informative to also show these analysis for all opther HSC populations for which the Glu and EdC analysis are shown in figure 2D and 2E

We agree that providing absolute numbers for all HSC sub-populations, in addition to the $EPCR^{High}$ fraction, is valuable for understanding the recovery process of the HSC pool following 5-

FU administration. Accordingly, we have included the absolute cell numbers of all HSCs and non-EPCR^{High} HSCs (defined as L^{-}ESLAM HSCs excluding the EPCR^{High} fraction) and the gate strategy for all HSCs in the revised manuscript (Figures 2B, 2D, and S2) along with a corresponding explanation in Results sections (Page: 6, Line 161–162). Interestingly, within the total HSC pool, only the EPCR^{High} fraction exhibited a significant increase by day 7 post-5-FU administration (Figures 2B–D). These data strongly support the notion that EPCR^{High} HSCs possess a significantly higher potential for self-renewing divisions compared to other HSC sub-populations. We thank the reviewer for this constructive suggestion, which has helped clarify the unique functional potential of the EPCR^{High} population during regeneration.

- Figure 3B-F: Glud1 inhibition results in elevated frequency of EPCRhi HSCs without reducing total cell numbers: what happens to the composition of the cultures in the presence of Glud1 inhibition regarding progenitor cell numbers, differentiation?

We agree that analyzing the impact of Glud1 inhibition on progenitor cells is critical for a comprehensive understanding of how HSC stemness is maintained. In contrast to the observed increase in EPCR^{High} HSCs, Glud1 inhibition significantly decreased both the frequency and absolute number of EPCR$^{Low/-}$ cells (Figure 3C–E and 3G–H).

Given that Glud1 inhibition did not alter the total cell number at the end of the culture period, these data demonstrate that Glud1 inhibition maintains the HSC pool by suppressing differentiation into EPCR$^{Low/-}$ progenitor cells, rather than by simply increasing overall cell proliferation. We have added the gate strategy for EPCR$^{Low/-}$ cells into Figure 3C, incorporated the data regarding EPCR$^{Low/-}$ cells (Figure 3E and H) and updated the corresponding interpretations to the revised manuscript to clarify the effect of Glud1 inhibition on the overall culture composition (Revised Result, Page: 8, Line 195–199). We thank the reviewer for this insightful comment, which has helped us better define the mechanism of HSC expansion in our system.

- Figure 4: both re-clusters 1 and 2 show significant enrichment of JAK-STAT signaling, however for cluster 1 this results in HSC maintenance and for cluster 2 a reduction in numbers. How do the authors explain these differences, when JAK-STAT signaling should be leading here?

We agree that clarification was needed regarding the divergent phenotypes observed in populations with similarly robust STAT activity. In the revised manuscript, we re-analyzed our scMultiome data by using objectively refined criteria to define the HSC fraction (Figures 4A-D and S5A-D). As a result, the revised re-clusters 0, 2, and 3 now correspond to the original re-clusters 1 and 2; all three exhibit high STAT motif enrichment compared to other clusters (Figure 4E and K). Critically, we found that most JAK-dependent genes were differentially expressed across re-clusters 0, 2, and 3 (Figure 4L), despite these clusters sharing similar STAT motif accessibility. These three re-

clusters constitute a specific differentiation trajectory (0→2→3) characterized by distinct module scores for HSC/differentiation signatures (Figures 4E–H). Furthermore, in addition to these JAK-dependent genes, signature gene sets for HSCs (e.g., EPCR-High and MolO) and differentiation (e.g., EPCR-Low and NoMO) showed a significant, approximately 2-fold enrichment of genes with high expression–accessibility correlation ($r > 0.5$) compared to the genome-wide average (Figure S5Q). Importantly, this enrichment pattern remained robust even when the analysis was restricted to potential STAT5-binding regions (Figure S5R). Notably, Glud1 inhibition suppressed this specific differentiation trajectory (re-clusters 0→2→3) characterized by distinct module scores for these HSC/differentiation signatures and high STAT motif accessibility (Figures 4E–H, J and K). These findings suggest that the molecular framework supporting HSC identity relies on a specific synergy between Glud1 catabolic activity and the active JAK–STAT signaling axis. We functionally validated that HSC maintenance under Glud1 inhibition is strictly dependent on robust JAK–STAT signaling using pharmacological inhibitors (Figures 5A–F). Furthermore, we demonstrated that Glud1 physically interacts with STAT5a and that inhibiting Glud1 activity disrupts this association without affecting STAT5 phosphorylation levels (Figures 5G–K). Collectively, these findings characterize a distinct HSC state in which the maintenance of self-renewal capacity is associated with robust JAK–STAT activity and a catabolism-dependent Glud1–STAT5a association pattern.

Accordingly, we have incorporated new data (Figures 4D, L, M and S5A–D and N–R), replaced previous data with these refined data (Figures 4E–H, J, K and S5M) and updated the corresponding descriptions (Page 9, line 222–Page 11, line 281). While the precise molecular mechanism remains to be fully defined, we suggest that the physical association with Glud1 modulates the functional status or target specificity of STAT5 to balance HSC self-renewal and differentiation. Accordingly, we have explicitly addressed this interpretation and the need for future studies in the Discussion section (Page 16, line 399–Page 17, line 402).

- Figure 5: JAK inhibitors alone already result in reduced total numbers (Figure 5E) and a reduction in the fold increase in EPCRhi HSCs (Figure 5F): thus, the effect of inhibiting JAK/STAT signalling alone is already dominant. The combined treatment showing the same as JAK inhibition alone can thus solely be the result of JAK inhibition, and not necessarily show that Glud1 effects are via JAK/STAT.

We appreciate the reviewer's insightful comment regarding the potential confounding effect of the overall reduction in cell numbers upon JAK inhibition. To clarify whether the loss of the Glud1-inhibition effect was a specific consequence of JAK–STAT suppression or merely a bystander effect of overall growth inhibition, we examined the impact of R162 in the presence of SCH772984, an Erk1/2 inhibitor.

Unlike JAK inhibitors, SCH772984 significantly reduces total cell numbers by suppressing

proliferative activity without affecting STAT5 phosphorylation levels. Notably, we found that Glud1 inhibition by R162 continued to exert its positive effects on both HSC frequency and absolute numbers, even when total cell proliferation was significantly impaired by Erk1/2 inhibition (Figure S6). These results demonstrate that the beneficial effects of Glud1 inhibition are not simply dependent on cell density or general proliferative state, but specifically require an active JAK–STAT signaling axis. Accordingly, we have incorporated these data as Figure S6 in the revised manuscript and updated the corresponding interpretations in Results section (Page 12, Line 287–292).

- The ip and WB in figure 5G is not convincing. There might be a very weak band…

We agree that the STAT5 band in the original immunoprecipitation (IP) data was faint. To more robustly validate the physical interaction between Glud1 and STAT5, we performed the IP in the reciprocal direction using an anti-STAT5 antibody, followed by Western blotting (WB) with an anti-Glud1 antibody.

This approach yielded a significantly clearer and stronger Glud1 band in the STAT5-immunoprecipitated samples (Figure 5G). Accordingly, we have replaced the original IP-WB data (IP: Glud1, WB: STAT5) with these improved results (IP: STAT5, WB: Glud1) in Figure 5G and updated the corresponding text in the Material & Method section (Page 25, Line 613–Page 26, Line 622). We believe this revised data provides more convincing evidence of the physical association between Glud1 and STAT5.

- It is too bad that the authors are not able to perform R162 treatment in the PVA cultures. Did they check the Glu levels in PVA cultures? How are they compared to the levels in their conventional cultures?

We agree that understanding the metabolic state within the F12-PVA culture system is important for contextualizing our findings. To address the reviewer's question, we compared the intracellular glutamate (Glu) levels in HSCs cultured under conventional conditions versus those in F12-PVA conditions. Our analysis confirmed that intracellular Glu levels are comparable between the two culture systems (see Response Figure D below). This evidence suggests that the mechanism by which Glud1 inhibition maintains HSCs is distinct from the mechanisms underlying the F12-PVA system. As noted, we have already provided a explanation regarding the distinct mechanistic differences between Glud1 inhibition and the PVA-based system in Discussion section (Page 15, Line 369–370).

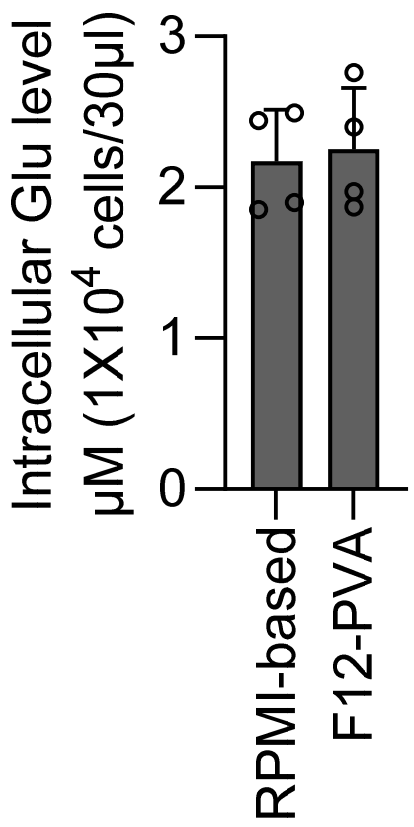

Response Figure D. The effect of F12-PVA system on intracellular Glu level.
After HSCs were cultured for 1-day under the RPMI-based conventional or the F12-PVA conditions, intracellular Glu level in HSCs were examined.

- The longterm cultures in Figure 7 are interesting. Is there a change that one starts to select for specific subclones and thus is looking a clonal expansion?

We agree that the clonality and potential for clonal expansion of HSCs during long-term culture under Glud1 inhibition are important considerations. While Glud1 inhibition significantly reduced the proportion of differentiated progenitors (e.g., EPCR$^{Low/-}$ cells), differentiation still occurred within the culture (Figure 3). Moreover, even EPCR^{High} HSCs maintained in the presence of R162 continued to produce differentiated progeny during subsequent serial passages (Figure 7), suggesting that the cultures do not consist of a single non-differentiating "runaway" clone. However, our data indicate that the beneficial effects of Glud1 inhibition strictly require robust JAK–STAT signaling, and our scMulti-omic analysis revealed functional heterogeneity in STAT activity among HSCs even under identical culture conditions. Therefore, Glud1 inhibition may provide a selective advantage to a subset of HSCs capable of sustaining robust JAK–STAT signaling throughout the culture period. We have added a discussion of this functional heterogeneity and the possibility of selecting HSCs with high JAK–STAT signaling capacity (Revised Discussion, Page 18, Line 440–442).

Expansion of mouse HSCs is interesting however the main clinical interest is with human HSCs. Therefore the impact of the story would significantly increase if human cultures were tested for Glu levels and inhibition of Glud1 would be tested in these human cultures.

[Redacted]

[Redacted]

[Redacted]

[Redacted]

Reviewer #3 (Remarks to the Author):

1) Summary and overall assessment

In this manuscript, Hashimoto and colleagues identify the glutamate dehydrogenase (Glud1) inhibitor R162 as an efficient strategy to enhance self-renewal divisions while preserving a bona fide HSC phenotype, both transcriptionally and functionally. The major impact of the work lies in the identification of a novel compound and metabolic strategy that enables robust expansion of murine phenotypic HSCs ex vivo.

The central claims of the study are, in my view, well supported by the data presented:

Low-glutamine culture conditions preserve HSC identity ex vivo but do not efficiently drive expansion.

Genetic (sgGlud1) or pharmacological (R162) inhibition of Glud1 leads to a marked increase in phenotypic and functional HSCs, accompanied by true expansion.

STAT5 appears to physically and functionally interact with Glud1, supporting a metabolic–transcriptional coupling mechanism that is well aligned with existing literature.

Combining R162 with PVA-F12 culture conditions further enhances LT-HSC expansion, likely defining a new best-in-class medium composition for the ex vivo expansion of multipotent HSCs.

This last point alone makes it highly likely that the manuscript will be widely used and cited by the stem cell community.

We sincerely thank the reviewer for the careful and insightful review of our manuscript. We are deeply gratified by the positive assessment of our findings, particularly the recognition of Glud1 inhibition as a novel strategy for *ex vivo* HSC expansion. In accordance with the reviewer's constructive suggestions, we have extensively revised the manuscript, and we believe these improvements have significantly enhanced its quality and clarity. We appreciate the reviewer's encouraging feedback once again.

2) Major comments

Clarity regarding culture media

Throughout the manuscript, several distinct basal media are used in R162 experiments (RPMI-based versus F12-PVA-based formulations). It would greatly improve clarity if each figure (or at least each experiment) explicitly stated which basal medium was used. This is particularly important given that media composition clearly modulates the magnitude of the observed effects.

We agree with the reviewer that clearly identifying the basal medium used in each experiment is essential, as the media composition significantly influences the magnitude of the observed effects. In accordance with this suggestion, we have explicitly specified the basal medium used for each experiment in the revised figures and/or figure legends (e.g., RPMI-based, F12-PVA-based etc.). We appreciate this constructive comment, which has improved the clarity and transparency of our study.

DMSO controls

I did not find any explicit mention of DMSO being used as a vehicle control, nor the final DMSO concentration employed. Given that R162 is dissolved in DMSO, this information is important to report clearly in the Methods.

This point is not merely technical: in our own hands (unpublished observations), 0.1% DMSO causes mild but reproducible effects on LT-HSC expansion. Interestingly, this aligns with recent reports describing radioprotective effects of DMSO mediated through LT-HSCs (e.g. Free Radic Biol Med 2020; DOI: 10.1016/j.freeradbiomed.2020.03.021). I am confident the appropriate controls were performed, but explicitly stating the vehicle control and DMSO percentage would strengthen rigor and reproducibility.

We agree with the reviewer that explicitly stating the vehicle control and its final concentration is essential for ensuring experimental rigor and reproducibility. As the reviewer correctly anticipated, we used 0.1% DMSO as the vehicle control in all experiments involving R162, as the R162 stock solution (dissolved in DMSO) was diluted 1:1,000 for the HSC cultures. In response to this constructive advice, we have updated the Materials and Methods section to clearly state the solvent and the specific vehicle control conditions used (Page 22, Line 538–Page 23, Line 548). We thank the reviewer for highlighting the potential biological effects of DMSO, and we believe this addition strengthens the technical clarity of our manuscript.

Extension to human HSCs

The effects of R162 on murine HSC expansion are sufficiently strong that it is difficult not to wonder whether similar outcomes would be observed in human HSC cultures. Even a limited set of exploratory experiments using human cells—well within the technical scope of this excellent group—would substantially enhance the impact and translational relevance of the study.

[Redacted]

[Redacted]

NAD$^+$ metabolism and NR supplementation

The authors show that R162 treatment or sgGlud1 increases NAD$^+$ levels in HSCs, yet supplementation with nicotinamide riboside (NR) does not phenocopy these effects. It would be helpful to know whether the NR treatment used here actually increases intracellular NAD$^+$ levels in HSCs. If this is already established in the literature, citing it would help; otherwise, directly measuring NAD$^+$ following NR treatment would clarify interpretation.

We agree with the reviewer that confirming whether nicotinamide riboside (NR) treatment effectively modulates intracellular NAD$^+$ levels is essential for interpreting its impact on HSCs. As the reviewer anticipated, our measurements confirmed that NR treatment significantly increased the NAD$^+$/NADH ratio in HSCs, comparable to the levels observed with R162 treatment (Figure S4B). Despite this increase in the NAD$^+$/NADH ratio, NR treatment failed to promote HSC expansion. These results strongly suggest that an increase in the NAD$^+$/NADH ratio is not the primary factor responsible for the positive effects of Glud1 inhibition on HSC maintenance. Accordingly, we have incorporated the new data into Figure S4B and updated the corresponding explanation in Results section (Page 9, Line 214-219).

In contrast to NR, we found that nicotinamide mononucleotide (NMN) treatment had a minimal impact on the NAD$^+$/NADH ratio under our culture conditions. Consequently, we have removed the data regarding NMN from the revised manuscript to maintain focus on the more conclusive results obtained with NR. We thank the reviewer for this constructive comment, which has helped clarify the metabolic requirements for HSC expansion.

Single-cell analysis of R162-treated HSCs

R162-treated HSCs clearly undergo a substantial shift in cell state. While the clustering and enrichment analyses are informative, several aspects could be strengthened:

Replicates: There is no explicit mention of biological replicates for the single-cell experiments.

If replicates were not included, conclusions drawn from these analyses should be appropriately tempered.

We agree with the reviewer that explicitly stating the presence or absence of biological replicates is essential for the transparent interpretation of single-cell data. As the reviewer noted, our scMulti-omic analysis was performed without biological replicates. In response to this limitation, we have carefully tempered our language throughout the revised manuscript, reframing the findings from these analyses as suggestive or hypothesis-generating rather than definitive conclusions. Importantly, we have ensured that the key insights derived from the single-cell data—such as the role of JAK–STAT signaling—were independently validated through multiple functional and protein-level experiments (e.g., Figure 5). We thank the reviewer for this constructive advice, which has improved the rigor and balance of our manuscript.

HSC subsetting: When defining HSC clusters, it is unclear why cluster 5 was excluded. This cluster appears Cd48^low and expresses Procr, and supplementary figures show expression of established HSC markers such as Mecom, Mllt3, Itsn1, and Mir99ahg. The high expression of Top2a and Mki67 suggests these may represent cycling HSCs. Applying cell-cycle correction or explicitly discussing this population would strengthen the analysis.

We agree with the reviewer that a more explicit and objective definition of the HSC fraction is necessary for the single-cell analysis. In the revised version, we have re-defined the HSC fraction by selecting the five clusters (Clusters 0, 1, 5, 10, and 13) that contain the highest frequency of *Procr* (EPCR)$^{+}$ *Cd48*$^{-}$ cells (Figures 4D and S5D). As the reviewer astutely noted, Cluster 5 was selected as part of the HSC fraction; we believe its inclusion provides a more comprehensive and unbiased view of the HSC compartment. Accordingly, we have incorporated these new data (Figures 4D and S5D) and updated the corresponding explanations into the Results section of the revised version (Page 9, Line 227-231).

Importantly, our subsequent analyses using this objectively re-defined HSC population yielded results consistent with our previous findings, confirming that the core of our conclusions remains robust. We believe this refined selection method significantly strengthens the rigor of our single-cell analysis. We thank the reviewer for this insightful recommendation.

STAT-related programs: Despite the proposed STAT5–Glud1 interaction, there is no focused analysis of STAT target gene expression or chromatin accessibility. Given that scMultiome data were generated, a deeper interrogation of differentially accessible regions and transcription factor motifs would be highly valuable. This dataset is particularly important, as high-quality scMultiome data from HSC cultures remain relatively scarce.

To address this, we first defined a set of 'JAK-dependent genes' by comparing the gene expression

profiles of HSCs cultured with or without ruxolitinib (Ruxo), a JAK inhibitor (as described in Williams et al, *Blood Adv* (2025) 9(2):291–309; threshold: fold change > 2, $p < 0.05$). This gene set successfully captured representative STAT5 targets, such as *Socs2* and *Cish*. Consistent with our motif enrichment analysis (Figure 4K), we observed that re-clusters 0, 2, and 3—which exhibit high enrichment of STAT-related motifs—also showed significantly higher expression of these JAK-dependent genes compared to re-clusters 1 and 4 (Figure 4L). Interestingly, even among these re-clusters with robust STAT activity, these JAK-dependent genes exhibited differential expression levels (Figure 4L). Furthermore, approximately half of the genes in this set exhibited a high correlation coefficient ($r > 0.5$) between transcript expression and chromatin accessibility (Figures 4M and S5O). In addition to these JAK-dependent genes, signature gene sets for HSCs (e.g., EPCR High and MolO) and differentiation (e.g., EPCR Low and NoMO) showed a significant, approximately 2-fold enrichment of genes with high expression–accessibility correlation ($r > 0.5$) compared to the genome-wide average (Figure S5Q). Importantly, this enrichment pattern remained robust even when the analysis was restricted to potential STAT5-binding regions (Figures 4M, S5P, and S5R). Notably, Glud1 inhibition suppressed a specific differentiation trajectory (re-clusters 0→2→3) characterized by distinct module scores for these HSC/differentiation signatures and high STAT motif accessibility (Figures 4E–H, J and K). These findings suggest that the molecular framework supporting HSC identity relies on the synergy between Glud1-mediated regulation and the active JAK–STAT signaling axis.

Accordingly, we have incorporated these new analyses into Figures 4L–M and S5O–R, and updated the corresponding explanations in the revised manuscript (Page 10, line 256–Page 11, lines 281 and Page 25, lines 595–606). We thank the reviewer for this insightful suggestion, which has significantly enhanced the mechanistic depth of our study.

Non-HSC populations: The remaining clusters appear to represent various activated HSC/MPP states and megakaryocyte-biased progenitors. Were any notable changes observed in these compartments upon R162 treatment?

We agree with the reviewer that analyzing non-HSC populations, such as multipotent progenitors (MPPs), provides important context for understanding the impact of R162 on the hematopoietic hierarchy. To address this, we calculated module scores for HSC and MPP1–5 signatures across the entire single-cell dataset, utilizing both our EPCR-High gene set and established signatures (Wilson et al., *Cell Stem Cell*, 2015, 16(6): 712-724; Sommerkamp et al., *Blood*, 2021, 137(23): 3218-3224). We found that R162 treatment reduced the proportion of cells exhibiting high scores for MPP2 (megakaryocyte/erythroid-biased) and MPP3 (myeloid-biased) (Figures S5A and S5E–L). This observation is highly consistent with our refined sub-clustering analysis, which demonstrated a

decrease in more differentiated progenitor states upon Glud1 inhibition (Figures 4E–H and 4J). These data (Figures S5A and S5E–L) and the corresponding explanations have been incorporated into the Results section of the revised manuscript (Page 9, lines 231–233). We thank the reviewer for this constructive suggestion, which has helped clarify the broader impact of Glud1 inhibition on the hematopoietic landscape.

Global Procr^{+} frequency: Across the full dataset, were Procr-expressing clusters enriched in the R162 condition?

As suggested by the reviewer, we confirmed whether R162 treatment led to a global enrichment of *Procr*-expressing cells across the full scMulti-omic dataset. Our analysis revealed that R162 treatment indeed increased the frequency of both *Procr*$^{+}$ cells and *Procr*$^{+}$*Cd48*$^{-}$ cells compared to the control condition (Figure S5B). Notably, this enrichment observed at the transcriptomic level is highly consistent with our flow cytometric findings (Figure 3C-E). We have incorporated this new data as Figure S5B and updated the corresponding explanations in Result Section of the revised manuscript (Page 9, Line 225–227). We thank the reviewer for this suggestion, which further validates the robustness of our single-cell observations.

HSC state signatures: Additional analyses examining R162-induced changes in established HSC signatures (dormancy, cell cycle, lineage bias, etc.) would further solidify the mechanistic conclusions.

We agree with the reviewer that a more detailed signature analysis of each re-cluster is essential for a comprehensive understanding of how Glud1 inhibition maintains and expands HSCs.

First, the identification of re-cluster 0 as the most primitive 'root' population in our unbiased pseudotime analysis was confirmed by its high scores for established HSC signatures, including MolO (Wilson et al., *Cell Stem Cell*, 2015, 16(6): 712-724), vWF-HSCs (Sanjuan-Pla et al., *Nature*, 2013, **502**(7470): 232-236), and the HSC signature (Sommerkamp et al., *Blood*, 2021, 137(23): 3218-3224) (Figure 4H).

Second, focusing on the two differentiation pathways originating from re-cluster 0 (Trajectory 1: 0→2→3; Trajectory 2: 0→1→4) identified by our unbiased pseudotime analysis (Figures 4E–H), we observed higher scores for signatures of pre-MegE (Pronk, C. J., et al. *Cell Stem Cell*, 2007, **1**(4): 428-442) and MkP (Sanjuan-Pla et al., *Nature*, 2013, **502**(7470): 232-236) in re-cluster 1 compared to re-cluster 2 (Figure 4H). This suggests that Glud1 inhibition does not significantly block differentiation into these specific lineages. Conversely, re-cluster 3 showed significantly higher scores for MPP2, MPP3, and MPP4 compared to re-clusters 1 and 4 (Figure 4H). The reduction in cell numbers in re-cluster 3 upon R162 treatment indicates that Glud1 inhibition specifically suppresses the transition from primitive HSCs to these progenitor states (Figure 4J).

Finally, we examined cell-cycle signatures and found that re-clusters 0, 2, and 3 contained a higher proportion of cells in S/G2/M phases compared to re-clusters 1 and 4 (Figure 4I). Given that R162 treatment suppresses differentiation along the 0→2→3 trajectory, these data suggest that Glud1 inhibition maintains HSC identity even under conditions of high proliferative activity. This explains how R162 promotes robust HSC expansion rather than merely preserving dormancy.

We have incorporated these analyses (Figures 4H and 4I) and added the corresponding interpretations to the Results section of the revised manuscript (Page 10, lines 240–242 and Line 244–246). We thank the reviewer for this suggestion, which has significantly strengthened our mechanistic conclusions.

3) Minor comments

The Methods section describing NAD^+ and αKG measurements states "following manufacturer's protocol," but the reagent kits used are not specified.

We appreciate the reviewer's attention to detail. We have now specified the names and manufacturers of the reagent kits used for these measurements in the revised Materials and Methods section.

An input control appears to be missing from the immunoprecipitation experiments.

We agree that an input control is essential for the valid interpretation of the immunoprecipitation results. Accordingly, we have added the input lanes to the representative blots in Figure 5G to demonstrate the presence of target proteins prior to pull-down.

Several antibody fluorochromes are not listed in the Methods.

The information regarding antibody fluorochromes has been added to the updated antibody list in the revised Materials and Methods (Page 19, Line 449–465).

4) Typographical errors

Line 534: "The resulting IP samples was analyzed by SDS-PAGE" → should be were.

Figure 7K: "The frequency of multi-lineage engfarted mice" → should be engrafted.

We thank the reviewer for the careful proofreading. We have corrected these grammatical and spelling errors in the revised manuscript.

Reviewer #1 (Remarks to the Author):

The manuscript has been clearly improved compared to the initial submission, and the authors are to be commended for their careful and constructive response to the reviewers' comments. In particular, the inclusion of additional data and the reorganization of the text have substantially strengthened the reproducibility and overall persuasiveness of the central observation that Glud1 inhibition contributes to the maintenance and expansion of HSC. The authors have also appropriately tempered their interpretation of the Glud1 and STAT5a interaction, reframing it as a correlative association rather than a definitive mechanistic driver, which helps avoid overstatement. Furthermore, the additional validation of the dependence on JAK–STAT signaling provides important functional support and clarifies the biological relevance of the findings.

We are deeply grateful for the reviewer's generous commendation and highly encouraging assessment of our revised manuscript. It is immensely rewarding to hear that our additional data, text reorganization, and the mechanistic validation of JAK–STAT signaling have successfully strengthened the reproducibility, persuasiveness, and biological relevance of our findings regarding Glud1 inhibition in HSCs. We also sincerely appreciate your validation of our tempered and reframed interpretation of the Glud1–STAT5a interaction. Your insightful guidance during the previous revision process was instrumental in helping us present a scientifically rigorous, well-balanced story that avoids overstatement while highlighting the novel correlative association.

In this round, we have taken your remaining conceptual comments with equal seriousness to further elevate the clarity and integration of our study.

But, despite these improvements, the central mechanistic framework of the study remains insufficiently defined. In the revised manuscript, multiple layers including metabolic changes αKG and NAD^+/NADH, the Glud1 and STAT5a interaction, and alterations in chromatin and transcriptional states are presented, but it is still unclear how these are causally integrated. As a result, while each dataset is individually interesting, the study does not yet converge on a single, coherent mechanistic explanation, leaving the overall message somewhat difficult to interpret.

We completely agree with the reviewer that establishing an integrated overview across the multiple layers of our data (metabolism, cytokine signaling, protein interaction, chromatin accessibility, transcription, and cell fate) is essential to provide a coherent conceptual framework. We sincerely appreciate this constructive criticism, which prompted us to reorganize our narrative and present our findings as a well-aligned model. To address this crucial point and provide a clear, integrated message without overstating a definitive mechanistic driver, we have constructed a comprehensive summary by visually aligning each dataset across these layers into a step-by-step

representation of correlated states, which has been added as Figure S8.

1. **Metabolism & Cytokine signaling (Figure S4A-B, Figure 5):** Under conventional conditions with increased intracellular glutamate (Glu) levels in HSCs, Glud1 elevates global αKG levels and decreases the NAD^+/NADH ratio, accompanying a physical interaction with STAT5a. Conversely, Glud1 inhibition decreases global αKG levels and increases the NAD^+/NADH ratio, while suppressing this interaction without negatively affecting STAT5 phosphorylation.
2. **Chromatin accessibility (Figure 4K-M, Figure S5Q-R):** Under robust JAK2-STAT5 signaling, the conventional conditions correlate with a loss of HSC-specific chromatin accessibility at potential STAT5-binding regions. In contrast, Glud1 inhibition closely parallels the maintenance of an open accessibility pattern at these regions.
3. **Transcriptome (Figure 4H, K-M, Figure S5Q-R):** These distinct chromatin architectures closely parallel the loss or maintenance of the HSC-specific gene expression signature.
4. **Cell fate (Figure 4J):** Ultimately, these aligned multi-layered states correspond to HSC fate outcomes under robust STAT activity. While conventional conditions progress a specific differentiation trajectory (Re-cluster 0→2→3), Glud1 inhibition block this trajectory, aligning with HSC self-renewal ($EPCR^{High}$ maintenance).

This integrated summary has resolved the previously disjointed interpretation by shifting the focus toward a parallel alignment of cellular states. The overall message of our study has now successfully converged on a coherent explanation: Glud1 inhibition under robust JAK2-STAT signaling—which suppresses the Glud1–STAT5a interaction—guides HSCs into self-renewal by blocking a specific differentiation trajectory in parallel with the preservation of HSC-specific chromatin accessibility at potential STAT5-binding regions and gene expression profiles. We have thoroughly revised the Discussion section (page 20, lines 461–468) to reflect this balanced framework and walk the reader through these aligned data layers. Moreover, we have added a brief explanation of the alternative differentiation trajectory in the subsequent sentence (page 20, lines 470–471).

A particularly important issue is that it remains unclear whether the effects of Glud1 inhibition reflect active instruction of cell fate or, alternatively, the selective preservation or expansion of a pre-existing HSC subset. This distinction has direct implications for the interpretation of the results and should be addressed either experimentally or through a more explicit and balanced discussion.

We completely agree with the reviewer that distinguishing between the active instruction of cell fate and the selective preservation or expansion of a pre-existing HSC subset is critical for correctly

interpreting our findings. To address this pivotal point, we first focused on our experimental evidence showing that the Glud1 inhibitor R162 increased EPCR^{High} HSC number by robustly maintaining the frequency of EPCR^{High} HSCs without affecting the total cell number after culture (Figure 3D, F and G). If HSC expansion was purely driven by accelerated proliferation of whole cultured cells or the selective expansion of a pre-existing subset, a corresponding increase in total cell cellularity would be expected. Moreover, our single-cell multiome (scMultiome) analysis indicated that R162 treatment increases the number of cells belonging to Re-cluster 0 (the most primitive cluster) specifically by suppressing a particular differentiation trajectory (Re-clusters 0→2→3) (Figures 4E–J). Taken together, these data strongly suggest that Glud1 inhibition contributes to HSC expansion by actively guiding HSCs into self-renewal through blocking a specific differentiation trajectory, rather than merely modulating general cell division activity. We have added a detailed discussion regarding this active fate instruction framework in the revised manuscript (page 16, lines 361–370).

Simultaneously, to ensure a balanced interpretation as suggested, we noted that Glud1 inhibition did not affect the alternative differentiation trajectory (Re-clusters 0→1→4) (Figures 4E–J). Importantly, the beneficial effect of Glud1 inhibition on HSC maintenance is dependent on robust JAK2-STAT signaling (Figure 5A-F). Moreover, cells on the 0→2→3 trajectory displayed a significantly higher enrichment of STAT-binding motifs within open chromatin regions compared to those on the 0→1→4 trajectory (Figure 4K). These findings cannot entirely exclude the possibility that Glud1 inhibition preferentially acts upon or expands a specific HSC subset that inherently maintains high JAK–STAT activity throughout the culture. To maintain scientific rigor, we have reinforced this explicit and balanced discussion regarding the alternative possibility of selective preservation/expansion in the revised Discussion section (page 16, lines 370–382).

In conjunction with the addition of these detailed discussions, we have also comprehensively revised the final paragraph of the Discussion section to avoid redundant duplication and to clear a path forward for future combinations with other culture systems (page 20, lines 473–475).

In addition, the conclusion that the observed effects are independent of αKG appears to be based primarily on the exclusion of global metabolic changes, without sufficiently considering the possibility of localized or complex-dependent metabolic regulation.

We completely agree with the reviewer that the exclusion of global metabolic shifts does not rule out the possibility of localized or complex-dependent metabolic regulation. While our conclusion regarding the independence of αKG was based on global metabolic changes and the lack of rescue by exogenous dimethyl-αKG (dmαKG), we fully recognize the validity of the reviewer's point regarding compartmentalized metabolic microenvironments. To address this crucial concept and ensure a well-balanced perspective, we have explicitly added the qualifier "global" to our conclusion regarding the independence of αKG in the revised manuscript to clarify the scope of our

observation. Moreover, we have explicitly expanded our discussion on the possibility of localized or protein-complex-dependent metabolic regulation in the revised Discussion section (page 17, lines 406–page 17, lines 414). Specifically, we highlight a previously reported paradigm where Glud1 physically interacts with a specific protein complex (such as the NF-κB complex), allowing locally generated αKG to specifically bind and activate its target (e.g., IKKβ)—a localized effect that inherently cannot be mimicked by the simple addition of exogenous dmαKG (Wang et al., Mol Cell, 2019; Ref. 26). By incorporating this explicitly nuanced discussion, we have balanced our interpretation to acknowledge that while global αKG abundance is excluded as a main driver, localized or complex-dependent metabolic regulation remains a highly plausible and intriguing possibility.

Reviewer #2 (Remarks to the Author):

Thank you to the authors for responding to my comments and suggestions. The manuscript greatly improved upon revision. Just a few remarks in response:

We are sincerely grateful to the reviewer for the highly encouraging assessment and for recognizing the substantial improvements in our revised manuscript. The insightful comments and constructive suggestions provided by the reviewer during the previous revision loop were truly instrumental in strengthening the quality, rigor, and overall clarity of our study. We have addressed your remaining remarks with the utmost seriousness and care, further refining the text to ensure the highest scientific standard. Below, we provide our point-by-point responses to your specific comments.

- Regarding my point to Figure 3B-F: if the absolute pool of EPCRlow cells is reduced, is the composition within this pool the same? Do you have similar levels of the more committed progenitors or is there a bias towards one of the other? EPCRlow cells is a great mix of different cell populations.

Thank you for this highly insightful question. As you correctly point out, the $EPCR^{low/-}$ population represents a highly heterogeneous mix of various downstream progenitors. Based on our date, we suggest that the reduced absolute size of the $EPCR^{low/-}$ pool induced by the Glud1 inhibitor R162 is not a uniform or non-specific decrease across all cell types; rather, there is a distinct compositional bias within this population. Indeed, our single-cell multiome (scMultiome) analysis indicated that R162 treatment specifically suppresses the differentiation trajectory heading toward the MPP2/3/4 states (Re-clusters 0→2→3) (Figures 4E–J). In sharp contrast, the alternative

differentiation pathway (Re-clusters 0→1→4) remains functional (Figures 4E–J). Furthermore, cells on the 0→2→3 trajectory displayed a significantly higher enrichment of STAT-binding motifs within open chromatin regions compared to those on the 0→1→4 trajectory (Figure 4K). These layered datasets strongly indicate that R162 treatment preferentially decreases progenitors associated with the JAK–STAT-dependent differentiation branch (Re-clusters 2 and 3), leading to a clear compositional shift (bias) that relatively preserves the alternative branches (Re-clusters 1 and 4). Therefore, the reduction of the overall $EPCR^{low/-}$ pool reflects a highly regulated, trajectory-specific blockade of differentiation rather than a generalized suppression of all committed progenitors. To ensure this clear conceptual framework is explicitly conveyed to the readers, we have incorporated this discussion into the revised Discussion section (page 16, lines 361–382).

- The additional detailed analysis on Glud1-STAT5a in figures 4 and 5 looks really interesting. As the authors conclude it does not uncover the whole mechanism yet, but it definitely increases the impact of their story

We are deeply encouraged by the reviewer's positive and supportive feedback regarding our additional analyses of the Glud1–STAT5a relationship presented in Figures 4 and 5. We completely agree that while these findings do not yet fully dissect the entire, detailed molecular cascade, establishing this direct biochemical and correlative link significantly elevates the conceptual novelty and overall impact of our study. As you noted, we have intentionally maintained a balanced and tempered interpretation of these data in the manuscript to ensure scientific rigor. We believe that highlighting this interaction provides a crucial foundation for future research exploring localized metabolic-epigenetic cross-talk in stem cell biology. Thank you very much for recognizing the value and broader implications of this newly added narrative.

- The additional data on ERK inhibition still does not formally proof the point raised regarding the potential confounding effect. However, this will also be a difficult one to dissect.

We sincerely appreciate the reviewer's professional and balanced understanding regarding the inherent complexity of dissecting these signaling networks. As you insightfully noted, completely isolating the individual confounding effects remains a major technical and biological challenge. While we agree that our current ERK inhibition data may not constitute a definitive formal proof, we believe it provides a meaningful direction toward understanding the signaling axis. To maintain scientific rigor and prevent any overstatement, we have appropriately tempered our conclusion and included a nuanced discussion acknowledging these complexities and potential alternative interpretations in the revised manuscript (page 13, lines 296–299). Thank you again for your constructive guidance.

Reviewer #3 (Remarks to the Author):

Hashimoto et al. have substantially revised their manuscript on Glud1, addressing all of my comments. I am convinced that the strength of the findings has been improved by the modifications. Simultaneously, I think the authors have tempered comments where the evidence was less robust. Still, I think there is substantial cross validation of the key results with various approaches. Thus, I remain convinced about the main conclusions and think that Glud1 inhibition. My main reservation is the lack of validation in human cultures, but the authors discuss a reasonable path forward for progress in translation. Overall, I think the manuscript has been improved leaps and bounds and the impact of the findings warrant publication and discussion by the community.

We are profoundly grateful to the reviewer for such an exceptionally high evaluation and for the strong endorsement of our study for publication. It is incredibly rewarding to receive your validation that the manuscript has improved "leaps and bounds" and that our extensive modifications, including the multi-approach cross-validations and the appropriately tempered interpretations, have successfully solidified the main conclusions. Your rigorous yet constructive guidance during the previous loop was vital in elevating our story to this standard. We also sincerely appreciate your encouraging feedback regarding our translational discussion. As you insightfully pointed out, while the lack of direct validation in human cultures remains a current limitation, we are fully committed to pursuing this "path forward." We believe that translating these findings into human HSC biology is a crucial next milestone, and your recognition of our proposed approach gives us great confidence as we move into the next phase of this research. Thank you once again for recognizing the scientific impact of our work and for advocating its value to the broader scientific community.

We sincerely thank the Editor and both Reviewers for their thoughtful evaluation of our manuscript and for their supportive final comments. We are deeply grateful for the reviewers' insightful feedback throughout the review process, which has significantly enhanced the quality and clarity of our study.

Below are our responses to the final comments from the reviewers.

Reviewer #1 (Remarks to the Author):

The revised MS has reached a standard where it provides useful information to the readers of this journal.

We sincerely thank Reviewer #1 for the positive evaluation and for endorsing our revised manuscript for publication in *Nature Communications*. We greatly appreciate the time and constructive feedback provided throughout the revision process, which helped us improve our manuscript.

Reviewer #2 (Remarks to the Author):

Thank you to the authors for responding to my remarks. This is an interesting manuscript that will attract great attention in the field.

We are deeply grateful to Reviewer #2 for these highly encouraging words and for the supportive assessment of our work. We sincerely appreciate the insightful comments and suggestions provided during the review process, which contributed substantially to refining our study.
